# Supplementary material for: One Year of SARS-CoV-2: Genomic Characterization of COVID-19 Outbreak in Qatar
Source: Front Cell Infect Microbiol. 2021 Nov 17;11:768883. doi: 10.3389/fcimb.2021.768883 (PMC8637114; doi:10.3389/fcimb.2021.768883)
Supplement: Supplementary file 1 [file DataSheet_1.pdf]

Supplementary Material

1 Supplementary Data 1

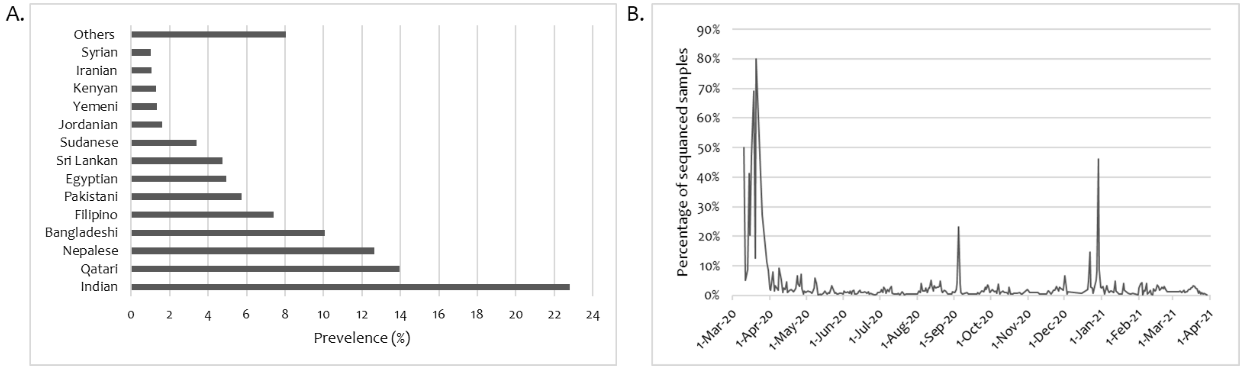

Supplementary Figure.1. Nationalities and the frequency of sequenced samples in the studies cohort. (A) Nationalities observed at least at 1% prevalence. Others (<1%) includes: Algerian, American, Argentinean, Australian, Bahraini, Brazilian, British, Cameroonian, Canadian, Chinese, Cuban, Cypriot, Djiboutian, Dominican, Emirati, Eritrean, Ethiopian, French, Gambian, German, Ghanaian, Indonesian, Irish, Italian, Lebanese, Malaysian, Malian, Moroccan, Myanmar, Nepalese, Nigerian, Omani, Palestinian, Portuguese, Romanian, Russian, Saudi, Serbian, Somali, South African, South Korean, Spanish, Sudanese, Swiss, Tajik, Thai, Tunisian, Turkish, Ugandan, and Ukrainian. **(B)** Percentage of sequenced samples in reference to the daily COVID19 positive cases in Qatar.

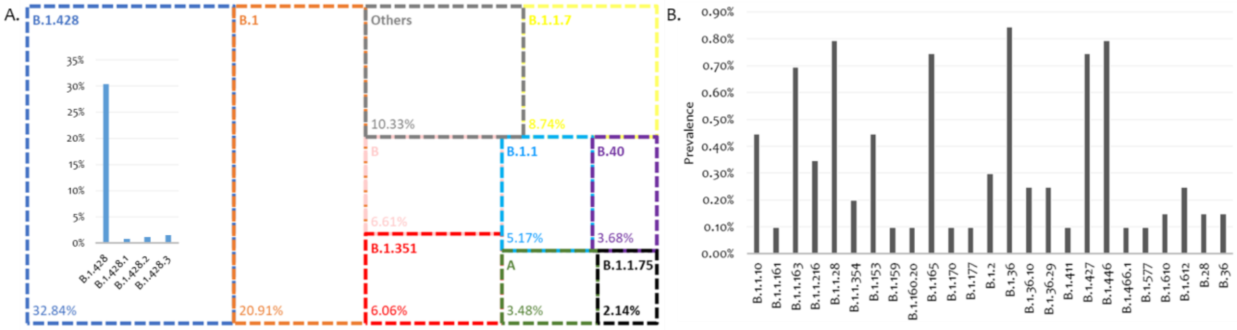

Supplementary Figure.2. Lineages detected in Qatar's sequenced cohort. (A) Highly prevalent lineages (>1%) and (B) Low prevalent lineages (<1% and in at least two sequences). Nomenclature is based on Pangolin tool.

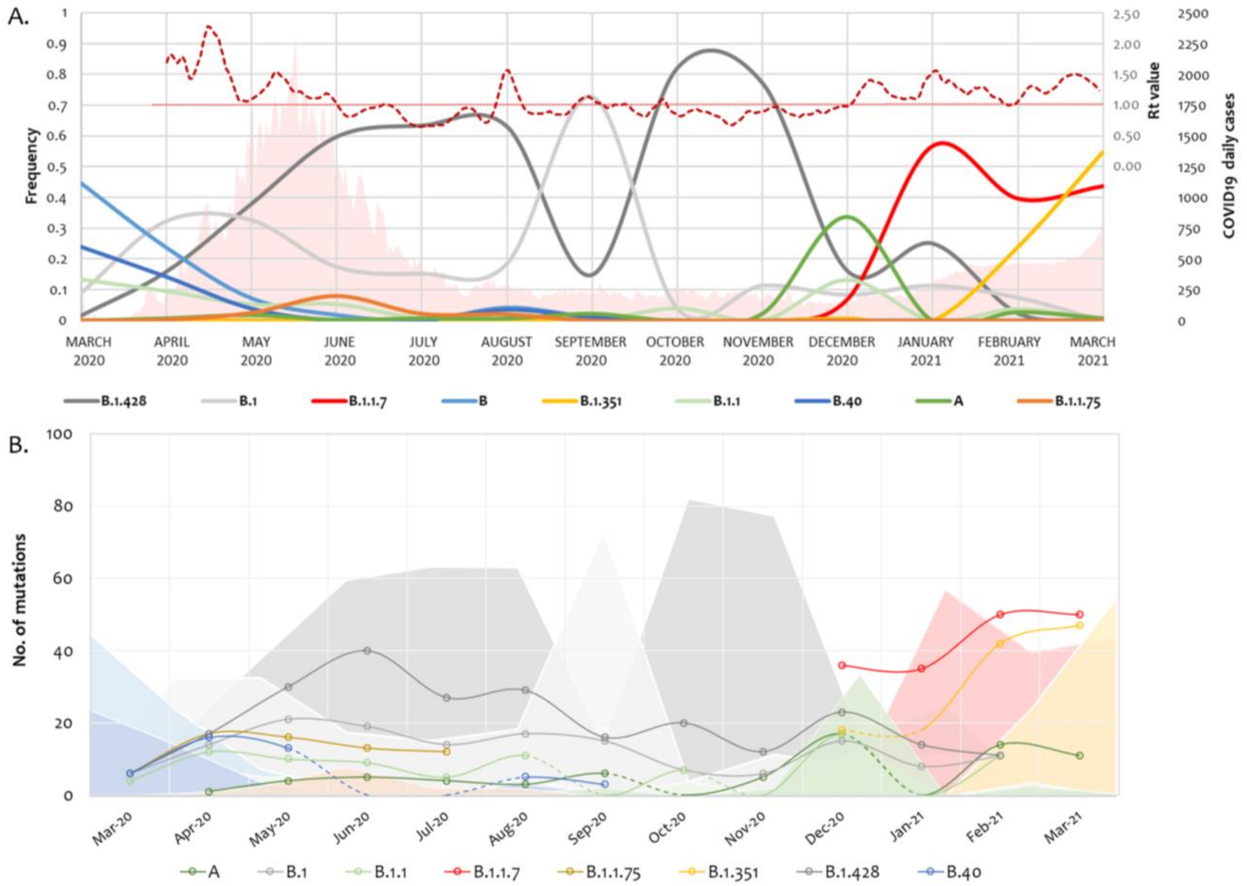

Supplementary Figure.3. SARS-COV-2 lineages evolution over the one-year study period (March 2020-March 2021). (A) Frequency of the highly prevalent lineages across the one year period in reference to COVID19 daily positive cases (area in light pink) and the Effective Reproduction Number (Rt, in dotted deep red line). The light red line highlights an Rt value of 1. **(B)** Accumulation of non-synonymous mutations in the genomes of major SARS-CoV-2 lineages.

2 Supplementary Data 2

Virus name	Accession ID
hCoV-19/Qatar/QA-QU_01-11/2020	EPI_ISL_1712659
hCoV-19/Qatar/QA-QU_01-12/2020	EPI_ISL_1712660
hCoV-19/Qatar/QA-QU_01-17/2020	EPI_ISL_1712662
hCoV-19/Qatar/QA-QU_01-21/2020	EPI_ISL_1712665
hCoV-19/Qatar/QA-QU_01-23/2020	EPI_ISL_1712667
hCoV-19/Qatar/QA-QU_01-24/2020	EPI_ISL_1712668
hCoV-19/Qatar/QA-QU_01-5/2020	EPI_ISL_1712669
hCoV-19/Qatar/QA-QU_01-19/2020	EPI_ISL_1712663
hCoV-19/Qatar/QA-QU_02-2-44/2020	EPI_ISL_1712699
hCoV-19/Qatar/QA-QU_02-2-60/2020	EPI_ISL_1712705
hCoV-19/Qatar/QA-QU_02-3-36/2020	EPI_ISL_1712717
hCoV-19/Qatar/QA-QU_01-13/2020	EPI_ISL_1712661
hCoV-19/Qatar/QA-QU_01-2/2020	EPI_ISL_1712664
hCoV-19/Qatar/QA-QU_02-2-7/2020	EPI_ISL_1712706
hCoV-19/Qatar/QA-QU_02-3-14/2020	EPI_ISL_1712710
hCoV-19/Qatar/QA-QU_02-3-43/2020	EPI_ISL_1712720
hCoV-19/Qatar/QA-QU_01-22/2020	EPI_ISL_1712666
hCoV-19/Qatar/QA-QU_02-2-18/2020	EPI_ISL_1712686
hCoV-19/Qatar/QA-QU_02-2-22/2020	EPI_ISL_1712689
hCoV-19/Qatar/QA-QU_02-2-27/2020	EPI_ISL_1712691
hCoV-19/Qatar/QA-QU_02-2-35/2020	EPI_ISL_1712695
hCoV-19/Qatar/QA-QU_02-2-5/2020	EPI_ISL_1712702
hCoV-19/Qatar/QA-QU_02-2-59/2020	EPI_ISL_1712703
hCoV-19/Qatar/QA-QU_02-3-16/2020	EPI_ISL_1712712
hCoV-19/Qatar/QA-QU_02-3-33/2020	EPI_ISL_1712715
hCoV-19/Qatar/QA-QU_02-3-37/2020	EPI_ISL_1712718
hCoV-19/Qatar/QA-QU_02-2-29/2020	EPI_ISL_1712692
hCoV-19/Qatar/QA-QU_02-2-39/2020	EPI_ISL_1712696
hCoV-19/Qatar/QA-QU_02-2-4/2020	EPI_ISL_1712697
hCoV-19/Qatar/QA-QU_02-2-49/2020	EPI_ISL_1712701
hCoV-19/Qatar/QA-QU_02-2-6/2020	EPI_ISL_1712704
hCoV-19/Qatar/QA-QU_02-3-11/2020	EPI_ISL_1712708
hCoV-19/Qatar/QA-QU_02-3-28/2020	EPI_ISL_1712713
hCoV-19/Qatar/QA-QU_02-3-32/2020	EPI_ISL_1712714
hCoV-19/Qatar/QA-QU_02-3-45/2020	EPI_ISL_1712721
hCoV-19/Qatar/QA-QU_02-2-1/2020	EPI_ISL_1712684
hCoV-19/Qatar/QA-QU_02-2-10/2020	EPI_ISL_1712685

hCoV-19/Qatar/QA-QU_02-2-20/2020	EPI_ISL_1712687
hCoV-19/Qatar/QA-QU_02-2-30/2020	EPI_ISL_1712693
hCoV-19/Qatar/QA-QU_02-2-31/2020	EPI_ISL_1712694
hCoV-19/Qatar/QA-QU_02-2-40/2020	EPI_ISL_1712698
hCoV-19/Qatar/QA-QU_02-2-9/2020	EPI_ISL_1712707
hCoV-19/Qatar/QA-QU_02-3-13/2020	EPI_ISL_1712709
hCoV-19/Qatar/QA-QU_02-3-15/2020	EPI_ISL_1712711
hCoV-19/Qatar/QA-QU_02-3-42/2020	EPI_ISL_1712719
hCoV-19/Qatar/QA-QU_02-3-50/2020	EPI_ISL_1712723
hCoV-19/Qatar/QA-QU_02-3-51/2020	EPI_ISL_1712724
hCoV-19/Qatar/QA-QU_02-3-58/2020	EPI_ISL_1712727
hCoV-19/Qatar/QA-QU_02-2-21/2020	EPI_ISL_1712688
hCoV-19/Qatar/QA-QU_02-3-47/2020	EPI_ISL_1712722
hCoV-19/Qatar/QA-QU_02-3-52/2020	EPI_ISL_1712725
hCoV-19/Qatar/QA-QU_02-3-54/2020	EPI_ISL_1712726
hCoV-19/Qatar/QA-QU_02-3-61/2020	EPI_ISL_1712728
hCoV-19/Qatar/QA-QU_02-2-46/2020	EPI_ISL_1712700
hCoV-19/Qatar/QA-QU_01-8/2020	EPI_ISL_1712670
hCoV-19/Qatar/QA-QU_02-2-26/2020	EPI_ISL_1712690
hCoV-19/Qatar/QA-QU_02-3-34/2020	EPI_ISL_1712716
hCoV-19/Qatar/QA-QU_09-1-E3/2020	EPI_ISL_1713096
hCoV-19/Qatar/QA-QU_09-1-G8/2020	EPI_ISL_1713115
hCoV-19/Qatar/QA-QU_09-1-H2/2020	EPI_ISL_1713117
hCoV-19/Qatar/QA-QU_09-1-A7/2020	EPI_ISL_1713078
hCoV-19/Qatar/QA-QU_09-1-G1/2020	EPI_ISL_1713110
hCoV-19/Qatar/QA-WCMQ_FD18215049/2020	EPI_ISL_1714648
hCoV-19/Qatar/QA-QU_02-1-1/2020	EPI_ISL_1712671
hCoV-19/Qatar/QA-QU_02-1-7/2020	EPI_ISL_1712683
hCoV-19/Qatar/QA-QU_09-1-E10/2020	EPI_ISL_1713094
hCoV-19/Qatar/QA-QU_09-1-H8/2020	EPI_ISL_1713121
hCoV-19/Qatar/QA-QU_02-1-13/2020	EPI_ISL_1712672
hCoV-19/Qatar/QA-QU_02-1-4/2020	EPI_ISL_1712680
hCoV-19/Qatar/QA-QU_03-H9/2020	EPI_ISL_1712786
hCoV-19/Qatar/QA-QU_02-1-14/2020	EPI_ISL_1712673
hCoV-19/Qatar/QA-QU_02-1-17/2020	EPI_ISL_1712674
hCoV-19/Qatar/QA-QU_02-1-5/2020	EPI_ISL_1712681
hCoV-19/Qatar/QA-WCMQ_FD15651317/2020	EPI_ISL_1714134
hCoV-19/Qatar/QA-QU_02-1-18/2020	EPI_ISL_1712675
hCoV-19/Qatar/QA-QU_02-1-6/2020	EPI_ISL_1712682

hCoV-19/Qatar/QA-QU_02-1-23/2020	EPI_ISL_1712677
hCoV-19/Qatar/QA-QU_02-1-25/2020	EPI_ISL_1712678
hCoV-19/Qatar/QA-QU_03-A11/2020	EPI_ISL_1712731
hCoV-19/Qatar/QA-QU_09-1-A2/2020	EPI_ISL_1713074
hCoV-19/Qatar/QA-QU_09-1-A9/2020	EPI_ISL_1713080
hCoV-19/Qatar/QA-QU_09-1-F3/2020	EPI_ISL_1713104
hCoV-19/Qatar/QA-QU_09-1-F4/2020	EPI_ISL_1713105
hCoV-19/Qatar/QA-QU_09-1-F7/2020	EPI_ISL_1713107
hCoV-19/Qatar/QA-QU_09-1-F8/2020	EPI_ISL_1713108
hCoV-19/Qatar/QA-QU_09-1-H12/2020	EPI_ISL_1713116
hCoV-19/Qatar/QA-QU_09-1-H6/2020	EPI_ISL_1713119
hCoV-19/Qatar/QA-QU_02-1-2/2020	EPI_ISL_1712676
hCoV-19/Qatar/QA-QU_02-1-3/2020	EPI_ISL_1712679
hCoV-19/Qatar/QA-QU_03-B12/2020	EPI_ISL_1712741
hCoV-19/Qatar/QA-QU_09-1-A6/2020	EPI_ISL_1713077
hCoV-19/Qatar/QA-QU_09-1-F12/2020	EPI_ISL_1713102
hCoV-19/Qatar/QA-WCMQ_FD15506506/2020	EPI_ISL_1713954
hCoV-19/Qatar/QA-WCMQ_FD15506537/2020	EPI_ISL_1713985
hCoV-19/Qatar/QA-WCMQ_FD15506566/2020	EPI_ISL_1714014
hCoV-19/Qatar/QA-QU_12-4-B9/2020	EPI_ISL_1713288
hCoV-19/Qatar/QA-WCMQ_FD15506502/2020	EPI_ISL_1713950
hCoV-19/Qatar/QA-WCMQ_FD15506540/2020	EPI_ISL_1713988
hCoV-19/Qatar/QA-WCMQ_FD15506521/2020	EPI_ISL_1713969
hCoV-19/Qatar/QA-WCMQ_FD18272693/2020	EPI_ISL_1714686
hCoV-19/Qatar/QA-QU_09-1-G6/2020	EPI_ISL_1713113
hCoV-19/Qatar/QA-WCMQ_FD15506504/2020	EPI_ISL_1713952
hCoV-19/Qatar/QA-WCMQ_FD15506525/2020	EPI_ISL_1713973
hCoV-19/Qatar/QA-WCMQ_FD18272684/2020	EPI_ISL_1714678
hCoV-19/Qatar/QA-WCMQ_FD18365000/2020	EPI_ISL_1714745
hCoV-19/Qatar/QA-QU_09-1-C8/2020	EPI_ISL_1713090
hCoV-19/Qatar/QA-WCMQ_FD18215263/2020	EPI_ISL_1714649
hCoV-19/Qatar/QA-QU_03-H12/2020	EPI_ISL_1712782

hCoV-19/Qatar/QA-QU_09-1-A8/2020	EPI_ISL_1713079
hCoV-19/Qatar/QA-QU_09-1-C10/2020	EPI_ISL_1713086
hCoV-19/Qatar/QA-QU_09-1-E8/2020	EPI_ISL_1713098
hCoV-19/Qatar/QA-QU_09-1-F10/2020	EPI_ISL_1713100
hCoV-19/Qatar/QA-QU_09-2-E3/2020	EPI_ISL_1713131
hCoV-19/Qatar/QA-QU_09-1-B1/2020	EPI_ISL_1713081
hCoV-19/Qatar/QA-QU_09-1-D8/2020	EPI_ISL_1713092
hCoV-19/Qatar/QA-WCMQ_FD18365042/2020	EPI_ISL_1714746
hCoV-19/Qatar/QA-QU_09-1-E7/2020	EPI_ISL_1713097
hCoV-19/Qatar/QA-QU_09-1-G7/2020	EPI_ISL_1713114
hCoV-19/Qatar/QA-WCMQ_FD15506552/2020	EPI_ISL_1714000
hCoV-19/Qatar/QA-QU_09-1-B3/2020	EPI_ISL_1713083
hCoV-19/Qatar/QA-QU_09-1-C7/2020	EPI_ISL_1713089
hCoV-19/Qatar/QA-WCMQ_FD15506541/2020	EPI_ISL_1713989
hCoV-19/Qatar/QA-WCMQ_FD18270879/2020	EPI_ISL_1714673
hCoV-19/Qatar/QA-QU_09-1-G4/2020	EPI_ISL_1713112
hCoV-19/Qatar/QA-QU_09-2-B12/2020	EPI_ISL_1713123
hCoV-19/Qatar/QA-WCMQ_FD15506547/2020	EPI_ISL_1713995
hCoV-19/Qatar/QA-WCMQ_FD15506578/2020	EPI_ISL_1714026
hCoV-19/Qatar/QA-WCMQ_FD15506581/2020	EPI_ISL_1714029
hCoV-19/Qatar/QA-WCMQ_FD15506582/2020	EPI_ISL_1714030
hCoV-19/Qatar/QA-WCMQ_FD15506584/2020	EPI_ISL_1714032
hCoV-19/Qatar/QA-QU_09-1-E9/2020	EPI_ISL_1713099
hCoV-19/Qatar/QA-QU_09-1-H7/2020	EPI_ISL_1713120
hCoV-19/Qatar/QA-QU_09-2-C4/2020	EPI_ISL_1713127
hCoV-19/Qatar/QA-WCMQ_FD15506507/2020	EPI_ISL_1713955
hCoV-19/Qatar/QA-WCMQ_FD15506534/2020	EPI_ISL_1713982
hCoV-19/Qatar/QA-WCMQ_FD15506546/2020	EPI_ISL_1713994
hCoV-19/Qatar/QA-WCMQ_FD15506567/2020	EPI_ISL_1714015
hCoV-19/Qatar/QA-WCMQ_FD18272703/2020	EPI_ISL_1714695

hCoV-19/Qatar/QA-QU_09-1-A11/2020	EPI_ISL_1713072
hCoV-19/Qatar/QA-QU_09-2-C9/2020	EPI_ISL_1713128
hCoV-19/Qatar/QA-QU_09-2-H1/2020	EPI_ISL_1713137
hCoV-19/Qatar/QA-WCMQ_FD15530973/2020	EPI_ISL_1714047
hCoV-19/Qatar/QA-WCMQ_FD18272690/2020	EPI_ISL_1714683
hCoV-19/Qatar/QA-WCMQ_FD18272767/2020	EPI_ISL_1714739
hCoV-19/Qatar/QA-QU_09-1-E12/2020	EPI_ISL_1713095
hCoV-19/Qatar/QA-QU_09-1-F6/2020	EPI_ISL_1713106
hCoV-19/Qatar/QA-WCMQ_FD15506523/2020	EPI_ISL_1713971
hCoV-19/Qatar/QA-WCMQ_FD15506558/2020	EPI_ISL_1714006
hCoV-19/Qatar/QA-WCMQ_FD15506577/2020	EPI_ISL_1714025
hCoV-19/Qatar/QA-WCMQ_FD15533261/2020	EPI_ISL_1714048
hCoV-19/Qatar/QA-WCMQ_FD18272719/2020	EPI_ISL_1714705
hCoV-19/Qatar/QA-QU_04-B3/2020	EPI_ISL_1712800
hCoV-19/Qatar/QA-QU_09-1-B7/2020	EPI_ISL_1713084
hCoV-19/Qatar/QA-QU_09-1-G3/2020	EPI_ISL_1713111
hCoV-19/Qatar/QA-WCMQ_FD15506515/2020	EPI_ISL_1713963
hCoV-19/Qatar/QA-WCMQ_FD15506522/2020	EPI_ISL_1713970
hCoV-19/Qatar/QA-WCMQ_FD15506524/2020	EPI_ISL_1713972
hCoV-19/Qatar/QA-WCMQ_FD15506549/2020	EPI_ISL_1713997
hCoV-19/Qatar/QA-WCMQ_FD15506550/2020	EPI_ISL_1713998
hCoV-19/Qatar/QA-WCMQ_FD15506580/2020	EPI_ISL_1714028
hCoV-19/Qatar/QA-WCMQ_FD15506585/2020	EPI_ISL_1714033
hCoV-19/Qatar/QA-WCMQ_FD15506590/2020	EPI_ISL_1714038
hCoV-19/Qatar/QA-WCMQ_FD15540726/2020	EPI_ISL_1714067
hCoV-19/Qatar/QA-QU_03-B11/2020	EPI_ISL_1712740
hCoV-19/Qatar/QA-QU_09-1-A3/2020	EPI_ISL_1713075
hCoV-19/Qatar/QA-QU_09-2-E12/2020	EPI_ISL_1713130

hCoV-19/Qatar/QA-WCMQ_FD15506503/2020	EPI_ISL_1713951
hCoV-19/Qatar/QA-WCMQ_FD15506510/2020	EPI_ISL_1713958
hCoV-19/Qatar/QA-WCMQ_FD15506512/2020	EPI_ISL_1713960
hCoV-19/Qatar/QA-WCMQ_FD15506520/2020	EPI_ISL_1713968
hCoV-19/Qatar/QA-WCMQ_FD15506526/2020	EPI_ISL_1713974
hCoV-19/Qatar/QA-WCMQ_FD15506528/2020	EPI_ISL_1713976
hCoV-19/Qatar/QA-WCMQ_FD15506531/2020	EPI_ISL_1713979
hCoV-19/Qatar/QA-WCMQ_FD15506543/2020	EPI_ISL_1713991
hCoV-19/Qatar/QA-WCMQ_FD15506544/2020	EPI_ISL_1713992
hCoV-19/Qatar/QA-WCMQ_FD15506545/2020	EPI_ISL_1713993
hCoV-19/Qatar/QA-WCMQ_FD15506548/2020	EPI_ISL_1713996
hCoV-19/Qatar/QA-WCMQ_FD15506553/2020	EPI_ISL_1714001
hCoV-19/Qatar/QA-WCMQ_FD15506560/2020	EPI_ISL_1714008
hCoV-19/Qatar/QA-WCMQ_FD15506562/2020	EPI_ISL_1714010
hCoV-19/Qatar/QA-WCMQ_FD15506572/2020	EPI_ISL_1714020
hCoV-19/Qatar/QA-WCMQ_FD15506573/2020	EPI_ISL_1714021
hCoV-19/Qatar/QA-WCMQ_FD15506575/2020	EPI_ISL_1714023
hCoV-19/Qatar/QA-WCMQ_FD15506576/2020	EPI_ISL_1714024
hCoV-19/Qatar/QA-WCMQ_FD15506583/2020	EPI_ISL_1714031
hCoV-19/Qatar/QA-WCMQ_FD15506587/2020	EPI_ISL_1714035
hCoV-19/Qatar/QA-WCMQ_FD15506588/2020	EPI_ISL_1714036
hCoV-19/Qatar/QA-WCMQ_FD15540695/2020	EPI_ISL_1714055
hCoV-19/Qatar/QA-WCMQ_FD15540712/2020	EPI_ISL_1714059

hCoV-19/Qatar/QA-WCMQ_FD15540718/2020	EPI_ISL_1714065
hCoV-19/Qatar/QA-WCMQ_FD18160888/2020	EPI_ISL_1714357
hCoV-19/Qatar/QA-WCMQ_FD18216975/2020	EPI_ISL_1714665
hCoV-19/Qatar/QA-WCMQ_FD18272725/2020	EPI_ISL_1714710
hCoV-19/Qatar/QA-WCMQ_FD18272726/2020	EPI_ISL_1714711
hCoV-19/Qatar/QA-WCMQ_FD18272734/2020	EPI_ISL_1714718
hCoV-19/Qatar/QA-QU_04-B2/2020	EPI_ISL_1712799
hCoV-19/Qatar/QA-QU_09-1-B9/2020	EPI_ISL_1713085
hCoV-19/Qatar/QA-QU_09-1-E1/2020	EPI_ISL_1713093
hCoV-19/Qatar/QA-QU_09-2-B10/2020	EPI_ISL_1713122
hCoV-19/Qatar/QA-QU_09-2-F3/2020	EPI_ISL_1713136
hCoV-19/Qatar/QA-QU_09-2-H3/2020	EPI_ISL_1713138
hCoV-19/Qatar/QA-WCMQ_FD15506505/2020	EPI_ISL_1713953
hCoV-19/Qatar/QA-WCMQ_FD15506511/2020	EPI_ISL_1713959
hCoV-19/Qatar/QA-WCMQ_FD15506513/2020	EPI_ISL_1713961
hCoV-19/Qatar/QA-WCMQ_FD15506514/2020	EPI_ISL_1713962
hCoV-19/Qatar/QA-WCMQ_FD15506518/2020	EPI_ISL_1713966
hCoV-19/Qatar/QA-WCMQ_FD15506529/2020	EPI_ISL_1713977
hCoV-19/Qatar/QA-WCMQ_FD15506530/2020	EPI_ISL_1713978
hCoV-19/Qatar/QA-WCMQ_FD15506542/2020	EPI_ISL_1713990
hCoV-19/Qatar/QA-WCMQ_FD15506574/2020	EPI_ISL_1714022
hCoV-19/Qatar/QA-WCMQ_FD15506579/2020	EPI_ISL_1714027
hCoV-19/Qatar/QA-WCMQ_FD15506589/2020	EPI_ISL_1714037
hCoV-19/Qatar/QA-WCMQ_FD15506591/2020	EPI_ISL_1714039
hCoV-19/Qatar/QA-WCMQ_FD15506593/2020	EPI_ISL_1714041
hCoV-19/Qatar/QA-WCMQ_FD15506597/2020	EPI_ISL_1714045

hCoV-19/Qatar/QA-WCMQ_FD15617953/2020	EPI_ISL_1714109
hCoV-19/Qatar/QA-WCMQ_FD15617970/2020	EPI_ISL_1714123
hCoV-19/Qatar/QA-WCMQ_FD15617972/2020	EPI_ISL_1714124
hCoV-19/Qatar/QA-WCMQ_FD18160883/2020	EPI_ISL_1714356
hCoV-19/Qatar/QA-WCMQ_FD18272687/2020	EPI_ISL_1714681
hCoV-19/Qatar/QA-WCMQ_FD18272695/2020	EPI_ISL_1714688
hCoV-19/Qatar/QA-WCMQ_FD18272715/2020	EPI_ISL_1714703
hCoV-19/Qatar/QA-QU_12-4-B8/2020	EPI_ISL_1713287
hCoV-19/Qatar/QA-WCMQ_FD15506516/2020	EPI_ISL_1713964
hCoV-19/Qatar/QA-WCMQ_FD15506527/2020	EPI_ISL_1713975
hCoV-19/Qatar/QA-WCMQ_FD15506561/2020	EPI_ISL_1714009
hCoV-19/Qatar/QA-WCMQ_FD15617909/2020	EPI_ISL_1714088
hCoV-19/Qatar/QA-WCMQ_FD15617926/2020	EPI_ISL_1714097
hCoV-19/Qatar/QA-WCMQ_FD15617927/2020	EPI_ISL_1714098
hCoV-19/Qatar/QA-WCMQ_FD15617929/2020	EPI_ISL_1714099
hCoV-19/Qatar/QA-WCMQ_FD15617933/2020	EPI_ISL_1714101
hCoV-19/Qatar/QA-WCMQ_FD15617938/2020	EPI_ISL_1714103
hCoV-19/Qatar/QA-WCMQ_FD15617939/2020	EPI_ISL_1714104
hCoV-19/Qatar/QA-WCMQ_FD15617942/2020	EPI_ISL_1714105
hCoV-19/Qatar/QA-WCMQ_FD15617946/2020	EPI_ISL_1714106
hCoV-19/Qatar/QA-WCMQ_FD15617955/2020	EPI_ISL_1714111
hCoV-19/Qatar/QA-WCMQ_FD15617966/2020	EPI_ISL_1714119
hCoV-19/Qatar/QA-WCMQ_FD15617967/2020	EPI_ISL_1714120

hCoV-19/Qatar/QA-WCMQ_FD15617968/2020	EPI_ISL_1714121
hCoV-19/Qatar/QA-WCMQ_FD18272685/2020	EPI_ISL_1714679
hCoV-19/Qatar/QA-WCMQ_FD18272686/2020	EPI_ISL_1714680
hCoV-19/Qatar/QA-WCMQ_FD18272689/2020	EPI_ISL_1714682
hCoV-19/Qatar/QA-WCMQ_FD18272718/2020	EPI_ISL_1714704
hCoV-19/Qatar/QA-WCMQ_FD18272723/2020	EPI_ISL_1714709
hCoV-19/Qatar/QA-WCMQ_FD18272735/2020	EPI_ISL_1714719
hCoV-19/Qatar/QA-QU_09-1-B12/2020	EPI_ISL_1713082
hCoV-19/Qatar/QA-QU_09-1-F9/2020	EPI_ISL_1713109
hCoV-19/Qatar/QA-QU_12-4-A12/2020	EPI_ISL_1713286
hCoV-19/Qatar/QA-WCMQ_FD15506532/2020	EPI_ISL_1713980
hCoV-19/Qatar/QA-WCMQ_FD15506533/2020	EPI_ISL_1713981
hCoV-19/Qatar/QA-WCMQ_FD15506536/2020	EPI_ISL_1713984
hCoV-19/Qatar/QA-WCMQ_FD15506559/2020	EPI_ISL_1714007
hCoV-19/Qatar/QA-WCMQ_FD15506586/2020	EPI_ISL_1714034
hCoV-19/Qatar/QA-WCMQ_FD15506592/2020	EPI_ISL_1714040
hCoV-19/Qatar/QA-WCMQ_FD15506595/2020	EPI_ISL_1714043
hCoV-19/Qatar/QA-WCMQ_FD15506596/2020	EPI_ISL_1714044
hCoV-19/Qatar/QA-WCMQ_FD15540699/2020	EPI_ISL_1714056
hCoV-19/Qatar/QA-WCMQ_FD15540702/2020	EPI_ISL_1714058
hCoV-19/Qatar/QA-WCMQ_FD15540713/2020	EPI_ISL_1714060
hCoV-19/Qatar/QA-WCMQ_FD15540714/2020	EPI_ISL_1714061
hCoV-19/Qatar/QA-WCMQ_FD15540715/2020	EPI_ISL_1714062
hCoV-19/Qatar/QA-WCMQ_FD15540716/2020	EPI_ISL_1714063
hCoV-19/Qatar/QA-WCMQ_FD15617880/2020	EPI_ISL_1714078

hCoV-19/Qatar/QA-WCMQ_FD15617881/2020	EPI_ISL_1714079
hCoV-19/Qatar/QA-WCMQ_FD15617882/2020	EPI_ISL_1714080
hCoV-19/Qatar/QA-WCMQ_FD15617884/2020	EPI_ISL_1714081
hCoV-19/Qatar/QA-WCMQ_FD15617893/2020	EPI_ISL_1714082
hCoV-19/Qatar/QA-WCMQ_FD15617894/2020	EPI_ISL_1714083
hCoV-19/Qatar/QA-WCMQ_FD15617901/2020	EPI_ISL_1714084
hCoV-19/Qatar/QA-WCMQ_FD15617902/2020	EPI_ISL_1714085
hCoV-19/Qatar/QA-WCMQ_FD15617907/2020	EPI_ISL_1714086
hCoV-19/Qatar/QA-WCMQ_FD15617908/2020	EPI_ISL_1714087
hCoV-19/Qatar/QA-WCMQ_FD15617910/2020	EPI_ISL_1714089
hCoV-19/Qatar/QA-WCMQ_FD15617912/2020	EPI_ISL_1714090
hCoV-19/Qatar/QA-WCMQ_FD15617914/2020	EPI_ISL_1714091
hCoV-19/Qatar/QA-WCMQ_FD15617917/2020	EPI_ISL_1714092
hCoV-19/Qatar/QA-WCMQ_FD15617918/2020	EPI_ISL_1714093
hCoV-19/Qatar/QA-WCMQ_FD15617919/2020	EPI_ISL_1714094
hCoV-19/Qatar/QA-WCMQ_FD15617920/2020	EPI_ISL_1714095
hCoV-19/Qatar/QA-WCMQ_FD15617922/2020	EPI_ISL_1714096
hCoV-19/Qatar/QA-WCMQ_FD15617930/2020	EPI_ISL_1714100
hCoV-19/Qatar/QA-WCMQ_FD15617934/2020	EPI_ISL_1714102
hCoV-19/Qatar/QA-WCMQ_FD15617949/2020	EPI_ISL_1714107
hCoV-19/Qatar/QA-WCMQ_FD15617952/2020	EPI_ISL_1714108
hCoV-19/Qatar/QA-WCMQ_FD15617954/2020	EPI_ISL_1714110
hCoV-19/Qatar/QA-WCMQ_FD15617956/2020	EPI_ISL_1714112

hCoV-19/Qatar/QA-WCMQ_FD15617957/2020	EPI_ISL_1714113
hCoV-19/Qatar/QA-WCMQ_FD15617958/2020	EPI_ISL_1714114
hCoV-19/Qatar/QA-WCMQ_FD15617959/2020	EPI_ISL_1714115
hCoV-19/Qatar/QA-WCMQ_FD15617960/2020	EPI_ISL_1714116
hCoV-19/Qatar/QA-WCMQ_FD15617963/2020	EPI_ISL_1714117
hCoV-19/Qatar/QA-WCMQ_FD15617964/2020	EPI_ISL_1714118
hCoV-19/Qatar/QA-WCMQ_FD15617969/2020	EPI_ISL_1714122
hCoV-19/Qatar/QA-WCMQ_FD18272707/2020	EPI_ISL_1714696
hCoV-19/Qatar/QA-WCMQ_FD18272708/2020	EPI_ISL_1714697
hCoV-19/Qatar/QA-WCMQ_FD18272709/2020	EPI_ISL_1714698
hCoV-19/Qatar/QA-WCMQ_FD18272710/2020	EPI_ISL_1714699
hCoV-19/Qatar/QA-WCMQ_FD18272711/2020	EPI_ISL_1714700
hCoV-19/Qatar/QA-WCMQ_FD18272712/2020	EPI_ISL_1714701
hCoV-19/Qatar/QA-WCMQ_FD18272713/2020	EPI_ISL_1714702
hCoV-19/Qatar/QA-WCMQ_FD18272768/2020	EPI_ISL_1714740
hCoV-19/Qatar/QA-QU_05-J8/2020	EPI_ISL_1712895
hCoV-19/Qatar/QA-WCMQ_FD15506508/2020	EPI_ISL_1713956
hCoV-19/Qatar/QA-WCMQ_FD15506509/2020	EPI_ISL_1713957
hCoV-19/Qatar/QA-WCMQ_FD15506519/2020	EPI_ISL_1713967
hCoV-19/Qatar/QA-WCMQ_FD15506535/2020	EPI_ISL_1713983
hCoV-19/Qatar/QA-WCMQ_FD15506538/2020	EPI_ISL_1713986
hCoV-19/Qatar/QA-WCMQ_FD15506539/2020	EPI_ISL_1713987
hCoV-19/Qatar/QA-WCMQ_FD15506551/2020	EPI_ISL_1713999
hCoV-19/Qatar/QA-WCMQ_FD15506563/2020	EPI_ISL_1714011

hCoV-19/Qatar/QA-WCMQ_FD15506564/2020	EPI_ISL_1714012
hCoV-19/Qatar/QA-WCMQ_FD15506569/2020	EPI_ISL_1714017
hCoV-19/Qatar/QA-WCMQ_FD15506570/2020	EPI_ISL_1714018
hCoV-19/Qatar/QA-WCMQ_FD15506571/2020	EPI_ISL_1714019
hCoV-19/Qatar/QA-WCMQ_FD15506594/2020	EPI_ISL_1714042
hCoV-19/Qatar/QA-WCMQ_FD15540717/2020	EPI_ISL_1714064
hCoV-19/Qatar/QA-WCMQ_FD18365058/2020	EPI_ISL_1714749
hCoV-19/Qatar/QA-QU_09-1-F2/2020	EPI_ISL_1713103
hCoV-19/Qatar/QA-QU_09-1-A5/2020	EPI_ISL_1713076
hCoV-19/Qatar/QA-QU_09-1-F11/2020	EPI_ISL_1713101
hCoV-19/Qatar/QA-QU_09-1-H5/2020	EPI_ISL_1713118
hCoV-19/Qatar/QA-QU_09-2-C3/2020	EPI_ISL_1713126
hCoV-19/Qatar/QA-WCMQ_FD15506517/2020	EPI_ISL_1713965
hCoV-19/Qatar/QA-WCMQ_FD15506556/2020	EPI_ISL_1714004
hCoV-19/Qatar/QA-WCMQ_FD15506565/2020	EPI_ISL_1714013
hCoV-19/Qatar/QA-WCMQ_FD15506568/2020	EPI_ISL_1714016
hCoV-19/Qatar/QA-QU_09-1-A12/2020	EPI_ISL_1713073
hCoV-19/Qatar/QA-QU_09-2-C2/2020	EPI_ISL_1713125
hCoV-19/Qatar/QA-WCMQ_FD15506554/2020	EPI_ISL_1714002
hCoV-19/Qatar/QA-WCMQ_FD15506555/2020	EPI_ISL_1714003
hCoV-19/Qatar/QA-WCMQ_FD15506557/2020	EPI_ISL_1714005
hCoV-19/Qatar/QA-QU_04-B9/2020	EPI_ISL_1712803
hCoV-19/Qatar/QA-QU_04-H10/2020	EPI_ISL_1712823
hCoV-19/Qatar/QA-WCMQ_FD15540722/2020	EPI_ISL_1714066
hCoV-19/Qatar/QA-WCMQ_FD18160893/2020	EPI_ISL_1714359
hCoV-19/Qatar/QA-WCMQ_FD18193845/2020	EPI_ISL_1714573
hCoV-19/Qatar/QA-QU_04-D10/2020	EPI_ISL_1712805

hCoV-19/Qatar/QA-WCMQ_FD15540727/2020	EPI_ISL_1714068
hCoV-19/Qatar/QA-WCMQ_FD15651304/2020	EPI_ISL_1714133
hCoV-19/Qatar/QA-WCMQ_FD15651341/2020	EPI_ISL_1714137
hCoV-19/Qatar/QA-WCMQ_FD17438193/2020	EPI_ISL_1714186
hCoV-19/Qatar/QA-WCMQ_FD18193844/2020	EPI_ISL_1714572
hCoV-19/Qatar/QA-WCMQ_FD18272696/2020	EPI_ISL_1714689
hCoV-19/Qatar/QA-QU_09-2-E9/2020	EPI_ISL_1713134
hCoV-19/Qatar/QA-WCMQ_FD18160892/2020	EPI_ISL_1714358
hCoV-19/Qatar/QA-WCMQ_FD18160894/2020	EPI_ISL_1714360
hCoV-19/Qatar/QA-WCMQ_FD18193908/2020	EPI_ISL_1714635
hCoV-19/Qatar/QA-QU_09-1-D12/2020	EPI_ISL_1713091
hCoV-19/Qatar/QA-QU_09-2-C1/2020	EPI_ISL_1713124
hCoV-19/Qatar/QA-WCMQ_FD15651515/2020	EPI_ISL_1714138
hCoV-19/Qatar/QA-WCMQ_FD18160912/2020	EPI_ISL_1714363
hCoV-19/Qatar/QA-WCMQ_FD18160916/2020	EPI_ISL_1714364
hCoV-19/Qatar/QA-WCMQ_FD18193884/2020	EPI_ISL_1714611
hCoV-19/Qatar/QA-QU_05-E4/2020	EPI_ISL_1712856
hCoV-19/Qatar/QA-WCMQ_FD18193849/2020	EPI_ISL_1714577
hCoV-19/Qatar/QA-WCMQ_FD18193870/2020	EPI_ISL_1714597
hCoV-19/Qatar/QA-WCMQ_FD18193872/2020	EPI_ISL_1714599
hCoV-19/Qatar/QA-WCMQ_FD18193873/2020	EPI_ISL_1714600
hCoV-19/Qatar/QA-WCMQ_FD18193883/2020	EPI_ISL_1714610
hCoV-19/Qatar/QA-QU_03-H11/2020	EPI_ISL_1712781
hCoV-19/Qatar/QA-QU_03-H7/2020	EPI_ISL_1712785
hCoV-19/Qatar/QA-QU_04-A4/2020	EPI_ISL_1712794
hCoV-19/Qatar/QA-WCMQ_FD18193835/2020	EPI_ISL_1714563

hCoV-19/Qatar/QA-WCMQ_FD18193867/2020	EPI_ISL_1714594
hCoV-19/Qatar/QA-WCMQ_FD18270856/2020	EPI_ISL_1714671
hCoV-19/Qatar/QA-WCMQ_FD18270878/2020	EPI_ISL_1714672
hCoV-19/Qatar/QA-WCMQ_FD18270880/2020	EPI_ISL_1714674
hCoV-19/Qatar/QA-WCMQ_FD18365049/2020	EPI_ISL_1714747
hCoV-19/Qatar/QA-WCMQ_FD18365050/2020	EPI_ISL_1714748
hCoV-19/Qatar/QA-QU_03-B9/2020	EPI_ISL_1712747
hCoV-19/Qatar/QA-QU_03-D9/2020	EPI_ISL_1712761
hCoV-19/Qatar/QA-QU_03-E9/2020	EPI_ISL_1712767
hCoV-19/Qatar/QA-QU_03-F9/2020	EPI_ISL_1712773
hCoV-19/Qatar/QA-QU_03-S7/2020	EPI_ISL_1712791
hCoV-19/Qatar/QA-QU_03-S8/2020	EPI_ISL_1712792
hCoV-19/Qatar/QA-QU_09-2-F1/2020	EPI_ISL_1713135
hCoV-19/Qatar/QA-WCMQ_FD18193907/2020	EPI_ISL_1714634
hCoV-19/Qatar/QA-WCMQ_FD18272749/2020	EPI_ISL_1714729
hCoV-19/Qatar/QA-WCMQ_FD18272750/2020	EPI_ISL_1714730
hCoV-19/Qatar/QA-WCMQ_FD18272752/2020	EPI_ISL_1714731
hCoV-19/Qatar/QA-WCMQ_FD18272755/2020	EPI_ISL_1714732
hCoV-19/Qatar/QA-WCMQ_FD18272756/2020	EPI_ISL_1714733
hCoV-19/Qatar/QA-WCMQ_FD18272757/2020	EPI_ISL_1714734
hCoV-19/Qatar/QA-WCMQ_FD18272760/2020	EPI_ISL_1714735
hCoV-19/Qatar/QA-QU_03-A1/2020	EPI_ISL_1712729
hCoV-19/Qatar/QA-QU_03-A10/2020	EPI_ISL_1712730
hCoV-19/Qatar/QA-QU_03-A12/2020	EPI_ISL_1712732
hCoV-19/Qatar/QA-QU_03-A4/2020	EPI_ISL_1712734
hCoV-19/Qatar/QA-QU_03-A5/2020	EPI_ISL_1712735
hCoV-19/Qatar/QA-QU_03-A8/2020	EPI_ISL_1712737
hCoV-19/Qatar/QA-QU_03-A9/2020	EPI_ISL_1712738
hCoV-19/Qatar/QA-QU_03-B10/2020	EPI_ISL_1712739
hCoV-19/Qatar/QA-QU_03-B3/2020	EPI_ISL_1712743

hCoV-19/Qatar/QA-QU_03-B4/2020	EPI_ISL_1712744
hCoV-19/Qatar/QA-QU_03-B6/2020	EPI_ISL_1712745
hCoV-19/Qatar/QA-QU_03-B8/2020	EPI_ISL_1712746
hCoV-19/Qatar/QA-QU_03-C1/2020	EPI_ISL_1712748
hCoV-19/Qatar/QA-QU_03-C3/2020	EPI_ISL_1712749
hCoV-19/Qatar/QA-QU_03-C4/2020	EPI_ISL_1712750
hCoV-19/Qatar/QA-QU_03-C5/2020	EPI_ISL_1712751
hCoV-19/Qatar/QA-QU_03-C6/2020	EPI_ISL_1712752
hCoV-19/Qatar/QA-QU_03-C8/2020	EPI_ISL_1712753
hCoV-19/Qatar/QA-QU_03-D2/2020	EPI_ISL_1712754
hCoV-19/Qatar/QA-QU_03-D3/2020	EPI_ISL_1712755
hCoV-19/Qatar/QA-QU_03-D4/2020	EPI_ISL_1712756
hCoV-19/Qatar/QA-QU_03-D5/2020	EPI_ISL_1712757
hCoV-19/Qatar/QA-QU_03-D6/2020	EPI_ISL_1712758
hCoV-19/Qatar/QA-QU_03-D7/2020	EPI_ISL_1712759
hCoV-19/Qatar/QA-QU_03-D8/2020	EPI_ISL_1712760
hCoV-19/Qatar/QA-QU_03-E10/2020	EPI_ISL_1712762
hCoV-19/Qatar/QA-QU_03-E3/2020	EPI_ISL_1712763
hCoV-19/Qatar/QA-QU_03-E5/2020	EPI_ISL_1712764
hCoV-19/Qatar/QA-QU_03-E6/2020	EPI_ISL_1712765
hCoV-19/Qatar/QA-QU_03-E8/2020	EPI_ISL_1712766
hCoV-19/Qatar/QA-QU_03-F1/2020	EPI_ISL_1712768
hCoV-19/Qatar/QA-QU_03-F4/2020	EPI_ISL_1712771
hCoV-19/Qatar/QA-QU_03-F6/2020	EPI_ISL_1712772
hCoV-19/Qatar/QA-QU_03-G12/2020	EPI_ISL_1712775
hCoV-19/Qatar/QA-QU_03-G3/2020	EPI_ISL_1712776
hCoV-19/Qatar/QA-QU_03-G4/2020	EPI_ISL_1712777
hCoV-19/Qatar/QA-QU_03-G5/2020	EPI_ISL_1712778
hCoV-19/Qatar/QA-QU_03-G9/2020	EPI_ISL_1712779
hCoV-19/Qatar/QA-QU_03-H1/2020	EPI_ISL_1712780
hCoV-19/Qatar/QA-QU_03-H4/2020	EPI_ISL_1712784
hCoV-19/Qatar/QA-QU_03-S1/2020	EPI_ISL_1712787
hCoV-19/Qatar/QA-QU_03-S9/2020	EPI_ISL_1712793
hCoV-19/Qatar/QA-QU_09-2-E7/2020	EPI_ISL_1713133
hCoV-19/Qatar/QA-WCMQ_FD18272727/2020	EPI_ISL_1714712
hCoV-19/Qatar/QA-WCMQ_FD18272736/2020	EPI_ISL_1714720
hCoV-19/Qatar/QA-WCMQ_FD18272738/2020	EPI_ISL_1714721
hCoV-19/Qatar/QA-WCMQ_FD18272739/2020	EPI_ISL_1714722

hCoV-19/Qatar/QA-WCMQ_FD18272740/2020	EPI_ISL_1714723
hCoV-19/Qatar/QA-WCMQ_FD18272741/2020	EPI_ISL_1714724
hCoV-19/Qatar/QA-WCMQ_FD18272742/2020	EPI_ISL_1714725
hCoV-19/Qatar/QA-WCMQ_FD18272743/2020	EPI_ISL_1714726
hCoV-19/Qatar/QA-WCMQ_FD18272744/2020	EPI_ISL_1714727
hCoV-19/Qatar/QA-WCMQ_FD18272746/2020	EPI_ISL_1714728
hCoV-19/Qatar/QA-WCMQ_FD18272763/2020	EPI_ISL_1714736
hCoV-19/Qatar/QA-WCMQ_FD18272765/2020	EPI_ISL_1714737
hCoV-19/Qatar/QA-WCMQ_FD18272766/2020	EPI_ISL_1714738
hCoV-19/Qatar/QA-QU_03-A3/2020	EPI_ISL_1712733
hCoV-19/Qatar/QA-QU_03-A7/2020	EPI_ISL_1712736
hCoV-19/Qatar/QA-QU_03-B2/2020	EPI_ISL_1712742
hCoV-19/Qatar/QA-QU_03-F10/2020	EPI_ISL_1712769
hCoV-19/Qatar/QA-QU_03-F2/2020	EPI_ISL_1712770
hCoV-19/Qatar/QA-QU_03-G11/2020	EPI_ISL_1712774
hCoV-19/Qatar/QA-QU_03-H2/2020	EPI_ISL_1712783
hCoV-19/Qatar/QA-QU_03-S10/2020	EPI_ISL_1712788
hCoV-19/Qatar/QA-QU_03-S2/2020	EPI_ISL_1712789
hCoV-19/Qatar/QA-QU_03-S5/2020	EPI_ISL_1712790
hCoV-19/Qatar/QA-QU_09-2-E10/2020	EPI_ISL_1713129
hCoV-19/Qatar/QA-WCMQ_FD15481381/2020	EPI_ISL_1713949
hCoV-19/Qatar/QA-WCMQ_FD18193848/2020	EPI_ISL_1714576
hCoV-19/Qatar/QA-WCMQ_FD18272674/2020	EPI_ISL_1714675
hCoV-19/Qatar/QA-WCMQ_FD18272675/2020	EPI_ISL_1714676
hCoV-19/Qatar/QA-WCMQ_FD18272682/2020	EPI_ISL_1714677
hCoV-19/Qatar/QA-WCMQ_FD18272691/2020	EPI_ISL_1714684
hCoV-19/Qatar/QA-WCMQ_FD18272692/2020	EPI_ISL_1714685
hCoV-19/Qatar/QA-WCMQ_FD18272694/2020	EPI_ISL_1714687

hCoV-19/Qatar/QA-WCMQ_FD18272697/2020	EPI_ISL_1714690
hCoV-19/Qatar/QA-WCMQ_FD18272698/2020	EPI_ISL_1714691
hCoV-19/Qatar/QA-WCMQ_FD18272699/2020	EPI_ISL_1714692
hCoV-19/Qatar/QA-WCMQ_FD18272700/2020	EPI_ISL_1714693
hCoV-19/Qatar/QA-WCMQ_FD18272702/2020	EPI_ISL_1714694
hCoV-19/Qatar/QA-WCMQ_FD18272720/2020	EPI_ISL_1714706
hCoV-19/Qatar/QA-WCMQ_FD18272721/2020	EPI_ISL_1714707
hCoV-19/Qatar/QA-WCMQ_FD18272722/2020	EPI_ISL_1714708
hCoV-19/Qatar/QA-WCMQ_FD18272728/2020	EPI_ISL_1714713
hCoV-19/Qatar/QA-WCMQ_FD18272730/2020	EPI_ISL_1714714
hCoV-19/Qatar/QA-WCMQ_FD18272731/2020	EPI_ISL_1714715
hCoV-19/Qatar/QA-WCMQ_FD18272732/2020	EPI_ISL_1714716
hCoV-19/Qatar/QA-WCMQ_FD18272733/2020	EPI_ISL_1714717
hCoV-19/Qatar/QA-WCMQ_FD15535184/2020	EPI_ISL_1714049
hCoV-19/Qatar/QA-WCMQ_FD15381415/2020	EPI_ISL_1713947
hCoV-19/Qatar/QA-WCMQ_FD15382133/2020	EPI_ISL_1713948
hCoV-19/Qatar/QA-WCMQ_FD15540691/2020	EPI_ISL_1714053
hCoV-19/Qatar/QA-WCMQ_FD15540694/2020	EPI_ISL_1714054
hCoV-19/Qatar/QA-WCMQ_FD15540741/2020	EPI_ISL_1714076
hCoV-19/Qatar/QA-WCMQ_FD18254821/2020	EPI_ISL_1714669
hCoV-19/Qatar/QA-WCMQ_FD18254849/2020	EPI_ISL_1714670
hCoV-19/Qatar/QA-QU_05-G1/2020	EPI_ISL_1712865
hCoV-19/Qatar/QA-QU_05-I1/2020	EPI_ISL_1712881
hCoV-19/Qatar/QA-QU_05-J4/2020	EPI_ISL_1712891
hCoV-19/Qatar/QA-WCMQ_FD15540735/2020	EPI_ISL_1714074

hCoV-19/Qatar/QA-WCMQ_FD18215318/2020	EPI_ISL_1714658
hCoV-19/Qatar/QA-QU_04-B5/2020	EPI_ISL_1712801
hCoV-19/Qatar/QA-QU_04-B8/2020	EPI_ISL_1712802
hCoV-19/Qatar/QA-QU_05-A4/2020	EPI_ISL_1712833
hCoV-19/Qatar/QA-QU_05-E2/2020	EPI_ISL_1712854
hCoV-19/Qatar/QA-QU_05-F9/2020	EPI_ISL_1712864
hCoV-19/Qatar/QA-QU_05-G2/2020	EPI_ISL_1712867
hCoV-19/Qatar/QA-QU_05-H6/2020	EPI_ISL_1712878
hCoV-19/Qatar/QA-QU_05-H7/2020	EPI_ISL_1712879
hCoV-19/Qatar/QA-QU_05-I7/2020	EPI_ISL_1712887
hCoV-19/Qatar/QA-QU_05-J2/2020	EPI_ISL_1712889
hCoV-19/Qatar/QA-QU_05-J3/2020	EPI_ISL_1712890
hCoV-19/Qatar/QA-WCMQ_FD18216518/2020	EPI_ISL_1714663
hCoV-19/Qatar/QA-WCMQ_FD18222868/2020	EPI_ISL_1714666
hCoV-19/Qatar/QA-WCMQ_FD18222877/2020	EPI_ISL_1714667
hCoV-19/Qatar/QA-QU_05-B4/2020	EPI_ISL_1712837
hCoV-19/Qatar/QA-QU_05-C7/2020	EPI_ISL_1712846
hCoV-19/Qatar/QA-QU_05-D9/2020	EPI_ISL_1712851
hCoV-19/Qatar/QA-QU_05-E10/2020	EPI_ISL_1712853
hCoV-19/Qatar/QA-WCMQ_FD18216498/2020	EPI_ISL_1714662
hCoV-19/Qatar/QA-WCMQ_FD18216524/2020	EPI_ISL_1714664
hCoV-19/Qatar/QA-WCMQ_FD18364037/2020	EPI_ISL_1714743
hCoV-19/Qatar/QA-WCMQ_FD18366226/2020	EPI_ISL_1714755
hCoV-19/Qatar/QA-QU_05-B8/2020	EPI_ISL_1712841
hCoV-19/Qatar/QA-QU_05-F7/2020	EPI_ISL_1712862
hCoV-19/Qatar/QA-QU_05-F8/2020	EPI_ISL_1712863
hCoV-19/Qatar/QA-WCMQ_FD15674720/2020	EPI_ISL_1714156
hCoV-19/Qatar/QA-WCMQ_FD18364075/2020	EPI_ISL_1714744
hCoV-19/Qatar/QA-QU_04-E8/2020	EPI_ISL_1712813
hCoV-19/Qatar/QA-QU_05-B5/2020	EPI_ISL_1712838
hCoV-19/Qatar/QA-QU_05-B6/2020	EPI_ISL_1712839
hCoV-19/Qatar/QA-QU_05-B7/2020	EPI_ISL_1712840
hCoV-19/Qatar/QA-QU_05-E3/2020	EPI_ISL_1712855

hCoV-19/Qatar/QA-WCMQ_FD15650635/2020	EPI_ISL_1714132
hCoV-19/Qatar/QA-WCMQ_FD15651331/2020	EPI_ISL_1714135
hCoV-19/Qatar/QA-WCMQ_FD15651333/2020	EPI_ISL_1714136
hCoV-19/Qatar/QA-WCMQ_FD18215300/2020	EPI_ISL_1714653
hCoV-19/Qatar/QA-WCMQ_FD18215301/2020	EPI_ISL_1714654
hCoV-19/Qatar/QA-WCMQ_FD18215304/2020	EPI_ISL_1714655
hCoV-19/Qatar/QA-WCMQ_FD18215306/2020	EPI_ISL_1714656
hCoV-19/Qatar/QA-QU_04-A5/2020	EPI_ISL_1712795
hCoV-19/Qatar/QA-QU_04-A6/2020	EPI_ISL_1712796
hCoV-19/Qatar/QA-QU_04-J8/2020	EPI_ISL_1712830
hCoV-19/Qatar/QA-QU_05-G8/2020	EPI_ISL_1712872
hCoV-19/Qatar/QA-QU_05-I8/2020	EPI_ISL_1712888
hCoV-19/Qatar/QA-WCMQ_FD15651569/2020	EPI_ISL_1714139
hCoV-19/Qatar/QA-WCMQ_FD15671484/2020	EPI_ISL_1714143
hCoV-19/Qatar/QA-WCMQ_FD18193903/2020	EPI_ISL_1714630
hCoV-19/Qatar/QA-WCMQ_FD18215266/2020	EPI_ISL_1714650
hCoV-19/Qatar/QA-WCMQ_FD18215269/2020	EPI_ISL_1714651
hCoV-19/Qatar/QA-QU_05-A1/2020	EPI_ISL_1712831
hCoV-19/Qatar/QA-QU_05-C6/2020	EPI_ISL_1712845
hCoV-19/Qatar/QA-QU_05-D2/2020	EPI_ISL_1712849
hCoV-19/Qatar/QA-QU_05-D4/2020	EPI_ISL_1712850
hCoV-19/Qatar/QA-QU_05-E1/2020	EPI_ISL_1712852
hCoV-19/Qatar/QA-QU_05-G10/2020	EPI_ISL_1712866
hCoV-19/Qatar/QA-QU_05-G3/2020	EPI_ISL_1712868
hCoV-19/Qatar/QA-QU_05-G4/2020	EPI_ISL_1712869
hCoV-19/Qatar/QA-QU_05-G7/2020	EPI_ISL_1712871
hCoV-19/Qatar/QA-QU_05-H1/2020	EPI_ISL_1712873
hCoV-19/Qatar/QA-QU_05-H2/2020	EPI_ISL_1712875
hCoV-19/Qatar/QA-QU_05-H3/2020	EPI_ISL_1712876
hCoV-19/Qatar/QA-QU_05-H8/2020	EPI_ISL_1712880
hCoV-19/Qatar/QA-QU_05-I2/2020	EPI_ISL_1712882
hCoV-19/Qatar/QA-QU_05-I3/2020	EPI_ISL_1712883

hCoV-19/Qatar/QA-QU_05-J5/2020	EPI_ISL_1712892
hCoV-19/Qatar/QA-WCMQ_FD15648572/2020	EPI_ISL_1714126
hCoV-19/Qatar/QA-WCMQ_FD15653230/2020	EPI_ISL_1714141
hCoV-19/Qatar/QA-WCMQ_FD15653243/2020	EPI_ISL_1714142
hCoV-19/Qatar/QA-WCMQ_FD15672876/2020	EPI_ISL_1714145
hCoV-19/Qatar/QA-WCMQ_FD15672879/2020	EPI_ISL_1714146
hCoV-19/Qatar/QA-WCMQ_FD15672880/2020	EPI_ISL_1714147
hCoV-19/Qatar/QA-WCMQ_FD15672882/2020	EPI_ISL_1714148
hCoV-19/Qatar/QA-WCMQ_FD15672898/2020	EPI_ISL_1714150
hCoV-19/Qatar/QA-WCMQ_FD18193856/2020	EPI_ISL_1714584
hCoV-19/Qatar/QA-QU_04-A8/2020	EPI_ISL_1712797
hCoV-19/Qatar/QA-QU_04-A9/2020	EPI_ISL_1712798
hCoV-19/Qatar/QA-QU_04-E1/2020	EPI_ISL_1712809
hCoV-19/Qatar/QA-QU_04-E2/2020	EPI_ISL_1712811
hCoV-19/Qatar/QA-QU_04-E3/2020	EPI_ISL_1712812
hCoV-19/Qatar/QA-QU_04-E9/2020	EPI_ISL_1712814
hCoV-19/Qatar/QA-QU_04-H1/2020	EPI_ISL_1712822
hCoV-19/Qatar/QA-QU_04-H2/2020	EPI_ISL_1712824
hCoV-19/Qatar/QA-QU_04-H4/2020	EPI_ISL_1712825
hCoV-19/Qatar/QA-QU_04-H6/2020	EPI_ISL_1712826
hCoV-19/Qatar/QA-QU_04-H7/2020	EPI_ISL_1712827
hCoV-19/Qatar/QA-QU_04-H8/2020	EPI_ISL_1712828
hCoV-19/Qatar/QA-QU_04-I6/2020	EPI_ISL_1712829
hCoV-19/Qatar/QA-QU_05-B10/2020	EPI_ISL_1712835
hCoV-19/Qatar/QA-QU_05-B2/2020	EPI_ISL_1712836
hCoV-19/Qatar/QA-QU_05-C2/2020	EPI_ISL_1712843
hCoV-19/Qatar/QA-QU_05-C5/2020	EPI_ISL_1712844
hCoV-19/Qatar/QA-QU_05-E8/2020	EPI_ISL_1712859
hCoV-19/Qatar/QA-QU_05-F10/2020	EPI_ISL_1712860
hCoV-19/Qatar/QA-QU_05-H10/2020	EPI_ISL_1712874
hCoV-19/Qatar/QA-QU_05-H4/2020	EPI_ISL_1712877
hCoV-19/Qatar/QA-QU_05-I6/2020	EPI_ISL_1712886
hCoV-19/Qatar/QA-QU_05-J6/2020	EPI_ISL_1712893
hCoV-19/Qatar/QA-QU_05-J7/2020	EPI_ISL_1712894

hCoV-19/Qatar/QA-WCMQ_FD15645971/2020	EPI_ISL_1714125
hCoV-19/Qatar/QA-WCMQ_FD15648575/2020	EPI_ISL_1714127
hCoV-19/Qatar/QA-WCMQ_FD15649701/2020	EPI_ISL_1714128
hCoV-19/Qatar/QA-WCMQ_FD15649738/2020	EPI_ISL_1714130
hCoV-19/Qatar/QA-WCMQ_FD15672845/2020	EPI_ISL_1714144
hCoV-19/Qatar/QA-WCMQ_FD15672883/2020	EPI_ISL_1714149
hCoV-19/Qatar/QA-WCMQ_FD15673744/2020	EPI_ISL_1714152
hCoV-19/Qatar/QA-WCMQ_FD15674674/2020	EPI_ISL_1714153
hCoV-19/Qatar/QA-WCMQ_FD15674698/2020	EPI_ISL_1714154
hCoV-19/Qatar/QA-WCMQ_FD15674717/2020	EPI_ISL_1714155
hCoV-19/Qatar/QA-WCMQ_FD15674928/2020	EPI_ISL_1714157
hCoV-19/Qatar/QA-WCMQ_FD15674947/2020	EPI_ISL_1714158
hCoV-19/Qatar/QA-WCMQ_FD15674989/2020	EPI_ISL_1714159
hCoV-19/Qatar/QA-WCMQ_FD15676536/2020	EPI_ISL_1714161
hCoV-19/Qatar/QA-WCMQ_FD18193822/2020	EPI_ISL_1714550
hCoV-19/Qatar/QA-WCMQ_FD18193910/2020	EPI_ISL_1714637
hCoV-19/Qatar/QA-WCMQ_FD18214894/2020	EPI_ISL_1714645
hCoV-19/Qatar/QA-WCMQ_FD18214900/2020	EPI_ISL_1714646
hCoV-19/Qatar/QA-WCMQ_FD18214947/2020	EPI_ISL_1714647
hCoV-19/Qatar/QA-WCMQ_FD18215283/2020	EPI_ISL_1714652
hCoV-19/Qatar/QA-WCMQ_FD18215309/2020	EPI_ISL_1714657
hCoV-19/Qatar/QA-WCMQ_FD18215325/2020	EPI_ISL_1714659
hCoV-19/Qatar/QA-WCMQ_FD18215529/2020	EPI_ISL_1714661
hCoV-19/Qatar/QA-QU_04-D1/2020	EPI_ISL_1712804

hCoV-19/Qatar/QA-QU_04-D2/2020	EPI_ISL_1712806
hCoV-19/Qatar/QA-QU_04-D3/2020	EPI_ISL_1712807
hCoV-19/Qatar/QA-QU_04-D4/2020	EPI_ISL_1712808
hCoV-19/Qatar/QA-QU_04-E10/2020	EPI_ISL_1712810
hCoV-19/Qatar/QA-QU_04-F3/2020	EPI_ISL_1712815
hCoV-19/Qatar/QA-QU_04-F4/2020	EPI_ISL_1712816
hCoV-19/Qatar/QA-QU_04-F6/2020	EPI_ISL_1712817
hCoV-19/Qatar/QA-QU_04-F7/2020	EPI_ISL_1712818
hCoV-19/Qatar/QA-QU_04-F8/2020	EPI_ISL_1712819
hCoV-19/Qatar/QA-QU_04-G1/2020	EPI_ISL_1712820
hCoV-19/Qatar/QA-QU_04-G9/2020	EPI_ISL_1712821
hCoV-19/Qatar/QA-QU_05-C10/2020	EPI_ISL_1712842
hCoV-19/Qatar/QA-QU_05-C9/2020	EPI_ISL_1712848
hCoV-19/Qatar/QA-QU_05-I4/2020	EPI_ISL_1712884
hCoV-19/Qatar/QA-WCMQ_FD15649726/2020	EPI_ISL_1714129
hCoV-19/Qatar/QA-WCMQ_FD15650061/2020	EPI_ISL_1714131
hCoV-19/Qatar/QA-WCMQ_FD15673719/2020	EPI_ISL_1714151
hCoV-19/Qatar/QA-WCMQ_FD15675017/2020	EPI_ISL_1714160
hCoV-19/Qatar/QA-WCMQ_FD18193847/2020	EPI_ISL_1714575
hCoV-19/Qatar/QA-WCMQ_FD18193878/2020	EPI_ISL_1714605
hCoV-19/Qatar/QA-WCMQ_FD18193899/2020	EPI_ISL_1714626
hCoV-19/Qatar/QA-WCMQ_FD18193914/2020	EPI_ISL_1714641
hCoV-19/Qatar/QA-WCMQ_FD18215505/2020	EPI_ISL_1714660
hCoV-19/Qatar/QA-WCMQ_FD18363620/2020	EPI_ISL_1714741
hCoV-19/Qatar/QA-WCMQ_FD18363627/2020	EPI_ISL_1714742
hCoV-19/Qatar/QA-WCMQ_FD18365446/2020	EPI_ISL_1714750
hCoV-19/Qatar/QA-WCMQ_FD18365465/2020	EPI_ISL_1714751
hCoV-19/Qatar/QA-WCMQ_FD18365515/2020	EPI_ISL_1714752
hCoV-19/Qatar/QA-WCMQ_FD18365535/2020	EPI_ISL_1714753

hCoV-19/Qatar/QA-WCMQ_FD18365537/2020	EPI_ISL_1714754
hCoV-19/Qatar/QA-QU_05-C8/2020	EPI_ISL_1712847
hCoV-19/Qatar/QA-QU_05-E6/2020	EPI_ISL_1712857
hCoV-19/Qatar/QA-QU_05-E7/2020	EPI_ISL_1712858
hCoV-19/Qatar/QA-WCMQ_FD15652594/2020	EPI_ISL_1714140
hCoV-19/Qatar/QA-WCMQ_FD18193826/2020	EPI_ISL_1714554
hCoV-19/Qatar/QA-WCMQ_FD18193854/2020	EPI_ISL_1714582
hCoV-19/Qatar/QA-WCMQ_FD18193855/2020	EPI_ISL_1714583
hCoV-19/Qatar/QA-WCMQ_FD18193859/2020	EPI_ISL_1714586
hCoV-19/Qatar/QA-WCMQ_FD18193900/2020	EPI_ISL_1714627
hCoV-19/Qatar/QA-WCMQ_FD18193909/2020	EPI_ISL_1714636
hCoV-19/Qatar/QA-WCMQ_FD18193912/2020	EPI_ISL_1714639
hCoV-19/Qatar/QA-QU_05-A3/2020	EPI_ISL_1712832
hCoV-19/Qatar/QA-QU_05-B1/2020	EPI_ISL_1712834
hCoV-19/Qatar/QA-QU_05-F6/2020	EPI_ISL_1712861
hCoV-19/Qatar/QA-QU_05-G5/2020	EPI_ISL_1712870
hCoV-19/Qatar/QA-QU_05-I5/2020	EPI_ISL_1712885
hCoV-19/Qatar/QA-WCMQ_FD18160907/2020	EPI_ISL_1714362
hCoV-19/Qatar/QA-WCMQ_FD18193823/2020	EPI_ISL_1714551
hCoV-19/Qatar/QA-WCMQ_FD18193824/2020	EPI_ISL_1714552
hCoV-19/Qatar/QA-WCMQ_FD18193827/2020	EPI_ISL_1714555
hCoV-19/Qatar/QA-WCMQ_FD18193828/2020	EPI_ISL_1714556
hCoV-19/Qatar/QA-WCMQ_FD18193829/2020	EPI_ISL_1714557
hCoV-19/Qatar/QA-WCMQ_FD18193830/2020	EPI_ISL_1714558
hCoV-19/Qatar/QA-WCMQ_FD18193831/2020	EPI_ISL_1714559
hCoV-19/Qatar/QA-WCMQ_FD18193832/2020	EPI_ISL_1714560
hCoV-19/Qatar/QA-WCMQ_FD18193833/2020	EPI_ISL_1714561

hCoV-19/Qatar/QA-WCMQ_FD18193836/2020	EPI_ISL_1714564
hCoV-19/Qatar/QA-WCMQ_FD18193871/2020	EPI_ISL_1714598
hCoV-19/Qatar/QA-WCMQ_FD18193825/2020	EPI_ISL_1714553
hCoV-19/Qatar/QA-WCMQ_FD18193860/2020	EPI_ISL_1714587
hCoV-19/Qatar/QA-WCMQ_FD18193868/2020	EPI_ISL_1714595
hCoV-19/Qatar/QA-WCMQ_FD18193885/2020	EPI_ISL_1714612
hCoV-19/Qatar/QA-WCMQ_FD18193886/2020	EPI_ISL_1714613
hCoV-19/Qatar/QA-WCMQ_FD18193917/2020	EPI_ISL_1714644
hCoV-19/Qatar/QA-WCMQ_FD18193861/2020	EPI_ISL_1714588
hCoV-19/Qatar/QA-WCMQ_FD18193863/2020	EPI_ISL_1714590
hCoV-19/Qatar/QA-WCMQ_FD18193879/2020	EPI_ISL_1714606
hCoV-19/Qatar/QA-WCMQ_FD18193880/2020	EPI_ISL_1714607
hCoV-19/Qatar/QA-WCMQ_FD18193888/2020	EPI_ISL_1714615
hCoV-19/Qatar/QA-WCMQ_FD18193906/2020	EPI_ISL_1714633
hCoV-19/Qatar/QA-WCMQ_FD15540700/2020	EPI_ISL_1714057
hCoV-19/Qatar/QA-WCMQ_FD15540728/2020	EPI_ISL_1714069
hCoV-19/Qatar/QA-WCMQ_FD17059325/2020	EPI_ISL_1714178
hCoV-19/Qatar/QA-WCMQ_FD18193838/2020	EPI_ISL_1714566
hCoV-19/Qatar/QA-WCMQ_FD18193846/2020	EPI_ISL_1714574
hCoV-19/Qatar/QA-WCMQ_FD18193892/2020	EPI_ISL_1714619
hCoV-19/Qatar/QA-WCMQ_FD18193893/2020	EPI_ISL_1714620
hCoV-19/Qatar/QA-WCMQ_FD18193894/2020	EPI_ISL_1714621
hCoV-19/Qatar/QA-WCMQ_FD18193895/2020	EPI_ISL_1714622

hCoV-19/Qatar/QA-WCMQ_FD18193896/2020	EPI_ISL_1714623
hCoV-19/Qatar/QA-WCMQ_FD18193897/2020	EPI_ISL_1714624
hCoV-19/Qatar/QA-WCMQ_FD18193905/2020	EPI_ISL_1714632
hCoV-19/Qatar/QA-WCMQ_FD18193840/2020	EPI_ISL_1714568
hCoV-19/Qatar/QA-WCMQ_FD18193841/2020	EPI_ISL_1714569
hCoV-19/Qatar/QA-WCMQ_FD18193842/2020	EPI_ISL_1714570
hCoV-19/Qatar/QA-WCMQ_FD18193852/2020	EPI_ISL_1714580
hCoV-19/Qatar/QA-WCMQ_FD18193857/2020	EPI_ISL_1714585
hCoV-19/Qatar/QA-WCMQ_FD18193862/2020	EPI_ISL_1714589
hCoV-19/Qatar/QA-WCMQ_FD18193865/2020	EPI_ISL_1714592
hCoV-19/Qatar/QA-WCMQ_FD18193866/2020	EPI_ISL_1714593
hCoV-19/Qatar/QA-WCMQ_FD18193875/2020	EPI_ISL_1714602
hCoV-19/Qatar/QA-WCMQ_FD18193877/2020	EPI_ISL_1714604
hCoV-19/Qatar/QA-WCMQ_FD18193898/2020	EPI_ISL_1714625
hCoV-19/Qatar/QA-WCMQ_FD18193904/2020	EPI_ISL_1714631
hCoV-19/Qatar/QA-WCMQ_FD18193913/2020	EPI_ISL_1714640
hCoV-19/Qatar/QA-WCMQ_FD17059315/2020	EPI_ISL_1714172
hCoV-19/Qatar/QA-WCMQ_FD18193834/2020	EPI_ISL_1714562
hCoV-19/Qatar/QA-WCMQ_FD18193837/2020	EPI_ISL_1714565
hCoV-19/Qatar/QA-WCMQ_FD18193843/2020	EPI_ISL_1714571
hCoV-19/Qatar/QA-WCMQ_FD18193850/2020	EPI_ISL_1714578
hCoV-19/Qatar/QA-WCMQ_FD18193853/2020	EPI_ISL_1714581
hCoV-19/Qatar/QA-WCMQ_FD18193869/2020	EPI_ISL_1714596

hCoV-19/Qatar/QA-WCMQ_FD18193881/2020	EPI_ISL_1714608
hCoV-19/Qatar/QA-WCMQ_FD18193887/2020	EPI_ISL_1714614
hCoV-19/Qatar/QA-WCMQ_FD18193889/2020	EPI_ISL_1714616
hCoV-19/Qatar/QA-WCMQ_FD18193890/2020	EPI_ISL_1714617
hCoV-19/Qatar/QA-WCMQ_FD18193891/2020	EPI_ISL_1714618
hCoV-19/Qatar/QA-WCMQ_FD18193915/2020	EPI_ISL_1714642
hCoV-19/Qatar/QA-WCMQ_FD18193916/2020	EPI_ISL_1714643
hCoV-19/Qatar/QA-WCMQ_FD15540731/2020	EPI_ISL_1714071
hCoV-19/Qatar/QA-WCMQ_FD18160881/2020	EPI_ISL_1714355
hCoV-19/Qatar/QA-WCMQ_FD18193839/2020	EPI_ISL_1714567
hCoV-19/Qatar/QA-WCMQ_FD18193864/2020	EPI_ISL_1714591
hCoV-19/Qatar/QA-WCMQ_FD18193882/2020	EPI_ISL_1714609
hCoV-19/Qatar/QA-WCMQ_FD18193901/2020	EPI_ISL_1714628
hCoV-19/Qatar/QA-QU_07-1-C6/2020	EPI_ISL_1712924
hCoV-19/Qatar/QA-QU_07-1-D9/2020	EPI_ISL_1712939
hCoV-19/Qatar/QA-QU_07-1-F5/2020	EPI_ISL_1712949
hCoV-19/Qatar/QA-QU_07-2-A4/2020	EPI_ISL_1712980
hCoV-19/Qatar/QA-QU_07-2-B4/2020	EPI_ISL_1712989
hCoV-19/Qatar/QA-QU_07-2-C1/2020	EPI_ISL_1712995
hCoV-19/Qatar/QA-QU_07-2-C4/2020	EPI_ISL_1712999
hCoV-19/Qatar/QA-QU_07-2-D2/2020	EPI_ISL_1713005
hCoV-19/Qatar/QA-QU_07-2-D4/2020	EPI_ISL_1713007
hCoV-19/Qatar/QA-QU_07-2-E12/2020	EPI_ISL_1713014
hCoV-19/Qatar/QA-QU_07-2-F11/2020	EPI_ISL_1713022
hCoV-19/Qatar/QA-WCMQ_FD17462447/2020	EPI_ISL_1714266
hCoV-19/Qatar/QA-WCMQ_FD18160880/2020	EPI_ISL_1714354
hCoV-19/Qatar/QA-WCMQ_FD18160897/2020	EPI_ISL_1714361
hCoV-19/Qatar/QA-WCMQ_FD18193851/2020	EPI_ISL_1714579

hCoV-19/Qatar/QA-WCMQ_FD18193874/2020	EPI_ISL_1714601
hCoV-19/Qatar/QA-WCMQ_FD18193876/2020	EPI_ISL_1714603
hCoV-19/Qatar/QA-WCMQ_FD18193902/2020	EPI_ISL_1714629
hCoV-19/Qatar/QA-WCMQ_FD18193911/2020	EPI_ISL_1714638
hCoV-19/Qatar/QA-QU_07-1-D1/2020	EPI_ISL_1712928
hCoV-19/Qatar/QA-QU_07-1-E12/2020	EPI_ISL_1712943
hCoV-19/Qatar/QA-QU_07-1-F1/2020	EPI_ISL_1712946
hCoV-19/Qatar/QA-QU_07-1-H6/2020	EPI_ISL_1712972
hCoV-19/Qatar/QA-QU_07-2-A1/2020	EPI_ISL_1712975
hCoV-19/Qatar/QA-QU_07-2-B11/2020	EPI_ISL_1712985
hCoV-19/Qatar/QA-QU_07-2-B6/2020	EPI_ISL_1712991
hCoV-19/Qatar/QA-QU_07-2-C7/2020	EPI_ISL_1713001
hCoV-19/Qatar/QA-QU_07-2-D7/2020	EPI_ISL_1713009
hCoV-19/Qatar/QA-WCMQ_FD17059314/2020	EPI_ISL_1714171
hCoV-19/Qatar/QA-WCMQ_FD17462364/2020	EPI_ISL_1714190
hCoV-19/Qatar/QA-WCMQ_FD17462366/2020	EPI_ISL_1714192
hCoV-19/Qatar/QA-WCMQ_FD17462372/2020	EPI_ISL_1714197
hCoV-19/Qatar/QA-WCMQ_FD17462408/2020	EPI_ISL_1714230
hCoV-19/Qatar/QA-WCMQ_FD17462432/2020	EPI_ISL_1714251
hCoV-19/Qatar/QA-WCMQ_FD17462450/2020	EPI_ISL_1714269
hCoV-19/Qatar/QA-QU_07-1-B2/2020	EPI_ISL_1712910
hCoV-19/Qatar/QA-QU_07-1-B3/2020	EPI_ISL_1712911
hCoV-19/Qatar/QA-QU_07-1-B8/2020	EPI_ISL_1712916
hCoV-19/Qatar/QA-QU_07-1-C1/2020	EPI_ISL_1712918
hCoV-19/Qatar/QA-QU_07-1-D10/2020	EPI_ISL_1712929
hCoV-19/Qatar/QA-QU_07-2-A2/2020	EPI_ISL_1712978
hCoV-19/Qatar/QA-QU_07-2-D6/2020	EPI_ISL_1713008
hCoV-19/Qatar/QA-QU_07-2-E7/2020	EPI_ISL_1713019
hCoV-19/Qatar/QA-WCMQ_FD15540730/2020	EPI_ISL_1714070
hCoV-19/Qatar/QA-WCMQ_FD17462362/2020	EPI_ISL_1714188
hCoV-19/Qatar/QA-WCMQ_FD17462386/2020	EPI_ISL_1714211

hCoV-19/Qatar/QA-WCMQ_FD17462394/2020	EPI_ISL_1714217
hCoV-19/Qatar/QA-WCMQ_FD17462401/2020	EPI_ISL_1714223
hCoV-19/Qatar/QA-WCMQ_FD17462456/2020	EPI_ISL_1714274
hCoV-19/Qatar/QA-QU_07-1-F6/2020	EPI_ISL_1712950
hCoV-19/Qatar/QA-QU_07-2-A3/2020	EPI_ISL_1712979
hCoV-19/Qatar/QA-QU_07-2-B1/2020	EPI_ISL_1712983
hCoV-19/Qatar/QA-QU_07-2-B12/2020	EPI_ISL_1712986
hCoV-19/Qatar/QA-QU_07-2-B7/2020	EPI_ISL_1712992
hCoV-19/Qatar/QA-QU_07-2-E6/2020	EPI_ISL_1713018
hCoV-19/Qatar/QA-QU_07-2-F6/2020	EPI_ISL_1713028
hCoV-19/Qatar/QA-QU_07-2-G5/2020	EPI_ISL_1713032
hCoV-19/Qatar/QA-QU_07-2-G8/2020	EPI_ISL_1713034
hCoV-19/Qatar/QA-QU_07-2-H6/2020	EPI_ISL_1713038
hCoV-19/Qatar/QA-WCMQ_FD15540588/2020	EPI_ISL_1714050
hCoV-19/Qatar/QA-WCMQ_FD17059318/2020	EPI_ISL_1714175
hCoV-19/Qatar/QA-WCMQ_FD17462393/2020	EPI_ISL_1714216
hCoV-19/Qatar/QA-WCMQ_FD17462426/2020	EPI_ISL_1714247
hCoV-19/Qatar/QA-WCMQ_FD17462440/2020	EPI_ISL_1714259
hCoV-19/Qatar/QA-QU_07-1-B11/2020	EPI_ISL_1712908
hCoV-19/Qatar/QA-QU_07-1-D11/2020	EPI_ISL_1712930
hCoV-19/Qatar/QA-QU_07-1-H5/2020	EPI_ISL_1712971
hCoV-19/Qatar/QA-QU_07-2-C5/2020	EPI_ISL_1713000
hCoV-19/Qatar/QA-QU_07-2-D8/2020	EPI_ISL_1713010
hCoV-19/Qatar/QA-QU_07-2-F5/2020	EPI_ISL_1713027
hCoV-19/Qatar/QA-QU_07-2-G7/2020	EPI_ISL_1713033
hCoV-19/Qatar/QA-WCMQ_FD17462395/2020	EPI_ISL_1714218
hCoV-19/Qatar/QA-WCMQ_FD17462425/2020	EPI_ISL_1714246
hCoV-19/Qatar/QA-WCMQ_FD17462433/2020	EPI_ISL_1714252
hCoV-19/Qatar/QA-QU_07-1-A1/2020	EPI_ISL_1712896
hCoV-19/Qatar/QA-QU_07-1-A9/2020	EPI_ISL_1712906
hCoV-19/Qatar/QA-QU_07-1-B1/2020	EPI_ISL_1712907
hCoV-19/Qatar/QA-QU_07-1-C2/2020	EPI_ISL_1712921

hCoV-19/Qatar/QA-QU_07-1-D2/2020	EPI_ISL_1712932
hCoV-19/Qatar/QA-QU_07-1-D4/2020	EPI_ISL_1712934
hCoV-19/Qatar/QA-QU_07-1-G10/2020	EPI_ISL_1712955
hCoV-19/Qatar/QA-QU_07-1-G6/2020	EPI_ISL_1712962
hCoV-19/Qatar/QA-QU_07-2-C3/2020	EPI_ISL_1712998
hCoV-19/Qatar/QA-QU_07-2-E2/2020	EPI_ISL_1713015
hCoV-19/Qatar/QA-QU_07-2-E8/2020	EPI_ISL_1713020
hCoV-19/Qatar/QA-QU_07-2-F2/2020	EPI_ISL_1713024
hCoV-19/Qatar/QA-QU_07-2-F7/2020	EPI_ISL_1713029
hCoV-19/Qatar/QA-QU_08-A4/2020	EPI_ISL_1713042
hCoV-19/Qatar/QA-QU_08-A5/2020	EPI_ISL_1713043
hCoV-19/Qatar/QA-QU_08-C4/2020	EPI_ISL_1713051
hCoV-19/Qatar/QA-WCMQ_FD15540687/2020	EPI_ISL_1714051
hCoV-19/Qatar/QA-WCMQ_FD17059317/2020	EPI_ISL_1714174
hCoV-19/Qatar/QA-WCMQ_FD17462402/2020	EPI_ISL_1714224
hCoV-19/Qatar/QA-WCMQ_FD17462403/2020	EPI_ISL_1714225
hCoV-19/Qatar/QA-WCMQ_FD17462411/2020	EPI_ISL_1714233
hCoV-19/Qatar/QA-WCMQ_FD17462415/2020	EPI_ISL_1714236
hCoV-19/Qatar/QA-WCMQ_FD17462431/2020	EPI_ISL_1714250
hCoV-19/Qatar/QA-QU_07-1-D12/2020	EPI_ISL_1712931
hCoV-19/Qatar/QA-QU_07-1-D3/2020	EPI_ISL_1712933
hCoV-19/Qatar/QA-QU_07-2-A6/2020	EPI_ISL_1712981
hCoV-19/Qatar/QA-QU_07-2-B3/2020	EPI_ISL_1712988
hCoV-19/Qatar/QA-QU_07-2-D3/2020	EPI_ISL_1713006
hCoV-19/Qatar/QA-QU_07-2-H10/2020	EPI_ISL_1713035
hCoV-19/Qatar/QA-WCMQ_FD17462439/2020	EPI_ISL_1714258
hCoV-19/Qatar/QA-WCMQ_FD17462441/2020	EPI_ISL_1714260
hCoV-19/Qatar/QA-QU_07-1-A5/2020	EPI_ISL_1712902
hCoV-19/Qatar/QA-QU_07-1-C5/2020	EPI_ISL_1712923
hCoV-19/Qatar/QA-QU_07-1-D5/2020	EPI_ISL_1712935
hCoV-19/Qatar/QA-QU_07-1-D6/2020	EPI_ISL_1712936
hCoV-19/Qatar/QA-QU_07-1-E1/2020	EPI_ISL_1712940
hCoV-19/Qatar/QA-QU_07-1-E10/2020	EPI_ISL_1712941
hCoV-19/Qatar/QA-QU_07-1-H9/2020	EPI_ISL_1712974
hCoV-19/Qatar/QA-QU_07-2-B8/2020	EPI_ISL_1712993

hCoV-19/Qatar/QA-QU_07-2-E1/2020	EPI_ISL_1713012
hCoV-19/Qatar/QA-QU_07-2-F8/2020	EPI_ISL_1713030
hCoV-19/Qatar/QA-QU_08-C2/2020	EPI_ISL_1713049
hCoV-19/Qatar/QA-QU_08-C3/2020	EPI_ISL_1713050
hCoV-19/Qatar/QA-QU_08-D1/2020	EPI_ISL_1713053
hCoV-19/Qatar/QA-QU_08-G1/2020	EPI_ISL_1713064
hCoV-19/Qatar/QA-WCMQ_FD17462368/2020	EPI_ISL_1714194
hCoV-19/Qatar/QA-WCMQ_FD17462436/2020	EPI_ISL_1714255
hCoV-19/Qatar/QA-QU_07-1-A3/2020	EPI_ISL_1712900
hCoV-19/Qatar/QA-QU_07-1-A4/2020	EPI_ISL_1712901
hCoV-19/Qatar/QA-QU_07-1-A6/2020	EPI_ISL_1712903
hCoV-19/Qatar/QA-QU_07-1-B7/2020	EPI_ISL_1712915
hCoV-19/Qatar/QA-QU_07-1-C9/2020	EPI_ISL_1712927
hCoV-19/Qatar/QA-QU_07-1-F3/2020	EPI_ISL_1712947
hCoV-19/Qatar/QA-QU_07-1-G5/2020	EPI_ISL_1712961
hCoV-19/Qatar/QA-QU_07-1-H1/2020	EPI_ISL_1712965
hCoV-19/Qatar/QA-QU_07-2-B2/2020	EPI_ISL_1712987
hCoV-19/Qatar/QA-QU_08-B1/2020	EPI_ISL_1713044
hCoV-19/Qatar/QA-QU_08-B3/2020	EPI_ISL_1713046
hCoV-19/Qatar/QA-QU_08-B4/2020	EPI_ISL_1713047
hCoV-19/Qatar/QA-QU_08-C5/2020	EPI_ISL_1713052
hCoV-19/Qatar/QA-QU_08-D5/2020	EPI_ISL_1713054
hCoV-19/Qatar/QA-QU_08-E1/2020	EPI_ISL_1713055
hCoV-19/Qatar/QA-QU_08-E2/2020	EPI_ISL_1713056
hCoV-19/Qatar/QA-QU_08-F2/2020	EPI_ISL_1713060
hCoV-19/Qatar/QA-QU_08-F3/2020	EPI_ISL_1713061
hCoV-19/Qatar/QA-QU_08-F5/2020	EPI_ISL_1713063
hCoV-19/Qatar/QA-QU_08-G4/2020	EPI_ISL_1713067
hCoV-19/Qatar/QA-QU_08-G5/2020	EPI_ISL_1713068
hCoV-19/Qatar/QA-WCMQ_FD17059306/2020	EPI_ISL_1714163
hCoV-19/Qatar/QA-WCMQ_FD17462419/2020	EPI_ISL_1714240
hCoV-19/Qatar/QA-WCMQ_FD17462429/2020	EPI_ISL_1714249
hCoV-19/Qatar/QA-WCMQ_FD17462438/2020	EPI_ISL_1714257
hCoV-19/Qatar/QA-QU_07-1-B5/2020	EPI_ISL_1712913
hCoV-19/Qatar/QA-QU_07-1-B9/2020	EPI_ISL_1712917
hCoV-19/Qatar/QA-QU_07-2-B5/2020	EPI_ISL_1712990

hCoV-19/Qatar/QA-QU_08-F4/2020	EPI_ISL_1713062
hCoV-19/Qatar/QA-WCMQ_FD17059312/2020	EPI_ISL_1714169
hCoV-19/Qatar/QA-WCMQ_FD17462374/2020	EPI_ISL_1714199
hCoV-19/Qatar/QA-WCMQ_FD17462452/2020	EPI_ISL_1714271
hCoV-19/Qatar/QA-QU_07-1-D8/2020	EPI_ISL_1712938
hCoV-19/Qatar/QA-WCMQ_FD17462442/2020	EPI_ISL_1714261
hCoV-19/Qatar/QA-WCMQ_FD17462444/2020	EPI_ISL_1714263
hCoV-19/Qatar/QA-QU_07-1-A11/2020	EPI_ISL_1712897
hCoV-19/Qatar/QA-QU_07-1-G9/2020	EPI_ISL_1712964
hCoV-19/Qatar/QA-QU_07-2-C11/2020	EPI_ISL_1712996
hCoV-19/Qatar/QA-QU_08-G2/2020	EPI_ISL_1713065
hCoV-19/Qatar/QA-QU_08-H2/2020	EPI_ISL_1713069
hCoV-19/Qatar/QA-QU_08-H4/2020	EPI_ISL_1713071
hCoV-19/Qatar/QA-WCMQ_FD15540740/2020	EPI_ISL_1714075
hCoV-19/Qatar/QA-WCMQ_FD17462376/2020	EPI_ISL_1714201
hCoV-19/Qatar/QA-WCMQ_FD17462405/2020	EPI_ISL_1714227
hCoV-19/Qatar/QA-QU_07-1-D7/2020	EPI_ISL_1712937
hCoV-19/Qatar/QA-QU_07-1-G1/2020	EPI_ISL_1712954
hCoV-19/Qatar/QA-QU_08-A1/2020	EPI_ISL_1713040
hCoV-19/Qatar/QA-QU_08-B2/2020	EPI_ISL_1713045
hCoV-19/Qatar/QA-QU_08-E4/2020	EPI_ISL_1713057
hCoV-19/Qatar/QA-QU_08-G3/2020	EPI_ISL_1713066
hCoV-19/Qatar/QA-QU_08-H3/2020	EPI_ISL_1713070
hCoV-19/Qatar/QA-WCMQ_FD15540690/2020	EPI_ISL_1714052
hCoV-19/Qatar/QA-WCMQ_FD17462361/2020	EPI_ISL_1714187
hCoV-19/Qatar/QA-WCMQ_FD17462363/2020	EPI_ISL_1714189
hCoV-19/Qatar/QA-WCMQ_FD17462379/2020	EPI_ISL_1714204
hCoV-19/Qatar/QA-WCMQ_FD17462381/2020	EPI_ISL_1714206
hCoV-19/Qatar/QA-WCMQ_FD17462413/2020	EPI_ISL_1714234
hCoV-19/Qatar/QA-WCMQ_FD17462414/2020	EPI_ISL_1714235

hCoV-19/Qatar/QA-WCMQ_FD17462417/2020	EPI_ISL_1714238
hCoV-19/Qatar/QA-WCMQ_FD17462455/2020	EPI_ISL_1714273
hCoV-19/Qatar/QA-QU_07-1-A8/2020	EPI_ISL_1712905
hCoV-19/Qatar/QA-QU_07-1-B4/2020	EPI_ISL_1712912
hCoV-19/Qatar/QA-QU_07-1-E11/2020	EPI_ISL_1712942
hCoV-19/Qatar/QA-QU_07-1-E9/2020	EPI_ISL_1712945
hCoV-19/Qatar/QA-QU_07-1-F9/2020	EPI_ISL_1712953
hCoV-19/Qatar/QA-QU_07-1-H3/2020	EPI_ISL_1712969
hCoV-19/Qatar/QA-QU_07-1-H4/2020	EPI_ISL_1712970
hCoV-19/Qatar/QA-QU_07-1-H8/2020	EPI_ISL_1712973
hCoV-19/Qatar/QA-QU_07-2-F3/2020	EPI_ISL_1713025
hCoV-19/Qatar/QA-QU_08-B5/2020	EPI_ISL_1713048
hCoV-19/Qatar/QA-QU_08-E5/2020	EPI_ISL_1713058
hCoV-19/Qatar/QA-WCMQ_FD17462383/2020	EPI_ISL_1714208
hCoV-19/Qatar/QA-WCMQ_FD17462384/2020	EPI_ISL_1714209
hCoV-19/Qatar/QA-WCMQ_FD17462399/2020	EPI_ISL_1714221
hCoV-19/Qatar/QA-WCMQ_FD17462446/2020	EPI_ISL_1714265
hCoV-19/Qatar/QA-WCMQ_FD17462448/2020	EPI_ISL_1714267
hCoV-19/Qatar/QA-QU_07-1-F8/2020	EPI_ISL_1712952
hCoV-19/Qatar/QA-QU_07-2-C12/2020	EPI_ISL_1712997
hCoV-19/Qatar/QA-QU_07-2-F9/2020	EPI_ISL_1713031
hCoV-19/Qatar/QA-QU_07-2-H3/2020	EPI_ISL_1713036
hCoV-19/Qatar/QA-QU_07-2-H9/2020	EPI_ISL_1713039
hCoV-19/Qatar/QA-QU_08-F1/2020	EPI_ISL_1713059
hCoV-19/Qatar/QA-WCMQ_FD17462390/2020	EPI_ISL_1714214
hCoV-19/Qatar/QA-WCMQ_FD17462423/2020	EPI_ISL_1714244
hCoV-19/Qatar/QA-WCMQ_FD17462424/2020	EPI_ISL_1714245
hCoV-19/Qatar/QA-WCMQ_FD17462434/2020	EPI_ISL_1714253
hCoV-19/Qatar/QA-QU_07-1-A12/2020	EPI_ISL_1712898
hCoV-19/Qatar/QA-QU_07-1-C11/2020	EPI_ISL_1712920
hCoV-19/Qatar/QA-QU_07-1-E2/2020	EPI_ISL_1712944
hCoV-19/Qatar/QA-QU_07-1-H10/2020	EPI_ISL_1712966

hCoV-19/Qatar/QA-QU_07-1-H12/2020	EPI_ISL_1712967
hCoV-19/Qatar/QA-QU_07-2-A11/2020	EPI_ISL_1712977
hCoV-19/Qatar/QA-QU_09-1-C5/2020	EPI_ISL_1713087
hCoV-19/Qatar/QA-WCMQ_FD15540732/2020	EPI_ISL_1714072
hCoV-19/Qatar/QA-WCMQ_FD15540733/2020	EPI_ISL_1714073
hCoV-19/Qatar/QA-WCMQ_FD17462367/2020	EPI_ISL_1714193
hCoV-19/Qatar/QA-WCMQ_FD17462418/2020	EPI_ISL_1714239
hCoV-19/Qatar/QA-QU_07-1-G8/2020	EPI_ISL_1712963
hCoV-19/Qatar/QA-QU_07-2-B10/2020	EPI_ISL_1712984
hCoV-19/Qatar/QA-QU_07-2-D10/2020	EPI_ISL_1713002
hCoV-19/Qatar/QA-QU_09-2-E6/2020	EPI_ISL_1713132
hCoV-19/Qatar/QA-WCMQ_FD17462392/2020	EPI_ISL_1714215
hCoV-19/Qatar/QA-WCMQ_FD17462398/2020	EPI_ISL_1714220
hCoV-19/Qatar/QA-WCMQ_FD17462435/2020	EPI_ISL_1714254
hCoV-19/Qatar/QA-QU_07-1-A7/2020	EPI_ISL_1712904
hCoV-19/Qatar/QA-QU_07-1-B12/2020	EPI_ISL_1712909
hCoV-19/Qatar/QA-QU_07-1-C8/2020	EPI_ISL_1712926
hCoV-19/Qatar/QA-QU_07-1-G2/2020	EPI_ISL_1712958
hCoV-19/Qatar/QA-QU_07-1-G3/2020	EPI_ISL_1712959
hCoV-19/Qatar/QA-QU_07-1-H2/2020	EPI_ISL_1712968
hCoV-19/Qatar/QA-QU_07-2-A10/2020	EPI_ISL_1712976
hCoV-19/Qatar/QA-QU_07-2-B9/2020	EPI_ISL_1712994
hCoV-19/Qatar/QA-QU_07-2-D12/2020	EPI_ISL_1713004
hCoV-19/Qatar/QA-QU_07-2-E3/2020	EPI_ISL_1713016
hCoV-19/Qatar/QA-QU_07-2-E9/2020	EPI_ISL_1713021
hCoV-19/Qatar/QA-QU_08-A2/2020	EPI_ISL_1713041
hCoV-19/Qatar/QA-WCMQ_FD17462369/2020	EPI_ISL_1714195
hCoV-19/Qatar/QA-WCMQ_FD17462373/2020	EPI_ISL_1714198
hCoV-19/Qatar/QA-WCMQ_FD17462375/2020	EPI_ISL_1714200
hCoV-19/Qatar/QA-WCMQ_FD17462380/2020	EPI_ISL_1714205
hCoV-19/Qatar/QA-WCMQ_FD17462409/2020	EPI_ISL_1714231
hCoV-19/Qatar/QA-WCMQ_FD17462420/2020	EPI_ISL_1714241

hCoV-19/Qatar/QA-WCMQ_FD17462422/2020	EPI_ISL_1714243
hCoV-19/Qatar/QA-WCMQ_FD18160923/2020	EPI_ISL_1714365
hCoV-19/Qatar/QA-QU_07-1-G4/2020	EPI_ISL_1712960
hCoV-19/Qatar/QA-QU_07-2-E10/2020	EPI_ISL_1713013
hCoV-19/Qatar/QA-QU_07-2-F4/2020	EPI_ISL_1713026
hCoV-19/Qatar/QA-WCMQ_FD17462421/2020	EPI_ISL_1714242
hCoV-19/Qatar/QA-WCMQ_FD17462445/2020	EPI_ISL_1714264
hCoV-19/Qatar/QA-QU_07-1-B6/2020	EPI_ISL_1712914
hCoV-19/Qatar/QA-QU_07-1-C10/2020	EPI_ISL_1712919
hCoV-19/Qatar/QA-QU_07-1-F4/2020	EPI_ISL_1712948
hCoV-19/Qatar/QA-QU_07-1-G12/2020	EPI_ISL_1712957
hCoV-19/Qatar/QA-QU_07-2-A9/2020	EPI_ISL_1712982
hCoV-19/Qatar/QA-WCMQ_FD17462406/2020	EPI_ISL_1714228
hCoV-19/Qatar/QA-WCMQ_FD17462410/2020	EPI_ISL_1714232
hCoV-19/Qatar/QA-WCMQ_FD17462449/2020	EPI_ISL_1714268
hCoV-19/Qatar/QA-WCMQ_FD17462454/2020	EPI_ISL_1714272
hCoV-19/Qatar/QA-QU_07-2-D11/2020	EPI_ISL_1713003
hCoV-19/Qatar/QA-WCMQ_FD17462371/2020	EPI_ISL_1714196
hCoV-19/Qatar/QA-WCMQ_FD17462382/2020	EPI_ISL_1714207
hCoV-19/Qatar/QA-WCMQ_FD17462389/2020	EPI_ISL_1714213
hCoV-19/Qatar/QA-WCMQ_FD17462416/2020	EPI_ISL_1714237
hCoV-19/Qatar/QA-QU_07-1-A2/2020	EPI_ISL_1712899
hCoV-19/Qatar/QA-QU_07-1-C3/2020	EPI_ISL_1712922
hCoV-19/Qatar/QA-QU_07-1-F7/2020	EPI_ISL_1712951
hCoV-19/Qatar/QA-QU_07-2-D9/2020	EPI_ISL_1713011
hCoV-19/Qatar/QA-QU_07-2-F12/2020	EPI_ISL_1713023
hCoV-19/Qatar/QA-WCMQ_FD17462365/2020	EPI_ISL_1714191
hCoV-19/Qatar/QA-WCMQ_FD17462437/2020	EPI_ISL_1714256
hCoV-19/Qatar/QA-WCMQ_FD17462451/2020	EPI_ISL_1714270

hCoV-19/Qatar/QA-QU_07-1-C7/2020	EPI_ISL_1712925
hCoV-19/Qatar/QA-QU_07-1-G11/2020	EPI_ISL_1712956
hCoV-19/Qatar/QA-QU_07-2-E4/2020	EPI_ISL_1713017
hCoV-19/Qatar/QA-QU_07-2-H4/2020	EPI_ISL_1713037
hCoV-19/Qatar/QA-WCMQ_FD17059316/2020	EPI_ISL_1714173
hCoV-19/Qatar/QA-WCMQ_FD17462387/2020	EPI_ISL_1714212
hCoV-19/Qatar/QA-QU_09-1-C6/2020	EPI_ISL_1713088
hCoV-19/Qatar/QA-WCMQ_FD17462404/2020	EPI_ISL_1714226
hCoV-19/Qatar/QA-QU_12-6-E11/2020	EPI_ISL_1713292
hCoV-19/Qatar/QA-WCMQ_FD17462397/2020	EPI_ISL_1714219
hCoV-19/Qatar/QA-QU_12-6-D1/2020	EPI_ISL_1713290
hCoV-19/Qatar/QA-QU_12-6-E4/2020	EPI_ISL_1713294
hCoV-19/Qatar/QA-WCMQ_FD17059321/2020	EPI_ISL_1714177
hCoV-19/Qatar/QA-WCMQ_FD17462400/2020	EPI_ISL_1714222
hCoV-19/Qatar/QA-WCMQ_FD17462407/2020	EPI_ISL_1714229
hCoV-19/Qatar/QA-QU_12-6-B2/2020	EPI_ISL_1713289
hCoV-19/Qatar/QA-QU_12-6-E1/2020	EPI_ISL_1713291
hCoV-19/Qatar/QA-QU_12-6-E12/2020	EPI_ISL_1713293
hCoV-19/Qatar/QA-QU_12-6-G10/2020	EPI_ISL_1713295
hCoV-19/Qatar/QA-WCMQ_FD17059309/2020	EPI_ISL_1714166
hCoV-19/Qatar/QA-WCMQ_FD17462377/2020	EPI_ISL_1714202
hCoV-19/Qatar/QA-WCMQ_FD17462378/2020	EPI_ISL_1714203
hCoV-19/Qatar/QA-WCMQ_FD17462428/2020	EPI_ISL_1714248
hCoV-19/Qatar/QA-WCMQ_FD17462443/2020	EPI_ISL_1714262
hCoV-19/Qatar/QA-QU_12-7-F5/2020	EPI_ISL_1713316
hCoV-19/Qatar/QA-QU_12-7-G8/2020	EPI_ISL_1713320
hCoV-19/Qatar/QA-WCMQ_FD17462385/2020	EPI_ISL_1714210
hCoV-19/Qatar/QA-WCMQ_FD18189985/2020	EPI_ISL_1714464
hCoV-19/Qatar/QA-WCMQ_FD18189988/2020	EPI_ISL_1714467

hCoV-19/Qatar/QA-WCMQ_FD18189996/2020	EPI_ISL_1714475
hCoV-19/Qatar/QA-WCMQ_FD18190008/2020	EPI_ISL_1714487
hCoV-19/Qatar/QA-WCMQ_FD18190020/2020	EPI_ISL_1714498
hCoV-19/Qatar/QA-WCMQ_FD18190029/2020	EPI_ISL_1714507
hCoV-19/Qatar/QA-WCMQ_FD18190068/2020	EPI_ISL_1714542
hCoV-19/Qatar/QA-WCMQ_FD18190072/2020	EPI_ISL_1714546
hCoV-19/Qatar/QA-QU_12-7-C9/2020	EPI_ISL_1713305
hCoV-19/Qatar/QA-QU_12-7-E11/2020	EPI_ISL_1713311
hCoV-19/Qatar/QA-QU_12-7-E7/2020	EPI_ISL_1713314
hCoV-19/Qatar/QA-QU_12-7-H1/2020	EPI_ISL_1713321
hCoV-19/Qatar/QA-QU_12-7-H12/2020	EPI_ISL_1713322
hCoV-19/Qatar/QA-QU_12-7-H9/2020	EPI_ISL_1713326
hCoV-19/Qatar/QA-WCMQ_FD17059308/2020	EPI_ISL_1714165
hCoV-19/Qatar/QA-WCMQ_FD17059311/2020	EPI_ISL_1714168
hCoV-19/Qatar/QA-WCMQ_FD18190005/2020	EPI_ISL_1714484
hCoV-19/Qatar/QA-WCMQ_FD18190010/2020	EPI_ISL_1714489
hCoV-19/Qatar/QA-WCMQ_FD18190032/2020	EPI_ISL_1714510
hCoV-19/Qatar/QA-WCMQ_FD18190040/2020	EPI_ISL_1714518
hCoV-19/Qatar/QA-WCMQ_FD18190047/2020	EPI_ISL_1714523
hCoV-19/Qatar/QA-WCMQ_FD18190054/2020	EPI_ISL_1714529
hCoV-19/Qatar/QA-WCMQ_FD18190064/2020	EPI_ISL_1714538
hCoV-19/Qatar/QA-WCMQ_FD18190073/2020	EPI_ISL_1714547
hCoV-19/Qatar/QA-QU_12-7-A11/2020	EPI_ISL_1713297
hCoV-19/Qatar/QA-QU_12-7-D5/2020	EPI_ISL_1713307
hCoV-19/Qatar/QA-QU_12-7-H6/2020	EPI_ISL_1713324
hCoV-19/Qatar/QA-WCMQ_FD18189986/2020	EPI_ISL_1714465
hCoV-19/Qatar/QA-WCMQ_FD18189993/2020	EPI_ISL_1714472

hCoV-19/Qatar/QA-WCMQ_FD18190009/2020	EPI_ISL_1714488
hCoV-19/Qatar/QA-WCMQ_FD18190066/2020	EPI_ISL_1714540
hCoV-19/Qatar/QA-QU_12-7-A10/2020	EPI_ISL_1713296
hCoV-19/Qatar/QA-QU_12-7-B11/2020	EPI_ISL_1713301
hCoV-19/Qatar/QA-QU_12-7-D7/2020	EPI_ISL_1713308
hCoV-19/Qatar/QA-QU_12-7-E3/2020	EPI_ISL_1713312
hCoV-19/Qatar/QA-QU_12-7-F3/2020	EPI_ISL_1713315
hCoV-19/Qatar/QA-QU_12-7-H4/2020	EPI_ISL_1713323
hCoV-19/Qatar/QA-WCMQ_FD17059310/2020	EPI_ISL_1714167
hCoV-19/Qatar/QA-WCMQ_FD18189994/2020	EPI_ISL_1714473
hCoV-19/Qatar/QA-WCMQ_FD18189995/2020	EPI_ISL_1714474
hCoV-19/Qatar/QA-WCMQ_FD18190007/2020	EPI_ISL_1714486
hCoV-19/Qatar/QA-WCMQ_FD18190017/2020	EPI_ISL_1714495
hCoV-19/Qatar/QA-WCMQ_FD18190058/2020	EPI_ISL_1714533
hCoV-19/Qatar/QA-WCMQ_FD18190065/2020	EPI_ISL_1714539
hCoV-19/Qatar/QA-WCMQ_FD18190069/2020	EPI_ISL_1714543
hCoV-19/Qatar/QA-QU_12-7-A5/2020	EPI_ISL_1713299
hCoV-19/Qatar/QA-QU_12-7-C10/2020	EPI_ISL_1713304
hCoV-19/Qatar/QA-QU_12-7-H7/2020	EPI_ISL_1713325
hCoV-19/Qatar/QA-WCMQ_FD18189989/2020	EPI_ISL_1714468
hCoV-19/Qatar/QA-WCMQ_FD18190002/2020	EPI_ISL_1714481
hCoV-19/Qatar/QA-WCMQ_FD18190003/2020	EPI_ISL_1714482
hCoV-19/Qatar/QA-WCMQ_FD18190034/2020	EPI_ISL_1714512
hCoV-19/Qatar/QA-WCMQ_FD18190038/2020	EPI_ISL_1714516
hCoV-19/Qatar/QA-WCMQ_FD18190052/2020	EPI_ISL_1714527
hCoV-19/Qatar/QA-WCMQ_FD18190055/2020	EPI_ISL_1714530
hCoV-19/Qatar/QA-WCMQ_FD18190056/2020	EPI_ISL_1714531

hCoV-19/Qatar/QA-WCMQ_FD18190057/2020	EPI_ISL_1714532
hCoV-19/Qatar/QA-QU_12-7-B1/2020	EPI_ISL_1713300
hCoV-19/Qatar/QA-WCMQ_FD17059320/2020	EPI_ISL_1714176
hCoV-19/Qatar/QA-QU_12-7-E6/2020	EPI_ISL_1713313
hCoV-19/Qatar/QA-QU_12-7-G1/2020	EPI_ISL_1713318
hCoV-19/Qatar/QA-WCMQ_FD18189992/2020	EPI_ISL_1714471
hCoV-19/Qatar/QA-WCMQ_FD18190004/2020	EPI_ISL_1714483
hCoV-19/Qatar/QA-WCMQ_FD18190011/2020	EPI_ISL_1714490
hCoV-19/Qatar/QA-WCMQ_FD18190021/2020	EPI_ISL_1714499
hCoV-19/Qatar/QA-WCMQ_FD18190027/2020	EPI_ISL_1714505
hCoV-19/Qatar/QA-WCMQ_FD18190042/2020	EPI_ISL_1714519
hCoV-19/Qatar/QA-WCMQ_FD18190053/2020	EPI_ISL_1714528
hCoV-19/Qatar/QA-WCMQ_FD18190062/2020	EPI_ISL_1714536
hCoV-19/Qatar/QA-WCMQ_FD18190063/2020	EPI_ISL_1714537
hCoV-19/Qatar/QA-WCMQ_FD18190071/2020	EPI_ISL_1714545
hCoV-19/Qatar/QA-QU_12-7-D12/2020	EPI_ISL_1713306
hCoV-19/Qatar/QA-QU_12-7-G7/2020	EPI_ISL_1713319
hCoV-19/Qatar/QA-WCMQ_FD18189997/2020	EPI_ISL_1714476
hCoV-19/Qatar/QA-WCMQ_FD18190019/2020	EPI_ISL_1714497
hCoV-19/Qatar/QA-WCMQ_FD18190028/2020	EPI_ISL_1714506
hCoV-19/Qatar/QA-WCMQ_FD18190030/2020	EPI_ISL_1714508
hCoV-19/Qatar/QA-WCMQ_FD18190049/2020	EPI_ISL_1714524
hCoV-19/Qatar/QA-QU_12-7-B3/2020	EPI_ISL_1713302
hCoV-19/Qatar/QA-WCMQ_FD18189987/2020	EPI_ISL_1714466
hCoV-19/Qatar/QA-WCMQ_FD18189991/2020	EPI_ISL_1714470

hCoV-19/Qatar/QA-WCMQ_FD18190000/2020	EPI_ISL_1714479
hCoV-19/Qatar/QA-WCMQ_FD18190001/2020	EPI_ISL_1714480
hCoV-19/Qatar/QA-WCMQ_FD18190006/2020	EPI_ISL_1714485
hCoV-19/Qatar/QA-WCMQ_FD18190022/2020	EPI_ISL_1714500
hCoV-19/Qatar/QA-WCMQ_FD18190025/2020	EPI_ISL_1714503
hCoV-19/Qatar/QA-WCMQ_FD18190031/2020	EPI_ISL_1714509
hCoV-19/Qatar/QA-WCMQ_FD18190037/2020	EPI_ISL_1714515
hCoV-19/Qatar/QA-WCMQ_FD18190044/2020	EPI_ISL_1714520
hCoV-19/Qatar/QA-WCMQ_FD18190074/2020	EPI_ISL_1714548
hCoV-19/Qatar/QA-QU_12-7-D9/2020	EPI_ISL_1713310
hCoV-19/Qatar/QA-QU_12-7-F8/2020	EPI_ISL_1713317
hCoV-19/Qatar/QA-WCMQ_FD18189990/2020	EPI_ISL_1714469
hCoV-19/Qatar/QA-WCMQ_FD18189998/2020	EPI_ISL_1714477
hCoV-19/Qatar/QA-WCMQ_FD18189999/2020	EPI_ISL_1714478
hCoV-19/Qatar/QA-WCMQ_FD18190018/2020	EPI_ISL_1714496
hCoV-19/Qatar/QA-WCMQ_FD18190026/2020	EPI_ISL_1714504
hCoV-19/Qatar/QA-WCMQ_FD18190035/2020	EPI_ISL_1714513
hCoV-19/Qatar/QA-WCMQ_FD18190039/2020	EPI_ISL_1714517
hCoV-19/Qatar/QA-WCMQ_FD18190045/2020	EPI_ISL_1714521
hCoV-19/Qatar/QA-WCMQ_FD18190046/2020	EPI_ISL_1714522
hCoV-19/Qatar/QA-WCMQ_FD18190050/2020	EPI_ISL_1714525
hCoV-19/Qatar/QA-WCMQ_FD18190051/2020	EPI_ISL_1714526
hCoV-19/Qatar/QA-WCMQ_FD18190070/2020	EPI_ISL_1714544
hCoV-19/Qatar/QA-WCMQ_FD18190023/2020	EPI_ISL_1714501

hCoV-19/Qatar/QA-WCMQ_FD18190033/2020	EPI_ISL_1714511
hCoV-19/Qatar/QA-WCMQ_FD18190036/2020	EPI_ISL_1714514
hCoV-19/Qatar/QA-WCMQ_FD18190024/2020	EPI_ISL_1714502
hCoV-19/Qatar/QA-WCMQ_FD18190060/2020	EPI_ISL_1714535
hCoV-19/Qatar/QA-QU_12-7-A12/2020	EPI_ISL_1713298
hCoV-19/Qatar/QA-WCMQ_FD17059313/2020	EPI_ISL_1714170
hCoV-19/Qatar/QA-WCMQ_FD18190075/2020	EPI_ISL_1714549
hCoV-19/Qatar/QA-WCMQ_FD18190067/2020	EPI_ISL_1714541
hCoV-19/Qatar/QA-WCMQ_FD18189981/2020	EPI_ISL_1714461
hCoV-19/Qatar/QA-WCMQ_FD18190013/2020	EPI_ISL_1714492
hCoV-19/Qatar/QA-WCMQ_FD18190012/2020	EPI_ISL_1714491
hCoV-19/Qatar/QA-WCMQ_FD18190016/2020	EPI_ISL_1714494
hCoV-19/Qatar/QA-WCMQ_FD18190059/2020	EPI_ISL_1714534
hCoV-19/Qatar/QA-QU_12-7-B8/2020	EPI_ISL_1713303
hCoV-19/Qatar/QA-WCMQ_FD18189980/2020	EPI_ISL_1714460
hCoV-19/Qatar/QA-WCMQ_FD18189983/2020	EPI_ISL_1714462
hCoV-19/Qatar/QA-WCMQ_FD18189984/2020	EPI_ISL_1714463
hCoV-19/Qatar/QA-WCMQ_FD18190014/2020	EPI_ISL_1714493
hCoV-19/Qatar/QA-QU_12-7-D8/2020	EPI_ISL_1713309
hCoV-19/Qatar/QA-QU_12-8-A2/2020	EPI_ISL_1713329
hCoV-19/Qatar/QA-WCMQ_FD18163117/2020	EPI_ISL_1714387
hCoV-19/Qatar/QA-WCMQ_FD18163158/2020	EPI_ISL_1714426
hCoV-19/Qatar/QA-WCMQ_FD18163188/2020	EPI_ISL_1714456
hCoV-19/Qatar/QA-WCMQ_FD17059329/2020	EPI_ISL_1714181
hCoV-19/Qatar/QA-WCMQ_FD18163112/2020	EPI_ISL_1714382

hCoV-19/Qatar/QA-WCMQ_FD18163113/2020	EPI_ISL_1714383
hCoV-19/Qatar/QA-WCMQ_FD18163114/2020	EPI_ISL_1714384
hCoV-19/Qatar/QA-WCMQ_FD18163115/2020	EPI_ISL_1714385
hCoV-19/Qatar/QA-WCMQ_FD18163116/2020	EPI_ISL_1714386
hCoV-19/Qatar/QA-WCMQ_FD18163145/2020	EPI_ISL_1714414
hCoV-19/Qatar/QA-WCMQ_FD18163168/2020	EPI_ISL_1714436
hCoV-19/Qatar/QA-WCMQ_FD18163171/2020	EPI_ISL_1714439
hCoV-19/Qatar/QA-QU_12-8-E10/2020	EPI_ISL_1713345
hCoV-19/Qatar/QA-QU_12-8-G1/2020	EPI_ISL_1713355
hCoV-19/Qatar/QA-WCMQ_FD18163173/2020	EPI_ISL_1714441
hCoV-19/Qatar/QA-WCMQ_FD18163184/2020	EPI_ISL_1714452
hCoV-19/Qatar/QA-QU_12-8-B4/2020	EPI_ISL_1713334
hCoV-19/Qatar/QA-QU_12-8-C11/2020	EPI_ISL_1713338
hCoV-19/Qatar/QA-WCMQ_FD18163122/2020	EPI_ISL_1714392
hCoV-19/Qatar/QA-QU_12-8-B9/2020	EPI_ISL_1713336
hCoV-19/Qatar/QA-QU_12-8-D10/2020	EPI_ISL_1713342
hCoV-19/Qatar/QA-QU_12-8-F9/2020	EPI_ISL_1713354
hCoV-19/Qatar/QA-WCMQ_FD18163162/2020	EPI_ISL_1714430
hCoV-19/Qatar/QA-QU_12-8-A12/2020	EPI_ISL_1713328
hCoV-19/Qatar/QA-QU_12-8-E6/2020	EPI_ISL_1713347
hCoV-19/Qatar/QA-WCMQ_FD18163119/2020	EPI_ISL_1714389
hCoV-19/Qatar/QA-WCMQ_FD18163143/2020	EPI_ISL_1714412
hCoV-19/Qatar/QA-WCMQ_FD18163147/2020	EPI_ISL_1714416
hCoV-19/Qatar/QA-WCMQ_FD18163150/2020	EPI_ISL_1714419
hCoV-19/Qatar/QA-WCMQ_FD18163190/2020	EPI_ISL_1714458
hCoV-19/Qatar/QA-QU_12-8-C8/2020	EPI_ISL_1713341
hCoV-19/Qatar/QA-QU_12-8-A4/2020	EPI_ISL_1713330
hCoV-19/Qatar/QA-QU_12-8-C10/2020	EPI_ISL_1713337
hCoV-19/Qatar/QA-QU_12-8-G11/2020	EPI_ISL_1713356

hCoV-19/Qatar/QA-WCMQ_FD18163106/2020	EPI_ISL_1714376
hCoV-19/Qatar/QA-WCMQ_FD18163109/2020	EPI_ISL_1714379
hCoV-19/Qatar/QA-WCMQ_FD18163159/2020	EPI_ISL_1714427
hCoV-19/Qatar/QA-WCMQ_FD18163187/2020	EPI_ISL_1714455
hCoV-19/Qatar/QA-QU_12-8-F10/2020	EPI_ISL_1713349
hCoV-19/Qatar/QA-WCMQ_FD18163105/2020	EPI_ISL_1714375
hCoV-19/Qatar/QA-WCMQ_FD18163124/2020	EPI_ISL_1714394
hCoV-19/Qatar/QA-WCMQ_FD18163139/2020	EPI_ISL_1714408
hCoV-19/Qatar/QA-WCMQ_FD18163140/2020	EPI_ISL_1714409
hCoV-19/Qatar/QA-WCMQ_FD18163153/2020	EPI_ISL_1714421
hCoV-19/Qatar/QA-WCMQ_FD18163156/2020	EPI_ISL_1714424
hCoV-19/Qatar/QA-WCMQ_FD18163172/2020	EPI_ISL_1714440
hCoV-19/Qatar/QA-QU_12-8-C7/2020	EPI_ISL_1713340
hCoV-19/Qatar/QA-QU_12-8-G6/2020	EPI_ISL_1713358
hCoV-19/Qatar/QA-WCMQ_FD15519042/2020	EPI_ISL_1714046
hCoV-19/Qatar/QA-WCMQ_FD18163104/2020	EPI_ISL_1714374
hCoV-19/Qatar/QA-WCMQ_FD18163110/2020	EPI_ISL_1714380
hCoV-19/Qatar/QA-WCMQ_FD18163121/2020	EPI_ISL_1714391
hCoV-19/Qatar/QA-WCMQ_FD18163126/2020	EPI_ISL_1714396
hCoV-19/Qatar/QA-WCMQ_FD18163128/2020	EPI_ISL_1714397
hCoV-19/Qatar/QA-WCMQ_FD18163155/2020	EPI_ISL_1714423
hCoV-19/Qatar/QA-WCMQ_FD18163163/2020	EPI_ISL_1714431
hCoV-19/Qatar/QA-WCMQ_FD18163166/2020	EPI_ISL_1714434
hCoV-19/Qatar/QA-WCMQ_FD18163167/2020	EPI_ISL_1714435

hCoV-19/Qatar/QA-WCMQ_FD18163185/2020	EPI_ISL_1714453
hCoV-19/Qatar/QA-WCMQ_FD18163186/2020	EPI_ISL_1714454
hCoV-19/Qatar/QA-QU_12-8-A7/2020	EPI_ISL_1713331
hCoV-19/Qatar/QA-QU_12-8-E9/2020	EPI_ISL_1713348
hCoV-19/Qatar/QA-QU_12-8-H2/2020	EPI_ISL_1713360
hCoV-19/Qatar/QA-WCMQ_FD18163123/2020	EPI_ISL_1714393
hCoV-19/Qatar/QA-WCMQ_FD18163125/2020	EPI_ISL_1714395
hCoV-19/Qatar/QA-WCMQ_FD18163131/2020	EPI_ISL_1714400
hCoV-19/Qatar/QA-WCMQ_FD18163136/2020	EPI_ISL_1714405
hCoV-19/Qatar/QA-WCMQ_FD18163157/2020	EPI_ISL_1714425
hCoV-19/Qatar/QA-WCMQ_FD18163160/2020	EPI_ISL_1714428
hCoV-19/Qatar/QA-WCMQ_FD18163174/2020	EPI_ISL_1714442
hCoV-19/Qatar/QA-WCMQ_FD18163182/2020	EPI_ISL_1714450
hCoV-19/Qatar/QA-QU_12-8-D5/2020	EPI_ISL_1713343
hCoV-19/Qatar/QA-WCMQ_FD18163107/2020	EPI_ISL_1714377
hCoV-19/Qatar/QA-WCMQ_FD18163135/2020	EPI_ISL_1714404
hCoV-19/Qatar/QA-WCMQ_FD18163178/2020	EPI_ISL_1714446
hCoV-19/Qatar/QA-QU_12-8-A9/2020	EPI_ISL_1713332
hCoV-19/Qatar/QA-QU_12-8-C3/2020	EPI_ISL_1713339
hCoV-19/Qatar/QA-WCMQ_FD18163144/2020	EPI_ISL_1714413
hCoV-19/Qatar/QA-WCMQ_FD18163148/2020	EPI_ISL_1714417
hCoV-19/Qatar/QA-WCMQ_FD18163154/2020	EPI_ISL_1714422
hCoV-19/Qatar/QA-WCMQ_FD18163170/2020	EPI_ISL_1714438
hCoV-19/Qatar/QA-WCMQ_FD18163176/2020	EPI_ISL_1714444
hCoV-19/Qatar/QA-WCMQ_FD18163177/2020	EPI_ISL_1714445
hCoV-19/Qatar/QA-WCMQ_FD18163180/2020	EPI_ISL_1714448

hCoV-19/Qatar/QA-QU_12-8-F12/2020	EPI_ISL_1713350
hCoV-19/Qatar/QA-QU_12-8-H10/2020	EPI_ISL_1713359
hCoV-19/Qatar/QA-WCMQ_FD18163108/2020	EPI_ISL_1714378
hCoV-19/Qatar/QA-WCMQ_FD18163137/2020	EPI_ISL_1714406
hCoV-19/Qatar/QA-WCMQ_FD18163149/2020	EPI_ISL_1714418
hCoV-19/Qatar/QA-WCMQ_FD18163169/2020	EPI_ISL_1714437
hCoV-19/Qatar/QA-WCMQ_FD18163179/2020	EPI_ISL_1714447
hCoV-19/Qatar/QA-QU_12-8-F5/2020	EPI_ISL_1713351
hCoV-19/Qatar/QA-QU_13-C2/2020	EPI_ISL_1713371
hCoV-19/Qatar/QA-WCMQ_FD18163102/2020	EPI_ISL_1714372
hCoV-19/Qatar/QA-WCMQ_FD18163111/2020	EPI_ISL_1714381
hCoV-19/Qatar/QA-WCMQ_FD18163120/2020	EPI_ISL_1714390
hCoV-19/Qatar/QA-WCMQ_FD18163129/2020	EPI_ISL_1714398
hCoV-19/Qatar/QA-WCMQ_FD18163130/2020	EPI_ISL_1714399
hCoV-19/Qatar/QA-WCMQ_FD18163189/2020	EPI_ISL_1714457
hCoV-19/Qatar/QA-QU_12-8-B7/2020	EPI_ISL_1713335
hCoV-19/Qatar/QA-WCMQ_FD17059328/2020	EPI_ISL_1714180
hCoV-19/Qatar/QA-WCMQ_FD18163097/2020	EPI_ISL_1714367
hCoV-19/Qatar/QA-WCMQ_FD18163101/2020	EPI_ISL_1714371
hCoV-19/Qatar/QA-WCMQ_FD18163132/2020	EPI_ISL_1714401
hCoV-19/Qatar/QA-WCMQ_FD18163146/2020	EPI_ISL_1714415
hCoV-19/Qatar/QA-WCMQ_FD18163191/2020	EPI_ISL_1714459
hCoV-19/Qatar/QA-QU_12-8-D6/2020	EPI_ISL_1713344
hCoV-19/Qatar/QA-QU_12-8-F6/2020	EPI_ISL_1713352
hCoV-19/Qatar/QA-QU_13-B2/2020	EPI_ISL_1713367
hCoV-19/Qatar/QA-WCMQ_FD18163098/2020	EPI_ISL_1714368

hCoV-19/Qatar/QA-WCMQ_FD18163099/2020	EPI_ISL_1714369
hCoV-19/Qatar/QA-WCMQ_FD18163161/2020	EPI_ISL_1714429
hCoV-19/Qatar/QA-WCMQ_FD18163181/2020	EPI_ISL_1714449
hCoV-19/Qatar/QA-QU_12-8-A11/2020	EPI_ISL_1713327
hCoV-19/Qatar/QA-QU_12-8-G3/2020	EPI_ISL_1713357
hCoV-19/Qatar/QA-WCMQ_FD18163096/2020	EPI_ISL_1714366
hCoV-19/Qatar/QA-WCMQ_FD18163100/2020	EPI_ISL_1714370
hCoV-19/Qatar/QA-WCMQ_FD18163103/2020	EPI_ISL_1714373
hCoV-19/Qatar/QA-WCMQ_FD18163118/2020	EPI_ISL_1714388
hCoV-19/Qatar/QA-WCMQ_FD18163133/2020	EPI_ISL_1714402
hCoV-19/Qatar/QA-WCMQ_FD18163138/2020	EPI_ISL_1714407
hCoV-19/Qatar/QA-WCMQ_FD18163142/2020	EPI_ISL_1714411
hCoV-19/Qatar/QA-WCMQ_FD18163151/2020	EPI_ISL_1714420
hCoV-19/Qatar/QA-WCMQ_FD18163164/2020	EPI_ISL_1714432
hCoV-19/Qatar/QA-WCMQ_FD18163165/2020	EPI_ISL_1714433
hCoV-19/Qatar/QA-WCMQ_FD18163183/2020	EPI_ISL_1714451
hCoV-19/Qatar/QA-QU_12-8-E5/2020	EPI_ISL_1713346
hCoV-19/Qatar/QA-WCMQ_FD18163134/2020	EPI_ISL_1714403
hCoV-19/Qatar/QA-WCMQ_FD18163141/2020	EPI_ISL_1714410
hCoV-19/Qatar/QA-WCMQ_FD18163175/2020	EPI_ISL_1714443
hCoV-19/Qatar/QA-QU_12-8-B3/2020	EPI_ISL_1713333
hCoV-19/Qatar/QA-QU_12-8-F8/2020	EPI_ISL_1713353
hCoV-19/Qatar/QA-WCMQ_FD17059307/2020	EPI_ISL_1714164
hCoV-19/Qatar/QA-QU_12-8-H8/2020	EPI_ISL_1713361
hCoV-19/Qatar/QA-WCMQ_FD15542781/2020	EPI_ISL_1714077
hCoV-19/Qatar/QA-WCMQ_FD15692498/2020	EPI_ISL_1714162

hCoV-19/Qatar/QA-WCMQ_FD17059334/2020	EPI_ISL_1714184
hCoV-19/Qatar/QA-WCMQ_FD18231586/2020	EPI_ISL_1714668
hCoV-19/Qatar/QA-QU_10-1-A8/2020	EPI_ISL_1713145
hCoV-19/Qatar/QA-WCMQ_FD17059336/2020	EPI_ISL_1714185
hCoV-19/Qatar/QA-QU_10-2-F8/2020	EPI_ISL_1713178
hCoV-19/Qatar/QA-QU_10-2-A1/2020	EPI_ISL_1713146
hCoV-19/Qatar/QA-QU_10-2-B5/2020	EPI_ISL_1713154
hCoV-19/Qatar/QA-QU_10-2-C6/2020	EPI_ISL_1713157
hCoV-19/Qatar/QA-QU_10-2-F4/2020	EPI_ISL_1713174
hCoV-19/Qatar/QA-QU_10-2-F5/2020	EPI_ISL_1713175
hCoV-19/Qatar/QA-QU_10-2-F6/2020	EPI_ISL_1713176
hCoV-19/Qatar/QA-QU_10-2-G2/2020	EPI_ISL_1713180
hCoV-19/Qatar/QA-QU_10-2-H6/2020	EPI_ISL_1713192
hCoV-19/Qatar/QA-QU_10-1-A4/2020	EPI_ISL_1713141
hCoV-19/Qatar/QA-QU_10-1-A5/2020	EPI_ISL_1713142
hCoV-19/Qatar/QA-QU_10-1-A7/2020	EPI_ISL_1713144
hCoV-19/Qatar/QA-QU_10-2-A3/2020	EPI_ISL_1713147
hCoV-19/Qatar/QA-QU_10-2-A4/2020	EPI_ISL_1713148
hCoV-19/Qatar/QA-QU_10-2-A6/2020	EPI_ISL_1713149
hCoV-19/Qatar/QA-QU_10-2-A7/2020	EPI_ISL_1713150
hCoV-19/Qatar/QA-QU_10-2-A9/2020	EPI_ISL_1713151
hCoV-19/Qatar/QA-QU_10-2-B1/2020	EPI_ISL_1713152
hCoV-19/Qatar/QA-QU_10-2-B2/2020	EPI_ISL_1713153
hCoV-19/Qatar/QA-QU_10-2-B7/2020	EPI_ISL_1713155
hCoV-19/Qatar/QA-QU_10-2-C1/2020	EPI_ISL_1713156
hCoV-19/Qatar/QA-QU_10-2-C9/2020	EPI_ISL_1713158
hCoV-19/Qatar/QA-QU_10-2-D1/2020	EPI_ISL_1713159
hCoV-19/Qatar/QA-QU_10-2-D3/2020	EPI_ISL_1713160
hCoV-19/Qatar/QA-QU_10-2-D5/2020	EPI_ISL_1713161
hCoV-19/Qatar/QA-QU_10-2-D9/2020	EPI_ISL_1713164
hCoV-19/Qatar/QA-QU_10-2-E2/2020	EPI_ISL_1713165
hCoV-19/Qatar/QA-QU_10-2-E3/2020	EPI_ISL_1713166
hCoV-19/Qatar/QA-QU_10-2-E4/2020	EPI_ISL_1713167
hCoV-19/Qatar/QA-QU_10-2-E5/2020	EPI_ISL_1713168
hCoV-19/Qatar/QA-QU_10-2-E6/2020	EPI_ISL_1713169
hCoV-19/Qatar/QA-QU_10-2-E7/2020	EPI_ISL_1713170
hCoV-19/Qatar/QA-QU_10-2-E8/2020	EPI_ISL_1713171
hCoV-19/Qatar/QA-QU_10-2-F1/2020	EPI_ISL_1713172

hCoV-19/Qatar/QA-QU_10-2-F7/2020	EPI_ISL_1713177
hCoV-19/Qatar/QA-QU_10-2-G1/2020	EPI_ISL_1713179
hCoV-19/Qatar/QA-QU_10-2-G3/2020	EPI_ISL_1713181
hCoV-19/Qatar/QA-QU_10-2-G4/2020	EPI_ISL_1713182
hCoV-19/Qatar/QA-QU_10-2-G5/2020	EPI_ISL_1713183
hCoV-19/Qatar/QA-QU_10-2-G6/2020	EPI_ISL_1713184
hCoV-19/Qatar/QA-QU_10-2-G8/2020	EPI_ISL_1713186
hCoV-19/Qatar/QA-QU_10-2-H3/2020	EPI_ISL_1713189
hCoV-19/Qatar/QA-QU_10-2-H5/2020	EPI_ISL_1713191
hCoV-19/Qatar/QA-QU_10-1-A1/2020	EPI_ISL_1713139
hCoV-19/Qatar/QA-QU_10-2-D6/2020	EPI_ISL_1713162
hCoV-19/Qatar/QA-QU_10-2-D7/2020	EPI_ISL_1713163
hCoV-19/Qatar/QA-QU_10-2-F2/2020	EPI_ISL_1713173
hCoV-19/Qatar/QA-QU_10-2-H1/2020	EPI_ISL_1713187
hCoV-19/Qatar/QA-QU_10-2-H2/2020	EPI_ISL_1713188
hCoV-19/Qatar/QA-QU_10-2-H8/2020	EPI_ISL_1713193
hCoV-19/Qatar/QA-QU_10-1-A6/2020	EPI_ISL_1713143
hCoV-19/Qatar/QA-QU_10-2-H4/2020	EPI_ISL_1713190
hCoV-19/Qatar/QA-QU_10-1-A2/2020	EPI_ISL_1713140
hCoV-19/Qatar/QA-WCMQ_FD17059326/2020	EPI_ISL_1714179
hCoV-19/Qatar/QA-QU_10-2-G7/2020	EPI_ISL_1713185
hCoV-19/Qatar/QA-WCMQ_FD17059332/2020	EPI_ISL_1714183
hCoV-19/Qatar/QA-QU_17-B1/2020	EPI_ISL_1713473
hCoV-19/Qatar/QA-QU_17-A6/2020	EPI_ISL_1713471
hCoV-19/Qatar/QA-QU_17-B6/2020	EPI_ISL_1713475
hCoV-19/Qatar/QA-QU_17-B7/2020	EPI_ISL_1713476
hCoV-19/Qatar/QA-QU_17-B10/2020	EPI_ISL_1713474
hCoV-19/Qatar/QA-QU_17-A3/2020	EPI_ISL_1713469
hCoV-19/Qatar/QA-QU_17-A7/2020	EPI_ISL_1713472
hCoV-19/Qatar/QA-QU_17-C1/2020	EPI_ISL_1713477
hCoV-19/Qatar/QA-QU_17-A2/2020	EPI_ISL_1713468
hCoV-19/Qatar/QA-QU_17-C4/2020	EPI_ISL_1713478
hCoV-19/Qatar/QA-QU_17-C5/2020	EPI_ISL_1713479
hCoV-19/Qatar/QA-QU_13-A2/2020	EPI_ISL_1713363
hCoV-19/Qatar/QA-QU_13-A3/2020	EPI_ISL_1713364
hCoV-19/Qatar/QA-QU_13-H1/2020	EPI_ISL_1713377
hCoV-19/Qatar/QA-QU_17-C6/2020	EPI_ISL_1713480
hCoV-19/Qatar/QA-QU_17-C7/2020	EPI_ISL_1713481
hCoV-19/Qatar/QA-QU_17-C8/2020	EPI_ISL_1713482
hCoV-19/Qatar/QA-QU_17-C9/2020	EPI_ISL_1713483

hCoV-19/Qatar/QA-QU_13-D1/2020	EPI_ISL_1713373
hCoV-19/Qatar/QA-QU_13-G1/2020	EPI_ISL_1713376
hCoV-19/Qatar/QA-QU_17-A4/2020	EPI_ISL_1713470
hCoV-19/Qatar/QA-WCMQ_FD17059331/2020	EPI_ISL_1714182
hCoV-19/Qatar/QA-QU_13-C1/2020	EPI_ISL_1713370
hCoV-19/Qatar/QA-QU_13-A1/2020	EPI_ISL_1713362
hCoV-19/Qatar/QA-QU_13-B1/2020	EPI_ISL_1713366
hCoV-19/Qatar/QA-QU_12-10-A8/2020	EPI_ISL_1713201
hCoV-19/Qatar/QA-QU_12-10-B4/2020	EPI_ISL_1713205
hCoV-19/Qatar/QA-QU_12-10-B10/2020	EPI_ISL_1713203
hCoV-19/Qatar/QA-QU_12-10-H10/2020	EPI_ISL_1713233
hCoV-19/Qatar/QA-QU_12-10-H6/2020	EPI_ISL_1713236
hCoV-19/Qatar/QA-QU_12-10-D8/2020	EPI_ISL_1713219
hCoV-19/Qatar/QA-QU_12-10-A2/2020	EPI_ISL_1713197
hCoV-19/Qatar/QA-QU_12-10-A3/2020	EPI_ISL_1713198
hCoV-19/Qatar/QA-QU_12-10-B1/2020	EPI_ISL_1713202
hCoV-19/Qatar/QA-QU_12-10-B2/2020	EPI_ISL_1713204
hCoV-19/Qatar/QA-QU_12-10-B6/2020	EPI_ISL_1713206
hCoV-19/Qatar/QA-QU_12-10-C2/2020	EPI_ISL_1713208
hCoV-19/Qatar/QA-QU_12-10-D1/2020	EPI_ISL_1713213
hCoV-19/Qatar/QA-QU_12-10-D6/2020	EPI_ISL_1713217
hCoV-19/Qatar/QA-QU_12-10-F2/2020	EPI_ISL_1713227
hCoV-19/Qatar/QA-QU_12-10-A7/2020	EPI_ISL_1713200
hCoV-19/Qatar/QA-QU_12-10-C6/2020	EPI_ISL_1713210
hCoV-19/Qatar/QA-QU_12-10-F6/2020	EPI_ISL_1713229
hCoV-19/Qatar/QA-QU_12-10-D12/2020	EPI_ISL_1713216
hCoV-19/Qatar/QA-QU_12-10-F10/2020	EPI_ISL_1713225
hCoV-19/Qatar/QA-QU_12-11-E2/2020	EPI_ISL_1713263
hCoV-19/Qatar/QA-QU_12-11-H2/2020	EPI_ISL_1713281
hCoV-19/Qatar/QA-QU_12-10-E3/2020	EPI_ISL_1713222
hCoV-19/Qatar/QA-QU_12-10-H7/2020	EPI_ISL_1713237
hCoV-19/Qatar/QA-QU_12-10-C3/2020	EPI_ISL_1713209
hCoV-19/Qatar/QA-QU_12-10-C8/2020	EPI_ISL_1713211
hCoV-19/Qatar/QA-QU_12-10-E9/2020	EPI_ISL_1713224
hCoV-19/Qatar/QA-QU_12-10-A6/2020	EPI_ISL_1713199
hCoV-19/Qatar/QA-QU_12-10-F4/2020	EPI_ISL_1713228
hCoV-19/Qatar/QA-QU_12-10-F8/2020	EPI_ISL_1713230
hCoV-19/Qatar/QA-QU_12-10-G12/2020	EPI_ISL_1713232
hCoV-19/Qatar/QA-QU_12-10-H3/2020	EPI_ISL_1713235
hCoV-19/Qatar/QA-QU_12-10-H8/2020	EPI_ISL_1713238

hCoV-19/Qatar/QA-QU_12-10-C9/2020	EPI_ISL_1713212
hCoV-19/Qatar/QA-QU_12-10-G10/2020	EPI_ISL_1713231
hCoV-19/Qatar/QA-QU_12-10-A10/2020	EPI_ISL_1713194
hCoV-19/Qatar/QA-QU_12-10-H11/2020	EPI_ISL_1713234
hCoV-19/Qatar/QA-QU_12-10-D9/2020	EPI_ISL_1713220
hCoV-19/Qatar/QA-QU_12-10-E7/2020	EPI_ISL_1713223
hCoV-19/Qatar/QA-QU_12-10-B8/2020	EPI_ISL_1713207
hCoV-19/Qatar/QA-QU_12-10-F11/2020	EPI_ISL_1713226
hCoV-19/Qatar/QA-QU_12-10-A11/2020	EPI_ISL_1713195
hCoV-19/Qatar/QA-QU_12-10-A12/2020	EPI_ISL_1713196
hCoV-19/Qatar/QA-QU_12-10-D10/2020	EPI_ISL_1713214
hCoV-19/Qatar/QA-QU_12-10-D11/2020	EPI_ISL_1713215
hCoV-19/Qatar/QA-QU_12-10-D7/2020	EPI_ISL_1713218
hCoV-19/Qatar/QA-QU_12-10-E10/2020	EPI_ISL_1713221
hCoV-19/Qatar/QA-QU_12-11-H11/2020	EPI_ISL_1713279
hCoV-19/Qatar/QA-QU_13-B3/2020	EPI_ISL_1713368
hCoV-19/Qatar/QA-QU_12-11-E11/2020	EPI_ISL_1713261
hCoV-19/Qatar/QA-QU_12-11-E9/2020	EPI_ISL_1713267
hCoV-19/Qatar/QA-QU_12-11-B3/2020	EPI_ISL_1713250
hCoV-19/Qatar/QA-QU_12-11-F11/2020	EPI_ISL_1713268
hCoV-19/Qatar/QA-QU_12-11-G1/2020	EPI_ISL_1713275
hCoV-19/Qatar/QA-QU_12-11-G10/2020	EPI_ISL_1713276
hCoV-19/Qatar/QA-QU_12-11-B2/2020	EPI_ISL_1713249
hCoV-19/Qatar/QA-QU_12-11-F9/2020	EPI_ISL_1713274
hCoV-19/Qatar/QA-QU_12-11-E12/2020	EPI_ISL_1713262
hCoV-19/Qatar/QA-QU_12-11-H10/2020	EPI_ISL_1713278
hCoV-19/Qatar/QA-QU_12-11-C9/2020	EPI_ISL_1713256
hCoV-19/Qatar/QA-QU_12-11-D3/2020	EPI_ISL_1713258
hCoV-19/Qatar/QA-QU_12-11-A12/2020	EPI_ISL_1713240
hCoV-19/Qatar/QA-QU_12-11-B4/2020	EPI_ISL_1713251
hCoV-19/Qatar/QA-QU_12-11-A2/2020	EPI_ISL_1713241
hCoV-19/Qatar/QA-QU_12-11-B10/2020	EPI_ISL_1713247
hCoV-19/Qatar/QA-QU_12-11-D1/2020	EPI_ISL_1713257
hCoV-19/Qatar/QA-QU_12-11-A10/2020	EPI_ISL_1713239
hCoV-19/Qatar/QA-QU_12-11-D4/2020	EPI_ISL_1713259
hCoV-19/Qatar/QA-QU_12-11-E3/2020	EPI_ISL_1713264
hCoV-19/Qatar/QA-QU_13-D2/2020	EPI_ISL_1713374
hCoV-19/Qatar/QA-QU_12-11-B1/2020	EPI_ISL_1713246
hCoV-19/Qatar/QA-QU_12-11-F12/2020	EPI_ISL_1713269
hCoV-19/Qatar/QA-QU_12-11-B12/2020	EPI_ISL_1713248
hCoV-19/Qatar/QA-QU_12-11-F8/2020	EPI_ISL_1713273

hCoV-19/Qatar/QA-QU_12-11-H9/2020	EPI_ISL_1713285
hCoV-19/Qatar/QA-QU_12-11-F3/2020	EPI_ISL_1713270
hCoV-19/Qatar/QA-QU_12-11-H12/2020	EPI_ISL_1713280
hCoV-19/Qatar/QA-QU_12-11-H3/2020	EPI_ISL_1713282
hCoV-19/Qatar/QA-QU_12-11-A5/2020	EPI_ISL_1713242
hCoV-19/Qatar/QA-QU_12-11-B5/2020	EPI_ISL_1713252
hCoV-19/Qatar/QA-QU_12-11-B9/2020	EPI_ISL_1713254
hCoV-19/Qatar/QA-QU_12-11-C6/2020	EPI_ISL_1713255
hCoV-19/Qatar/QA-QU_12-11-H4/2020	EPI_ISL_1713283
hCoV-19/Qatar/QA-QU_12-11-H7/2020	EPI_ISL_1713284
hCoV-19/Qatar/QA-QU_13-C3/2020	EPI_ISL_1713372
hCoV-19/Qatar/QA-QU_12-11-A6/2020	EPI_ISL_1713243
hCoV-19/Qatar/QA-QU_12-11-D8/2020	EPI_ISL_1713260
hCoV-19/Qatar/QA-QU_12-11-G6/2020	EPI_ISL_1713277
hCoV-19/Qatar/QA-QU_12-11-A7/2020	EPI_ISL_1713244
hCoV-19/Qatar/QA-QU_12-11-B6/2020	EPI_ISL_1713253
hCoV-19/Qatar/QA-QU_12-11-E8/2020	EPI_ISL_1713266
hCoV-19/Qatar/QA-QU_12-11-F5/2020	EPI_ISL_1713271
hCoV-19/Qatar/QA-QU_12-11-F6/2020	EPI_ISL_1713272
hCoV-19/Qatar/QA-QU_12-11-A8/2020	EPI_ISL_1713245
hCoV-19/Qatar/QA-QU_12-11-E7/2020	EPI_ISL_1713265
hCoV-19/Qatar/QA-QU_13-A4/2020	EPI_ISL_1713365
hCoV-19/Qatar/QA-QU_13-B4/2020	EPI_ISL_1713369
hCoV-19/Qatar/QA-QU_13-E3/2020	EPI_ISL_1713375
hCoV-19/Qatar/QA-QU_15-1-B4/2020	EPI_ISL_1713382
hCoV-19/Qatar/QA-QU_15-1-F3/2020	EPI_ISL_1713386
hCoV-19/Qatar/QA-QU_15-1-A8/2020	EPI_ISL_1713381
hCoV-19/Qatar/QA-QU_15-1-C8/2020	EPI_ISL_1713383
hCoV-19/Qatar/QA-QU_15-1-E8/2020	EPI_ISL_1713385
hCoV-19/Qatar/QA-QU_15-1-G8/2020	EPI_ISL_1713388
hCoV-19/Qatar/QA-QU_15-1-H7/2020	EPI_ISL_1713389
hCoV-19/Qatar/QA-QU_15-1-E7/2020	EPI_ISL_1713384
hCoV-19/Qatar/QA-QU_15-1-G12/2020	EPI_ISL_1713387
hCoV-19/Qatar/QA-QU_15-5-F2/2020	EPI_ISL_1713417
hCoV-19/Qatar/QA-QU_17-H9/2020	EPI_ISL_1713517
hCoV-19/Qatar/QA-WCMQ_FD17571280/2020	EPI_ISL_1714279
hCoV-19/Qatar/QA-WCMQ_FD17571337/2020	EPI_ISL_1714328
hCoV-19/Qatar/QA-WCMQ_FD17571338/2020	EPI_ISL_1714329

hCoV-19/Qatar/QA-WCMQ_FD17571339/2020	EPI_ISL_1714330
hCoV-19/Qatar/QA-WCMQ_FD17571341/2020	EPI_ISL_1714332
hCoV-19/Qatar/QA-WCMQ_FD17571342/2020	EPI_ISL_1714333
hCoV-19/Qatar/QA-WCMQ_FD17571361/2020	EPI_ISL_1714351
hCoV-19/Qatar/QA-WCMQ_FD17571364/2020	EPI_ISL_1714352
hCoV-19/Qatar/QA-WCMQ_FD17571367/2020	EPI_ISL_1714353
hCoV-19/Qatar/QA-QU_15-4-D11/2020	EPI_ISL_1713412
hCoV-19/Qatar/QA-QU_15-4-F12/2020	EPI_ISL_1713413
hCoV-19/Qatar/QA-WCMQ_FD17571340/2020	EPI_ISL_1714331
hCoV-19/Qatar/QA-QU_15-2-G8/2020	EPI_ISL_1713398
hCoV-19/Qatar/QA-QU_15-2-H8/2020	EPI_ISL_1713402
hCoV-19/Qatar/QA-QU_15-2-F12/2020	EPI_ISL_1713397
hCoV-19/Qatar/QA-QU_14-C2/2020	EPI_ISL_1713378
hCoV-19/Qatar/QA-QU_15-2-A10/2020	EPI_ISL_1713390
hCoV-19/Qatar/QA-QU_15-2-C11/2020	EPI_ISL_1713392
hCoV-19/Qatar/QA-QU_15-2-H10/2020	EPI_ISL_1713399
hCoV-19/Qatar/QA-QU_15-2-H6/2020	EPI_ISL_1713401
hCoV-19/Qatar/QA-QU_14-C3/2020	EPI_ISL_1713379
hCoV-19/Qatar/QA-QU_14-C5/2020	EPI_ISL_1713380
hCoV-19/Qatar/QA-QU_15-2-A6/2020	EPI_ISL_1713391
hCoV-19/Qatar/QA-QU_15-2-C2/2020	EPI_ISL_1713393
hCoV-19/Qatar/QA-QU_15-2-H5/2020	EPI_ISL_1713400
hCoV-19/Qatar/QA-QU_17-D1/2020	EPI_ISL_1713484
hCoV-19/Qatar/QA-QU_15-4-F5/2020	EPI_ISL_1713414
hCoV-19/Qatar/QA-QU_17-D2/2020	EPI_ISL_1713487
hCoV-19/Qatar/QA-WCMQ_FD17571288/2020	EPI_ISL_1714285
hCoV-19/Qatar/QA-WCMQ_FD17571300/2020	EPI_ISL_1714297
hCoV-19/Qatar/QA-WCMQ_FD17571303/2020	EPI_ISL_1714300
hCoV-19/Qatar/QA-WCMQ_FD17571343/2020	EPI_ISL_1714334
hCoV-19/Qatar/QA-QU_15-4-C7/2020	EPI_ISL_1713411
hCoV-19/Qatar/QA-QU_15-4-F6/2020	EPI_ISL_1713415
hCoV-19/Qatar/QA-QU_15-5-A12/2020	EPI_ISL_1713416
hCoV-19/Qatar/QA-QU_16-E10/2020	EPI_ISL_1713456

hCoV-19/Qatar/QA-QU_16-E11/2020	EPI_ISL_1713457
hCoV-19/Qatar/QA-QU_16-E12/2020	EPI_ISL_1713458
hCoV-19/Qatar/QA-QU_16-E6/2020	EPI_ISL_1713459
hCoV-19/Qatar/QA-QU_16-E7/2020	EPI_ISL_1713460
hCoV-19/Qatar/QA-QU_16-E8/2020	EPI_ISL_1713461
hCoV-19/Qatar/QA-QU_16-E9/2020	EPI_ISL_1713462
hCoV-19/Qatar/QA-QU_16-F1/2020	EPI_ISL_1713463
hCoV-19/Qatar/QA-QU_16-F2/2020	EPI_ISL_1713466
hCoV-19/Qatar/QA-QU_16-F3/2020	EPI_ISL_1713467
hCoV-19/Qatar/QA-QU_17-D5/2020	EPI_ISL_1713488
hCoV-19/Qatar/QA-WCMQ_FD17571274/2020	EPI_ISL_1714275
hCoV-19/Qatar/QA-WCMQ_FD17571275/2020	EPI_ISL_1714276
hCoV-19/Qatar/QA-WCMQ_FD17571276/2020	EPI_ISL_1714277
hCoV-19/Qatar/QA-WCMQ_FD17571279/2020	EPI_ISL_1714278
hCoV-19/Qatar/QA-WCMQ_FD17571281/2020	EPI_ISL_1714280
hCoV-19/Qatar/QA-WCMQ_FD17571282/2020	EPI_ISL_1714281
hCoV-19/Qatar/QA-WCMQ_FD17571283/2020	EPI_ISL_1714282
hCoV-19/Qatar/QA-WCMQ_FD17571286/2020	EPI_ISL_1714283
hCoV-19/Qatar/QA-WCMQ_FD17571287/2020	EPI_ISL_1714284
hCoV-19/Qatar/QA-WCMQ_FD17571289/2020	EPI_ISL_1714286
hCoV-19/Qatar/QA-WCMQ_FD17571290/2020	EPI_ISL_1714287
hCoV-19/Qatar/QA-WCMQ_FD17571291/2020	EPI_ISL_1714288
hCoV-19/Qatar/QA-WCMQ_FD17571292/2020	EPI_ISL_1714289
hCoV-19/Qatar/QA-WCMQ_FD17571293/2020	EPI_ISL_1714290
hCoV-19/Qatar/QA-WCMQ_FD17571294/2020	EPI_ISL_1714291
hCoV-19/Qatar/QA-WCMQ_FD17571295/2020	EPI_ISL_1714292
hCoV-19/Qatar/QA-WCMQ_FD17571296/2020	EPI_ISL_1714293

hCoV-19/Qatar/QA-WCMQ_FD17571297/2020	EPI_ISL_1714294
hCoV-19/Qatar/QA-WCMQ_FD17571298/2020	EPI_ISL_1714295
hCoV-19/Qatar/QA-WCMQ_FD17571299/2020	EPI_ISL_1714296
hCoV-19/Qatar/QA-WCMQ_FD17571301/2020	EPI_ISL_1714298
hCoV-19/Qatar/QA-WCMQ_FD17571302/2020	EPI_ISL_1714299
hCoV-19/Qatar/QA-WCMQ_FD17571304/2020	EPI_ISL_1714301
hCoV-19/Qatar/QA-WCMQ_FD17571305/2020	EPI_ISL_1714302
hCoV-19/Qatar/QA-WCMQ_FD17571306/2020	EPI_ISL_1714303
hCoV-19/Qatar/QA-WCMQ_FD17571308/2020	EPI_ISL_1714304
hCoV-19/Qatar/QA-WCMQ_FD17571309/2020	EPI_ISL_1714305
hCoV-19/Qatar/QA-WCMQ_FD17571310/2020	EPI_ISL_1714306
hCoV-19/Qatar/QA-WCMQ_FD17571311/2020	EPI_ISL_1714307
hCoV-19/Qatar/QA-WCMQ_FD17571312/2020	EPI_ISL_1714308
hCoV-19/Qatar/QA-WCMQ_FD17571313/2020	EPI_ISL_1714309
hCoV-19/Qatar/QA-WCMQ_FD17571314/2020	EPI_ISL_1714310
hCoV-19/Qatar/QA-WCMQ_FD17571315/2020	EPI_ISL_1714311
hCoV-19/Qatar/QA-WCMQ_FD17571316/2020	EPI_ISL_1714312
hCoV-19/Qatar/QA-WCMQ_FD17571317/2020	EPI_ISL_1714313
hCoV-19/Qatar/QA-WCMQ_FD17571318/2020	EPI_ISL_1714314
hCoV-19/Qatar/QA-WCMQ_FD17571319/2020	EPI_ISL_1714315
hCoV-19/Qatar/QA-WCMQ_FD17571320/2020	EPI_ISL_1714316
hCoV-19/Qatar/QA-WCMQ_FD17571322/2020	EPI_ISL_1714317
hCoV-19/Qatar/QA-WCMQ_FD17571324/2020	EPI_ISL_1714318

hCoV-19/Qatar/QA-WCMQ_FD17571325/2020	EPI_ISL_1714319
hCoV-19/Qatar/QA-WCMQ_FD17571326/2020	EPI_ISL_1714320
hCoV-19/Qatar/QA-WCMQ_FD17571327/2020	EPI_ISL_1714321
hCoV-19/Qatar/QA-WCMQ_FD17571328/2020	EPI_ISL_1714322
hCoV-19/Qatar/QA-WCMQ_FD17571329/2020	EPI_ISL_1714323
hCoV-19/Qatar/QA-WCMQ_FD17571332/2020	EPI_ISL_1714324
hCoV-19/Qatar/QA-WCMQ_FD17571333/2020	EPI_ISL_1714325
hCoV-19/Qatar/QA-WCMQ_FD17571335/2020	EPI_ISL_1714326
hCoV-19/Qatar/QA-WCMQ_FD17571336/2020	EPI_ISL_1714327
hCoV-19/Qatar/QA-WCMQ_FD17571344/2020	EPI_ISL_1714335
hCoV-19/Qatar/QA-WCMQ_FD17571345/2020	EPI_ISL_1714336
hCoV-19/Qatar/QA-WCMQ_FD17571346/2020	EPI_ISL_1714337
hCoV-19/Qatar/QA-WCMQ_FD17571347/2020	EPI_ISL_1714338
hCoV-19/Qatar/QA-WCMQ_FD17571348/2020	EPI_ISL_1714339
hCoV-19/Qatar/QA-WCMQ_FD17571349/2020	EPI_ISL_1714340
hCoV-19/Qatar/QA-WCMQ_FD17571350/2020	EPI_ISL_1714341
hCoV-19/Qatar/QA-WCMQ_FD17571351/2020	EPI_ISL_1714342
hCoV-19/Qatar/QA-WCMQ_FD17571352/2020	EPI_ISL_1714343
hCoV-19/Qatar/QA-WCMQ_FD17571353/2020	EPI_ISL_1714344
hCoV-19/Qatar/QA-WCMQ_FD17571354/2020	EPI_ISL_1714345
hCoV-19/Qatar/QA-WCMQ_FD17571355/2020	EPI_ISL_1714346
hCoV-19/Qatar/QA-WCMQ_FD17571356/2020	EPI_ISL_1714347
hCoV-19/Qatar/QA-WCMQ_FD17571357/2020	EPI_ISL_1714348

hCoV-19/Qatar/QA-WCMQ_FD17571358/2020	EPI_ISL_1714349
hCoV-19/Qatar/QA-WCMQ_FD17571359/2020	EPI_ISL_1714350
hCoV-19/Qatar/QA-QU_15-2-D2/2020	EPI_ISL_1713394
hCoV-19/Qatar/QA-QU_15-2-D3/2020	EPI_ISL_1713395
hCoV-19/Qatar/QA-QU_15-2-E1/2020	EPI_ISL_1713396
hCoV-19/Qatar/QA-QU_16-C10/2020	EPI_ISL_1713440
hCoV-19/Qatar/QA-QU_16-C11/2020	EPI_ISL_1713441
hCoV-19/Qatar/QA-QU_16-C12/2020	EPI_ISL_1713442
hCoV-19/Qatar/QA-QU_16-D1/2020	EPI_ISL_1713449
hCoV-19/Qatar/QA-QU_16-D2/2020	EPI_ISL_1713450
hCoV-19/Qatar/QA-QU_16-D3/2020	EPI_ISL_1713451
hCoV-19/Qatar/QA-QU_16-D4/2020	EPI_ISL_1713452
hCoV-19/Qatar/QA-QU_16-D5/2020	EPI_ISL_1713453
hCoV-19/Qatar/QA-QU_16-D6/2020	EPI_ISL_1713454
hCoV-19/Qatar/QA-QU_16-D7/2020	EPI_ISL_1713455
hCoV-19/Qatar/QA-QU_15-3-B9/2020	EPI_ISL_1713404
hCoV-19/Qatar/QA-QU_15-3-G3/2020	EPI_ISL_1713410
hCoV-19/Qatar/QA-QU_17-D10/2020	EPI_ISL_1713485
hCoV-19/Qatar/QA-QU_17-D9/2020	EPI_ISL_1713489
hCoV-19/Qatar/QA-QU_15-3-D9/2021	EPI_ISL_1713407
hCoV-19/Qatar/QA-QU_15-3-E8/2021	EPI_ISL_1713408
hCoV-19/Qatar/QA-QU_15-3-F7/2021	EPI_ISL_1713409
hCoV-19/Qatar/QA-QU_16-C7/2021	EPI_ISL_1713448
hCoV-19/Qatar/QA-QU_15-3-B2/2021	EPI_ISL_1713403
hCoV-19/Qatar/QA-QU_15-3-C12/2021	EPI_ISL_1713405
hCoV-19/Qatar/QA-QU_15-3-D11/2021	EPI_ISL_1713406
hCoV-19/Qatar/QA-QU_17-D12/2021	EPI_ISL_1713486
hCoV-19/Qatar/QA-QU_17-E1/2021	EPI_ISL_1713490
hCoV-19/Qatar/QA-QU_17-E2/2021	EPI_ISL_1713494
hCoV-19/Qatar/QA-QU_17-H1/2021	EPI_ISL_1713510
hCoV-19/Qatar/QA-QU_17-H4/2021	EPI_ISL_1713512
hCoV-19/Qatar/QA-QU_17-H5/2021	EPI_ISL_1713513
hCoV-19/Qatar/QA-QU_17-H6/2021	EPI_ISL_1713514
hCoV-19/Qatar/QA-QU_17-H7/2021	EPI_ISL_1713515
hCoV-19/Qatar/QA-QU_17-E4/2021	EPI_ISL_1713495
hCoV-19/Qatar/QA-QU_17-E5/2021	EPI_ISL_1713496
hCoV-19/Qatar/QA-QU_17-H2/2021	EPI_ISL_1713511
hCoV-19/Qatar/QA-QU_17-E6/2021	EPI_ISL_1713497
hCoV-19/Qatar/QA-QU_17-G4/2021	EPI_ISL_1713508
hCoV-19/Qatar/QA-QU_17-E10/2021	EPI_ISL_1713491

hCoV-19/Qatar/QA-QU_17-H8/2021	EPI_ISL_1713516
hCoV-19/Qatar/QA-QU_16-F12/2021	EPI_ISL_1713465
hCoV-19/Qatar/QA-QU_17-E11/2021	EPI_ISL_1713492
hCoV-19/Qatar/QA-QU_16-A1/2021	EPI_ISL_1713421
hCoV-19/Qatar/QA-QU_16-A10/2021	EPI_ISL_1713422
hCoV-19/Qatar/QA-QU_16-A2/2021	EPI_ISL_1713423
hCoV-19/Qatar/QA-QU_16-A3/2021	EPI_ISL_1713424
hCoV-19/Qatar/QA-QU_16-A4/2021	EPI_ISL_1713425
hCoV-19/Qatar/QA-QU_16-A5/2021	EPI_ISL_1713426
hCoV-19/Qatar/QA-QU_16-A6/2021	EPI_ISL_1713427
hCoV-19/Qatar/QA-QU_16-A7/2021	EPI_ISL_1713428
hCoV-19/Qatar/QA-QU_16-A8/2021	EPI_ISL_1713429
hCoV-19/Qatar/QA-QU_16-A9/2021	EPI_ISL_1713430
hCoV-19/Qatar/QA-QU_16-F11/2021	EPI_ISL_1713464
hCoV-19/Qatar/QA-QU_17-E12/2021	EPI_ISL_1713493
hCoV-19/Qatar/QA-QU_17-F1/2021	EPI_ISL_1713498
hCoV-19/Qatar/QA-QU_17-F5/2021	EPI_ISL_1713501
hCoV-19/Qatar/QA-QU_17-F6/2021	EPI_ISL_1713502
hCoV-19/Qatar/QA-QU_17-F10/2021	EPI_ISL_1713499
hCoV-19/Qatar/QA-QU_17-F8/2021	EPI_ISL_1713503
hCoV-19/Qatar/QA-QU_17-F9/2021	EPI_ISL_1713504
hCoV-19/Qatar/QA-QU_17-G1/2021	EPI_ISL_1713505
hCoV-19/Qatar/QA-QU_17-G12/2021	EPI_ISL_1713507
hCoV-19/Qatar/QA-QU_17-F11/2021	EPI_ISL_1713500
hCoV-19/Qatar/QA-QU_17-G10/2021	EPI_ISL_1713506
hCoV-19/Qatar/QA-QU_17-G9/2021	EPI_ISL_1713509
hCoV-19/Qatar/QA-QU_18-H7/2021	EPI_ISL_1713923
hCoV-19/Qatar/QA-QU_18-D10/2021	EPI_ISL_1713891
hCoV-19/Qatar/QA-QU_18-F10/2021	EPI_ISL_1713907
hCoV-19/Qatar/QA-QU_19-1-B11/2021	EPI_ISL_1713938
hCoV-19/Qatar/QA-QU_18-A5/2021	EPI_ISL_1713867
hCoV-19/Qatar/QA-QU_18-A8/2021	EPI_ISL_1713870
hCoV-19/Qatar/QA-QU_18-B6/2021	EPI_ISL_1713877
hCoV-19/Qatar/QA-QU_18-C7/2021	EPI_ISL_1713888
hCoV-19/Qatar/QA-QU_18-D7/2021	EPI_ISL_1713896
hCoV-19/Qatar/QA-QU_18-E7/2021	EPI_ISL_1713903
hCoV-19/Qatar/QA-QU_18-F5/2021	EPI_ISL_1713910
hCoV-19/Qatar/QA-QU_18-G3/2021	EPI_ISL_1713916
hCoV-19/Qatar/QA-QU_18-G8/2021	EPI_ISL_1713918
hCoV-19/Qatar/QA-QU_18-A6/2021	EPI_ISL_1713868
hCoV-19/Qatar/QA-QU_18-B8/2021	EPI_ISL_1713879

hCoV-19/Qatar/QA-QU_18-C6/2021	EPI_ISL_1713887
hCoV-19/Qatar/QA-QU_18-C8/2021	EPI_ISL_1713889
hCoV-19/Qatar/QA-QU_18-D6/2021	EPI_ISL_1713895
hCoV-19/Qatar/QA-QU_18-E3/2021	EPI_ISL_1713899
hCoV-19/Qatar/QA-QU_18-E6/2021	EPI_ISL_1713902
hCoV-19/Qatar/QA-QU_18-E8/2021	EPI_ISL_1713904
hCoV-19/Qatar/QA-QU_18-F3/2021	EPI_ISL_1713908
hCoV-19/Qatar/QA-QU_18-F8/2021	EPI_ISL_1713912
hCoV-19/Qatar/QA-QU_18-H4/2021	EPI_ISL_1713922
hCoV-19/Qatar/QA-QU_19-1-A9/2021	EPI_ISL_1713935
hCoV-19/Qatar/QA-QU_19-1-B10/2020	EPI_ISL_1713937
hCoV-19/Qatar/QA-QU_18-A4/2021	EPI_ISL_1713866
hCoV-19/Qatar/QA-QU_18-A7/2021	EPI_ISL_1713869
hCoV-19/Qatar/QA-QU_18-A9/2021	EPI_ISL_1713871
hCoV-19/Qatar/QA-QU_18-B4/2021	EPI_ISL_1713875
hCoV-19/Qatar/QA-QU_18-B5/2021	EPI_ISL_1713876
hCoV-19/Qatar/QA-QU_18-B7/2021	EPI_ISL_1713878
hCoV-19/Qatar/QA-QU_18-B9/2021	EPI_ISL_1713880
hCoV-19/Qatar/QA-QU_18-C4/2021	EPI_ISL_1713885
hCoV-19/Qatar/QA-QU_18-C5/2021	EPI_ISL_1713886
hCoV-19/Qatar/QA-QU_18-C9/2021	EPI_ISL_1713890
hCoV-19/Qatar/QA-QU_18-D5/2021	EPI_ISL_1713894
hCoV-19/Qatar/QA-QU_18-E4/2021	EPI_ISL_1713900
hCoV-19/Qatar/QA-QU_18-E5/2021	EPI_ISL_1713901
hCoV-19/Qatar/QA-QU_18-F4/2021	EPI_ISL_1713909
hCoV-19/Qatar/QA-QU_18-F7/2021	EPI_ISL_1713911
hCoV-19/Qatar/QA-QU_18-G5/2021	EPI_ISL_1713917
hCoV-19/Qatar/QA-QU_19-1-A1/2021	EPI_ISL_1713925
hCoV-19/Qatar/QA-QU_18-B1/2021	EPI_ISL_1713872
hCoV-19/Qatar/QA-QU_18-C1/2021	EPI_ISL_1713881
hCoV-19/Qatar/QA-QU_18-D9/2021	EPI_ISL_1713897
hCoV-19/Qatar/QA-QU_18-F1/2021	EPI_ISL_1713906
hCoV-19/Qatar/QA-QU_18-H2/2021	EPI_ISL_1713921
hCoV-19/Qatar/QA-QU_19-1-A10/2021	EPI_ISL_1713926
hCoV-19/Qatar/QA-QU_18-A2/2021	EPI_ISL_1713865
hCoV-19/Qatar/QA-QU_18-C10/2021	EPI_ISL_1713882
hCoV-19/Qatar/QA-QU_18-E9/2021	EPI_ISL_1713905
hCoV-19/Qatar/QA-QU_18-F9/2021	EPI_ISL_1713913
hCoV-19/Qatar/QA-QU_18-G10/2021	EPI_ISL_1713914
hCoV-19/Qatar/QA-QU_18-H10/2021	EPI_ISL_1713919
hCoV-19/Qatar/QA-QU_18-A10/2021	EPI_ISL_1713863

hCoV-19/Qatar/QA-QU_18-A12/2021	EPI_ISL_1713864
hCoV-19/Qatar/QA-QU_18-B11/2021	EPI_ISL_1713873
hCoV-19/Qatar/QA-QU_18-B12/2021	EPI_ISL_1713874
hCoV-19/Qatar/QA-QU_18-C11/2021	EPI_ISL_1713883
hCoV-19/Qatar/QA-QU_18-C12/2021	EPI_ISL_1713884
hCoV-19/Qatar/QA-QU_18-D11/2021	EPI_ISL_1713892
hCoV-19/Qatar/QA-QU_18-D12/2021	EPI_ISL_1713893
hCoV-19/Qatar/QA-QU_18-E12/2021	EPI_ISL_1713898
hCoV-19/Qatar/QA-QU_18-G12/2021	EPI_ISL_1713915
hCoV-19/Qatar/QA-QU_18-H11/2021	EPI_ISL_1713920
hCoV-19/Qatar/QA-QU_18-H9/2021	EPI_ISL_1713924
hCoV-19/Qatar/QA-QU_19-1-A4/2021	EPI_ISL_1713931
hCoV-19/Qatar/QA-QU_19-1-B7/2021	EPI_ISL_1713944
hCoV-19/Qatar/QA-QU_19-1-B4/2021	EPI_ISL_1713941
hCoV-19/Qatar/QA-QU_19-1-B5/2021	EPI_ISL_1713942
hCoV-19/Qatar/QA-QU_18-2-B1/2021	EPI_ISL_1713568
hCoV-19/Qatar/QA-QU_18-2-E2/2021	EPI_ISL_1713575
hCoV-19/Qatar/QA-QU_18-2-E3/2021	EPI_ISL_1713576
hCoV-19/Qatar/QA-QU_18-2-F3/2021	EPI_ISL_1713578
hCoV-19/Qatar/QA-QU_18-2-B4/2021	EPI_ISL_1713570
hCoV-19/Qatar/QA-QU_18-2-B5/2021	EPI_ISL_1713571
hCoV-19/Qatar/QA-QU_18-2-E6/2021	EPI_ISL_1713577
hCoV-19/Qatar/QA-QU_18-2-F5/2021	EPI_ISL_1713579
hCoV-19/Qatar/QA-QU_18-2-H6/2021	EPI_ISL_1713582
hCoV-19/Qatar/QA-QU_19-1-B3/2021	EPI_ISL_1713940
hCoV-19/Qatar/QA-QU_19-1-B8/2021	EPI_ISL_1713945
hCoV-19/Qatar/QA-QU_19-1-B1/2021	EPI_ISL_1713936
hCoV-19/Qatar/QA-QU_18-2-2-B1/2021	EPI_ISL_1713567
hCoV-19/Qatar/QA-QU_18-2-B7/2021	EPI_ISL_1713572
hCoV-19/Qatar/QA-QU_18-2-G11/2021	EPI_ISL_1713580
hCoV-19/Qatar/QA-QU_18-2-H9/2021	EPI_ISL_1713583
hCoV-19/Qatar/QA-QU_19-1-A5/2021	EPI_ISL_1713932
hCoV-19/Qatar/QA-QU_19-1-A7/2021	EPI_ISL_1713934
hCoV-19/Qatar/QA-QU_19-1-B9/2021	EPI_ISL_1713946
hCoV-19/Qatar/QA-QU_18-2-B11/2021	EPI_ISL_1713569
hCoV-19/Qatar/QA-QU_18-2-C11/2021	EPI_ISL_1713573
hCoV-19/Qatar/QA-QU_18-2-C12/2021	EPI_ISL_1713574
hCoV-19/Qatar/QA-QU_18-2-G9/2021	EPI_ISL_1713581
hCoV-19/Qatar/QA-QU_19-1-A2/2021	EPI_ISL_1713929
hCoV-19/Qatar/QA-QU_18-3-A11/2021	EPI_ISL_1713585
hCoV-19/Qatar/QA-QU_18-3-A12/2021	EPI_ISL_1713586

hCoV-19/Qatar/QA-QU_18-3-B12/2021	EPI_ISL_1713592
hCoV-19/Qatar/QA-QU_18-3-D12/2021	EPI_ISL_1713602
hCoV-19/Qatar/QA-QU_18-3-F11/2021	EPI_ISL_1713611
hCoV-19/Qatar/QA-QU_18-3-G11/2021	EPI_ISL_1713617
hCoV-19/Qatar/QA-QU_18-3-H12/2021	EPI_ISL_1713621
hCoV-19/Qatar/QA-QU_18-3-A8/2021	EPI_ISL_1713589
hCoV-19/Qatar/QA-QU_18-3-A9/2021	EPI_ISL_1713590
hCoV-19/Qatar/QA-QU_18-3-B10/2021	EPI_ISL_1713591
hCoV-19/Qatar/QA-QU_18-3-B8/2021	EPI_ISL_1713594
hCoV-19/Qatar/QA-QU_18-3-B9/2021	EPI_ISL_1713595
hCoV-19/Qatar/QA-QU_18-3-C9/2021	EPI_ISL_1713600
hCoV-19/Qatar/QA-QU_18-3-E10/2021	EPI_ISL_1713605
hCoV-19/Qatar/QA-QU_18-3-E8/2021	EPI_ISL_1713608
hCoV-19/Qatar/QA-QU_18-3-F10/2021	EPI_ISL_1713610
hCoV-19/Qatar/QA-QU_18-3-F8/2021	EPI_ISL_1713614
hCoV-19/Qatar/QA-QU_18-3-F9/2021	EPI_ISL_1713615
hCoV-19/Qatar/QA-QU_18-3-G8/2021	EPI_ISL_1713619
hCoV-19/Qatar/QA-QU_18-3-G9/2021	EPI_ISL_1713620
hCoV-19/Qatar/QA-QU_18-3-A6/2021	EPI_ISL_1713588
hCoV-19/Qatar/QA-QU_18-3-C5/2021	EPI_ISL_1713598
hCoV-19/Qatar/QA-QU_18-3-C7/2021	EPI_ISL_1713599
hCoV-19/Qatar/QA-QU_18-3-D6/2021	EPI_ISL_1713604
hCoV-19/Qatar/QA-QU_18-3-E5/2021	EPI_ISL_1713606
hCoV-19/Qatar/QA-QU_18-3-E7/2021	EPI_ISL_1713607
hCoV-19/Qatar/QA-QU_18-3-F5/2021	EPI_ISL_1713612
hCoV-19/Qatar/QA-QU_18-3-F7/2021	EPI_ISL_1713613
hCoV-19/Qatar/QA-QU_19-1-A11/2021	EPI_ISL_1713927
hCoV-19/Qatar/QA-QU_19-1-A6/2021	EPI_ISL_1713933
hCoV-19/Qatar/QA-QU_18-3-A3/2021	EPI_ISL_1713587
hCoV-19/Qatar/QA-QU_18-3-B3/2021	EPI_ISL_1713593
hCoV-19/Qatar/QA-QU_18-3-C3/2021	EPI_ISL_1713597
hCoV-19/Qatar/QA-QU_18-3-D3/2021	EPI_ISL_1713603
hCoV-19/Qatar/QA-QU_18-3-G2/2021	EPI_ISL_1713618
hCoV-19/Qatar/QA-QU_18-3-H2/2021	EPI_ISL_1713622
hCoV-19/Qatar/QA-QU_18-3-H3/2021	EPI_ISL_1713623
hCoV-19/Qatar/QA-QU_18-3-H4/2021	EPI_ISL_1713624
hCoV-19/Qatar/QA-QU_18-3-C2/2021	EPI_ISL_1713596
hCoV-19/Qatar/QA-QU_18-3-F1/2021	EPI_ISL_1713609
hCoV-19/Qatar/QA-QU_18-3-G1/2021	EPI_ISL_1713616
hCoV-19/Qatar/QA-QU_18-4-A11/2021	EPI_ISL_1713626
hCoV-19/Qatar/QA-QU_18-4-D11/2021	EPI_ISL_1713642

hCoV-19/Qatar/QA-QU_18-4-E12/2021	EPI_ISL_1713645
hCoV-19/Qatar/QA-QU_18-4-F12/2021	EPI_ISL_1713651
hCoV-19/Qatar/QA-QU_18-4-H10/2021	EPI_ISL_1713660
hCoV-19/Qatar/QA-QU_18-4-H12/2021	EPI_ISL_1713661
hCoV-19/Qatar/QA-QU_18-3-A1/2021	EPI_ISL_1713584
hCoV-19/Qatar/QA-QU_18-3-D1/2021	EPI_ISL_1713601
hCoV-19/Qatar/QA-QU_18-4-A9/2021	EPI_ISL_1713629
hCoV-19/Qatar/QA-QU_18-4-B10/2021	EPI_ISL_1713630
hCoV-19/Qatar/QA-QU_18-4-C12/2021	EPI_ISL_1713635
hCoV-19/Qatar/QA-QU_18-4-C9/2021	EPI_ISL_1713640
hCoV-19/Qatar/QA-QU_18-4-D10/2021	EPI_ISL_1713641
hCoV-19/Qatar/QA-QU_18-4-E8/2021	EPI_ISL_1713649
hCoV-19/Qatar/QA-QU_18-4-E9/2021	EPI_ISL_1713650
hCoV-19/Qatar/QA-QU_18-4-G9/2021	EPI_ISL_1713659
hCoV-19/Qatar/QA-QU_18-4-H8/2021	EPI_ISL_1713665
hCoV-19/Qatar/QA-QU_18-4-A6/2021	EPI_ISL_1713628
hCoV-19/Qatar/QA-QU_18-4-B8/2021	EPI_ISL_1713633
hCoV-19/Qatar/QA-QU_18-4-C6/2021	EPI_ISL_1713638
hCoV-19/Qatar/QA-QU_18-4-C8/2021	EPI_ISL_1713639
hCoV-19/Qatar/QA-QU_18-4-E6/2021	EPI_ISL_1713648
hCoV-19/Qatar/QA-QU_18-4-F5/2021	EPI_ISL_1713653
hCoV-19/Qatar/QA-QU_18-4-F7/2021	EPI_ISL_1713654
hCoV-19/Qatar/QA-QU_18-4-G6/2021	EPI_ISL_1713657
hCoV-19/Qatar/QA-QU_18-4-G7/2021	EPI_ISL_1713658
hCoV-19/Qatar/QA-QU_18-4-H5/2021	EPI_ISL_1713664
hCoV-19/Qatar/QA-QU_18-4-A4/2021	EPI_ISL_1713627
hCoV-19/Qatar/QA-QU_18-4-B2/2021	EPI_ISL_1713631
hCoV-19/Qatar/QA-QU_18-4-B5/2021	EPI_ISL_1713632
hCoV-19/Qatar/QA-QU_18-4-C3/2021	EPI_ISL_1713636
hCoV-19/Qatar/QA-QU_18-4-C4/2021	EPI_ISL_1713637
hCoV-19/Qatar/QA-QU_18-4-D2/2021	EPI_ISL_1713643
hCoV-19/Qatar/QA-QU_18-4-E3/2021	EPI_ISL_1713646
hCoV-19/Qatar/QA-QU_18-4-E4/2021	EPI_ISL_1713647
hCoV-19/Qatar/QA-QU_18-4-F2/2021	EPI_ISL_1713652
hCoV-19/Qatar/QA-QU_18-4-G2/2021	EPI_ISL_1713655
hCoV-19/Qatar/QA-QU_18-4-G4/2021	EPI_ISL_1713656
hCoV-19/Qatar/QA-QU_18-4-H2/2021	EPI_ISL_1713662
hCoV-19/Qatar/QA-QU_18-4-H3/2021	EPI_ISL_1713663
hCoV-19/Qatar/QA-QU_18-4-A1/2021	EPI_ISL_1713625
hCoV-19/Qatar/QA-QU_18-4-C1/2021	EPI_ISL_1713634
hCoV-19/Qatar/QA-QU_18-4-E1/2021	EPI_ISL_1713644

hCoV-19/Qatar/QA-QU_18-5-2-A2/2021	EPI_ISL_1713691
hCoV-19/Qatar/QA-QU_18-5-2-A6/2021	EPI_ISL_1713693
hCoV-19/Qatar/QA-QU_18-5-2-A9/2021	EPI_ISL_1713694
hCoV-19/Qatar/QA-QU_18-5-2-B9/2021	EPI_ISL_1713696
hCoV-19/Qatar/QA-QU_18-5-2-C2/2021	EPI_ISL_1713698
hCoV-19/Qatar/QA-QU_18-5-2-H6/2021	EPI_ISL_1713712
hCoV-19/Qatar/QA-QU_18-5-2-C1/2021	EPI_ISL_1713697
hCoV-19/Qatar/QA-QU_18-5-2-E5/2021	EPI_ISL_1713704
hCoV-19/Qatar/QA-QU_18-5-2-E8/2021	EPI_ISL_1713705
hCoV-19/Qatar/QA-QU_18-5-2-F5/2021	EPI_ISL_1713707
hCoV-19/Qatar/QA-QU_18-5-2-F6/2021	EPI_ISL_1713708
hCoV-19/Qatar/QA-QU_18-5-2-A10/2021	EPI_ISL_1713689
hCoV-19/Qatar/QA-QU_18-5-2-A12/2021	EPI_ISL_1713690
hCoV-19/Qatar/QA-QU_18-5-2-A4/2021	EPI_ISL_1713692
hCoV-19/Qatar/QA-QU_18-5-2-D6/2021	EPI_ISL_1713701
hCoV-19/Qatar/QA-QU_18-5-2-H2/2021	EPI_ISL_1713710
hCoV-19/Qatar/QA-QU_18-5-2-H5/2021	EPI_ISL_1713711
hCoV-19/Qatar/QA-QU_18-5-2-B4/2021	EPI_ISL_1713695
hCoV-19/Qatar/QA-QU_18-5-2-D2/2021	EPI_ISL_1713699
hCoV-19/Qatar/QA-QU_18-5-2-D3/2021	EPI_ISL_1713700
hCoV-19/Qatar/QA-QU_18-5-2-E2/2021	EPI_ISL_1713702
hCoV-19/Qatar/QA-QU_18-5-2-E4/2021	EPI_ISL_1713703
hCoV-19/Qatar/QA-QU_18-5-2-G4/2021	EPI_ISL_1713709
hCoV-19/Qatar/QA-QU_18-5-3-A5/2021	EPI_ISL_1713714
hCoV-19/Qatar/QA-QU_18-5-3-A8/2021	EPI_ISL_1713717
hCoV-19/Qatar/QA-QU_18-5-3-B7/2021	EPI_ISL_1713719
hCoV-19/Qatar/QA-QU_18-5-3-B9/2021	EPI_ISL_1713720
hCoV-19/Qatar/QA-QU_18-5-3-C10/2021	EPI_ISL_1713721
hCoV-19/Qatar/QA-QU_18-5-3-C8/2021	EPI_ISL_1713722
hCoV-19/Qatar/QA-QU_18-5-3-A6/2021	EPI_ISL_1713715
hCoV-19/Qatar/QA-QU_18-5-3-A7/2021	EPI_ISL_1713716
hCoV-19/Qatar/QA-QU_18-5-3-D4/2021	EPI_ISL_1713724
hCoV-19/Qatar/QA-QU_18-5-3-E1/2021	EPI_ISL_1713725
hCoV-19/Qatar/QA-QU_18-5-3-E10/2021	EPI_ISL_1713726
hCoV-19/Qatar/QA-QU_18-5-3-G7/2021	EPI_ISL_1713733
hCoV-19/Qatar/QA-QU_18-5-3-E4/2021	EPI_ISL_1713727
hCoV-19/Qatar/QA-QU_18-5-3-E9/2021	EPI_ISL_1713729
hCoV-19/Qatar/QA-QU_18-5-3-G1/2021	EPI_ISL_1713730
hCoV-19/Qatar/QA-QU_18-5-3-G3/2021	EPI_ISL_1713732
hCoV-19/Qatar/QA-QU_18-5-2-F4/2021	EPI_ISL_1713706
hCoV-19/Qatar/QA-QU_18-5-3-A12/2021	EPI_ISL_1713713

hCoV-19/Qatar/QA-QU_18-5-3-B1/2021	EPI_ISL_1713718
hCoV-19/Qatar/QA-QU_18-5-3-D11/2021	EPI_ISL_1713723
hCoV-19/Qatar/QA-QU_18-5-3-E6/2021	EPI_ISL_1713728
hCoV-19/Qatar/QA-QU_18-5-3-G11/2021	EPI_ISL_1713731
hCoV-19/Qatar/QA-QU_18-5-1-C10/2021	EPI_ISL_1713668
hCoV-19/Qatar/QA-QU_18-5-1-D10/2021	EPI_ISL_1713673
hCoV-19/Qatar/QA-QU_18-5-1-D12/2021	EPI_ISL_1713674
hCoV-19/Qatar/QA-QU_18-5-1-E11/2021	EPI_ISL_1713676
hCoV-19/Qatar/QA-QU_18-5-1-E12/2021	EPI_ISL_1713677
hCoV-19/Qatar/QA-QU_18-5-1-G12/2021	EPI_ISL_1713684
hCoV-19/Qatar/QA-QU_18-5-1-E9/2021	EPI_ISL_1713680
hCoV-19/Qatar/QA-QU_18-5-1-F7/2021	EPI_ISL_1713683
hCoV-19/Qatar/QA-QU_18-5-1-G9/2021	EPI_ISL_1713685
hCoV-19/Qatar/QA-QU_18-5-1-H6/2021	EPI_ISL_1713687
hCoV-19/Qatar/QA-QU_18-5-1-H9/2021	EPI_ISL_1713688
hCoV-19/Qatar/QA-QU_18-5-1-C2/2021	EPI_ISL_1713669
hCoV-19/Qatar/QA-QU_18-5-1-C3/2021	EPI_ISL_1713670
hCoV-19/Qatar/QA-QU_18-5-1-C6/2021	EPI_ISL_1713671
hCoV-19/Qatar/QA-QU_18-5-1-D2/2021	EPI_ISL_1713675
hCoV-19/Qatar/QA-QU_18-5-1-E5/2021	EPI_ISL_1713679
hCoV-19/Qatar/QA-QU_18-5-1-F6/2021	EPI_ISL_1713682
hCoV-19/Qatar/QA-QU_18-5-1-A4/2021	EPI_ISL_1713666
hCoV-19/Qatar/QA-QU_18-5-1-B2/2021	EPI_ISL_1713667
hCoV-19/Qatar/QA-QU_18-5-1-D1/2021	EPI_ISL_1713672
hCoV-19/Qatar/QA-QU_18-5-1-E4/2021	EPI_ISL_1713678
hCoV-19/Qatar/QA-QU_18-5-1-F1/2021	EPI_ISL_1713681
hCoV-19/Qatar/QA-QU_18-5-1-H1/2021	EPI_ISL_1713686
hCoV-19/Qatar/QA-QU_18-6-C3/2021	EPI_ISL_1713735
hCoV-19/Qatar/QA-QU_18-6-C4/2021	EPI_ISL_1713736
hCoV-19/Qatar/QA-QU_18-6-F2/2021	EPI_ISL_1713744
hCoV-19/Qatar/QA-QU_18-6-F3/2021	EPI_ISL_1713745
hCoV-19/Qatar/QA-QU_18-6-F4/2021	EPI_ISL_1713746
hCoV-19/Qatar/QA-QU_18-6-H4/2021	EPI_ISL_1713753
hCoV-19/Qatar/QA-QU_18-6-C7/2021	EPI_ISL_1713737
hCoV-19/Qatar/QA-QU_18-6-D7/2021	EPI_ISL_1713740
hCoV-19/Qatar/QA-QU_18-6-G4/2021	EPI_ISL_1713749
hCoV-19/Qatar/QA-QU_18-6-G5/2021	EPI_ISL_1713750
hCoV-19/Qatar/QA-QU_18-6-H6/2021	EPI_ISL_1713754
hCoV-19/Qatar/QA-QU_18-6-C9/2021	EPI_ISL_1713738
hCoV-19/Qatar/QA-QU_18-6-D1/2021	EPI_ISL_1713739
hCoV-19/Qatar/QA-QU_18-6-D9/2021	EPI_ISL_1713741

hCoV-19/Qatar/QA-QU_18-6-F8/2021	EPI_ISL_1713747
hCoV-19/Qatar/QA-QU_18-6-G9/2021	EPI_ISL_1713751
hCoV-19/Qatar/QA-QU_18-6-H9/2021	EPI_ISL_1713755
hCoV-19/Qatar/QA-QU_18-6-B10/2021	EPI_ISL_1713734
hCoV-19/Qatar/QA-QU_18-6-E12/2021	EPI_ISL_1713742
hCoV-19/Qatar/QA-QU_18-6-F12/2021	EPI_ISL_1713743
hCoV-19/Qatar/QA-QU_18-6-G12/2021	EPI_ISL_1713748
hCoV-19/Qatar/QA-QU_18-6-H11/2021	EPI_ISL_1713752
hCoV-19/Qatar/QA-QU_18-7-H12/2021	EPI_ISL_1713775
hCoV-19/Qatar/QA-QU_18-9-A1/2021	EPI_ISL_1713820
hCoV-19/Qatar/QA-QU_18-7-C9/2021	EPI_ISL_1713763
hCoV-19/Qatar/QA-QU_18-7-D9/2021	EPI_ISL_1713764
hCoV-19/Qatar/QA-QU_18-7-F11/2021	EPI_ISL_1713769
hCoV-19/Qatar/QA-QU_18-7-G11/2021	EPI_ISL_1713773
hCoV-19/Qatar/QA-QU_18-7-H10/2021	EPI_ISL_1713774
hCoV-19/Qatar/QA-QU_18-7-A8/2021	EPI_ISL_1713758
hCoV-19/Qatar/QA-QU_18-7-B6/2021	EPI_ISL_1713760
hCoV-19/Qatar/QA-QU_18-7-B8/2021	EPI_ISL_1713761
hCoV-19/Qatar/QA-QU_18-7-E8/2021	EPI_ISL_1713767
hCoV-19/Qatar/QA-QU_18-7-F6/2021	EPI_ISL_1713772
hCoV-19/Qatar/QA-QU_18-7-H8/2021	EPI_ISL_1713777
hCoV-19/Qatar/QA-QU_18-7-B2/2021	EPI_ISL_1713759
hCoV-19/Qatar/QA-QU_18-7-E5/2021	EPI_ISL_1713766
hCoV-19/Qatar/QA-QU_18-7-F1/2021	EPI_ISL_1713768
hCoV-19/Qatar/QA-QU_18-7-F2/2021	EPI_ISL_1713770
hCoV-19/Qatar/QA-QU_18-7-H2/2021	EPI_ISL_1713776
hCoV-19/Qatar/QA-QU_18-7-A2/2021	EPI_ISL_1713756
hCoV-19/Qatar/QA-QU_18-7-A4/2021	EPI_ISL_1713757
hCoV-19/Qatar/QA-QU_18-7-C4/2021	EPI_ISL_1713762
hCoV-19/Qatar/QA-QU_18-7-E3/2021	EPI_ISL_1713765
hCoV-19/Qatar/QA-QU_18-7-F3/2021	EPI_ISL_1713771
hCoV-19/Qatar/QA-QU_18-8-B2/2021	EPI_ISL_1713784
hCoV-19/Qatar/QA-QU_18-8-E2/2021	EPI_ISL_1713803
hCoV-19/Qatar/QA-QU_18-8-G1/2021	EPI_ISL_1713811
hCoV-19/Qatar/QA-QU_18-8-H1/2021	EPI_ISL_1713816
hCoV-19/Qatar/QA-QU_18-8-A5/2021	EPI_ISL_1713780
hCoV-19/Qatar/QA-QU_18-8-A6/2021	EPI_ISL_1713781
hCoV-19/Qatar/QA-QU_18-8-B5/2021	EPI_ISL_1713785
hCoV-19/Qatar/QA-QU_18-8-C4/2021	EPI_ISL_1713790
hCoV-19/Qatar/QA-QU_18-8-D3/2021	EPI_ISL_1713797
hCoV-19/Qatar/QA-QU_18-8-E1/2021	EPI_ISL_1713801

hCoV-19/Qatar/QA-QU_18-8-E3/2021	EPI_ISL_1713804
hCoV-19/Qatar/QA-QU_18-8-F3/2021	EPI_ISL_1713807
hCoV-19/Qatar/QA-QU_18-8-F5/2021	EPI_ISL_1713808
hCoV-19/Qatar/QA-QU_18-8-A11/2021	EPI_ISL_1713779
hCoV-19/Qatar/QA-QU_18-8-A7/2021	EPI_ISL_1713782
hCoV-19/Qatar/QA-QU_18-8-B6/2021	EPI_ISL_1713786
hCoV-19/Qatar/QA-QU_18-8-C6/2021	EPI_ISL_1713791
hCoV-19/Qatar/QA-QU_18-8-C8/2021	EPI_ISL_1713792
hCoV-19/Qatar/QA-QU_18-8-D7/2021	EPI_ISL_1713798
hCoV-19/Qatar/QA-QU_18-8-D8/2021	EPI_ISL_1713799
hCoV-19/Qatar/QA-QU_18-8-E6/2021	EPI_ISL_1713805
hCoV-19/Qatar/QA-QU_18-8-F6/2021	EPI_ISL_1713809
hCoV-19/Qatar/QA-QU_18-8-F7/2021	EPI_ISL_1713810
hCoV-19/Qatar/QA-QU_18-8-G8/2021	EPI_ISL_1713814
hCoV-19/Qatar/QA-QU_18-8-A10/2021	EPI_ISL_1713778
hCoV-19/Qatar/QA-QU_18-8-B1/2021	EPI_ISL_1713783
hCoV-19/Qatar/QA-QU_18-8-B9/2021	EPI_ISL_1713787
hCoV-19/Qatar/QA-QU_18-8-C12/2021	EPI_ISL_1713789
hCoV-19/Qatar/QA-QU_18-8-D1/2021	EPI_ISL_1713793
hCoV-19/Qatar/QA-QU_18-8-D11/2021	EPI_ISL_1713795
hCoV-19/Qatar/QA-QU_18-8-D12/2021	EPI_ISL_1713796
hCoV-19/Qatar/QA-QU_18-8-D9/2021	EPI_ISL_1713800
hCoV-19/Qatar/QA-QU_18-8-E8/2021	EPI_ISL_1713806
hCoV-19/Qatar/QA-QU_18-8-G9/2021	EPI_ISL_1713815
hCoV-19/Qatar/QA-QU_18-8-H6/2021	EPI_ISL_1713818
hCoV-19/Qatar/QA-QU_18-8-C10/2021	EPI_ISL_1713788
hCoV-19/Qatar/QA-QU_18-8-D10/2021	EPI_ISL_1713794
hCoV-19/Qatar/QA-QU_18-8-E11/2021	EPI_ISL_1713802
hCoV-19/Qatar/QA-QU_18-8-G11/2021	EPI_ISL_1713812
hCoV-19/Qatar/QA-QU_18-8-G12/2021	EPI_ISL_1713813
hCoV-19/Qatar/QA-QU_18-8-H10/2021	EPI_ISL_1713817
hCoV-19/Qatar/QA-QU_18-8-H9/2021	EPI_ISL_1713819
hCoV-19/Qatar/QA-QU_18-9-A12/2021	EPI_ISL_1713823
hCoV-19/Qatar/QA-QU_18-9-D11/2021	EPI_ISL_1713836
hCoV-19/Qatar/QA-QU_18-9-D12/2021	EPI_ISL_1713837
hCoV-19/Qatar/QA-QU_18-9-E12/2021	EPI_ISL_1713843
hCoV-19/Qatar/QA-QU_18-9-F11/2021	EPI_ISL_1713851
hCoV-19/Qatar/QA-QU_18-9-G11/2021	EPI_ISL_1713857
hCoV-19/Qatar/QA-QU_18-9-A11/2021	EPI_ISL_1713822
hCoV-19/Qatar/QA-QU_18-9-B2/2021	EPI_ISL_1713826
hCoV-19/Qatar/QA-QU_18-9-D1/2021	EPI_ISL_1713834

hCoV-19/Qatar/QA-QU_18-9-D10/2021	EPI_ISL_1713835
hCoV-19/Qatar/QA-QU_18-9-D9/2021	EPI_ISL_1713840
hCoV-19/Qatar/QA-QU_18-9-E1/2021	EPI_ISL_1713841
hCoV-19/Qatar/QA-QU_18-9-E10/2021	EPI_ISL_1713842
hCoV-19/Qatar/QA-QU_18-9-F1/2021	EPI_ISL_1713849
hCoV-19/Qatar/QA-QU_18-9-F10/2021	EPI_ISL_1713850
hCoV-19/Qatar/QA-QU_18-9-F9/2021	EPI_ISL_1713854
hCoV-19/Qatar/QA-QU_18-9-G1/2021	EPI_ISL_1713855
hCoV-19/Qatar/QA-QU_18-9-G10/2021	EPI_ISL_1713856
hCoV-19/Qatar/QA-QU_18-9-H10/2021	EPI_ISL_1713859
hCoV-19/Qatar/QA-QU_18-9-H9/2021	EPI_ISL_1713862
hCoV-19/Qatar/QA-QU_18-9-A10/2021	EPI_ISL_1713821
hCoV-19/Qatar/QA-QU_18-9-A7/2021	EPI_ISL_1713825
hCoV-19/Qatar/QA-QU_18-9-B3/2021	EPI_ISL_1713827
hCoV-19/Qatar/QA-QU_18-9-B7/2021	EPI_ISL_1713830
hCoV-19/Qatar/QA-QU_18-9-B9/2021	EPI_ISL_1713831
hCoV-19/Qatar/QA-QU_18-9-C6/2021	EPI_ISL_1713832
hCoV-19/Qatar/QA-QU_18-9-C9/2021	EPI_ISL_1713833
hCoV-19/Qatar/QA-QU_18-9-D6/2021	EPI_ISL_1713839
hCoV-19/Qatar/QA-QU_18-9-E3/2021	EPI_ISL_1713845
hCoV-19/Qatar/QA-QU_18-9-E4/2021	EPI_ISL_1713846
hCoV-19/Qatar/QA-QU_18-9-H8/2021	EPI_ISL_1713861
hCoV-19/Qatar/QA-QU_18-9-B5/2021	EPI_ISL_1713829
hCoV-19/Qatar/QA-QU_18-9-D5/2021	EPI_ISL_1713838
hCoV-19/Qatar/QA-QU_18-9-E2/2021	EPI_ISL_1713844
hCoV-19/Qatar/QA-QU_18-9-E5/2021	EPI_ISL_1713847
hCoV-19/Qatar/QA-QU_18-9-E7/2021	EPI_ISL_1713848
hCoV-19/Qatar/QA-QU_18-9-F2/2021	EPI_ISL_1713852
hCoV-19/Qatar/QA-QU_18-9-F6/2021	EPI_ISL_1713853
hCoV-19/Qatar/QA-QU_18-9-G2/2021	EPI_ISL_1713858
hCoV-19/Qatar/QA-QU_18-9-H6/2021	EPI_ISL_1713860
hCoV-19/Qatar/QA-QU_18-10-A11/2021	EPI_ISL_1713520
hCoV-19/Qatar/QA-QU_18-10-A6/2021	EPI_ISL_1713525
hCoV-19/Qatar/QA-QU_18-10-B3/2021	EPI_ISL_1713533
hCoV-19/Qatar/QA-QU_18-9-A6/2021	EPI_ISL_1713824
hCoV-19/Qatar/QA-QU_18-10-B1/2021	EPI_ISL_1713529
hCoV-19/Qatar/QA-QU_18-10-B2/2021	EPI_ISL_1713532
hCoV-19/Qatar/QA-QU_18-10-C1/2021	EPI_ISL_1713539
hCoV-19/Qatar/QA-QU_18-10-C2/2021	EPI_ISL_1713542
hCoV-19/Qatar/QA-QU_18-10-C3/2021	EPI_ISL_1713543
hCoV-19/Qatar/QA-QU_18-10-C4/2021	EPI_ISL_1713544

hCoV-19/Qatar/QA-QU_18-10-C8/2021	EPI_ISL_1713548
hCoV-19/Qatar/QA-QU_18-9-B4/2021	EPI_ISL_1713828
hCoV-19/Qatar/QA-QU_18-10-A8/2021	EPI_ISL_1713527
hCoV-19/Qatar/QA-QU_18-10-A9/2021	EPI_ISL_1713528
hCoV-19/Qatar/QA-QU_18-10-C7/2021	EPI_ISL_1713547
hCoV-19/Qatar/QA-QU_18-10-A10/2021	EPI_ISL_1713519
hCoV-19/Qatar/QA-QU_18-10-A12/2021	EPI_ISL_1713521
hCoV-19/Qatar/QA-QU_18-10-A7/2021	EPI_ISL_1713526
hCoV-19/Qatar/QA-QU_18-10-B12/2020	EPI_ISL_1713531
hCoV-19/Qatar/QA-QU_18-10-C6/2021	EPI_ISL_1713546
hCoV-19/Qatar/QA-QU_18-10-C9/2021	EPI_ISL_1713549
hCoV-19/Qatar/QA-QU_18-10-A5/2021	EPI_ISL_1713524
hCoV-19/Qatar/QA-QU_18-10-B9/2021	EPI_ISL_1713538
hCoV-19/Qatar/QA-QU_18-10-B5/2021	EPI_ISL_1713535
hCoV-19/Qatar/QA-QU_18-10-B6/2021	EPI_ISL_1713536
hCoV-19/Qatar/QA-QU_18-10-B7/2021	EPI_ISL_1713537
hCoV-19/Qatar/QA-QU_18-10-C12/2021	EPI_ISL_1713541
hCoV-19/Qatar/QA-QU_18-10-C5/2021	EPI_ISL_1713545
hCoV-19/Qatar/QA-QU_18-10-B10/2021	EPI_ISL_1713530
hCoV-19/Qatar/QA-QU_18-10-B4/2021	EPI_ISL_1713534
hCoV-19/Qatar/QA-QU_18-10-D11/2021	EPI_ISL_1713552
hCoV-19/Qatar/QA-QU_18-10-D4/2021	EPI_ISL_1713555
hCoV-19/Qatar/QA-QU_18-10-D7/2021	EPI_ISL_1713557
hCoV-19/Qatar/QA-QU_18-10-D8/2021	EPI_ISL_1713558
hCoV-19/Qatar/QA-QU_18-10-D9/2021	EPI_ISL_1713559
hCoV-19/Qatar/QA-QU_18-10-D2/2021	EPI_ISL_1713553
hCoV-19/Qatar/QA-QU_18-10-D3/2021	EPI_ISL_1713554
hCoV-19/Qatar/QA-QU_18-10-E8/2021	EPI_ISL_1713563
hCoV-19/Qatar/QA-QU_18-10-F1/2021	EPI_ISL_1713564
hCoV-19/Qatar/QA-QU_18-10-F11/2021	EPI_ISL_1713566
hCoV-19/Qatar/QA-QU_16-2-A5/2020	EPI_ISL_1713420
hCoV-19/Qatar/QA-QU_16-B11/2020	EPI_ISL_1713431
hCoV-19/Qatar/QA-QU_16-B12/2020	EPI_ISL_1713432
hCoV-19/Qatar/QA-QU_16-B4/2020	EPI_ISL_1713433
hCoV-19/Qatar/QA-QU_16-B5/2020	EPI_ISL_1713434
hCoV-19/Qatar/QA-QU_16-B6/2020	EPI_ISL_1713435
hCoV-19/Qatar/QA-QU_16-B7/2020	EPI_ISL_1713436
hCoV-19/Qatar/QA-QU_16-B8/2020	EPI_ISL_1713437
hCoV-19/Qatar/QA-QU_16-B9/2020	EPI_ISL_1713438
hCoV-19/Qatar/QA-QU_16-C1/2020	EPI_ISL_1713439
hCoV-19/Qatar/QA-QU_16-C2/2020	EPI_ISL_1713443

hCoV-19/Qatar/QA-QU_16-C3/2020	EPI_ISL_1713444
hCoV-19/Qatar/QA-QU_16-C4/2020	EPI_ISL_1713445
hCoV-19/Qatar/QA-QU_16-C5/2020	EPI_ISL_1713446
hCoV-19/Qatar/QA-QU_16-C6/2020	EPI_ISL_1713447
hCoV-19/Qatar/QA-QU_19-1-A3/2020	EPI_ISL_1713930
hCoV-19/Qatar/QA-QU_19-1-B2/2020	EPI_ISL_1713939
hCoV-19/Qatar/QA-QU_19-1-B6/2020	EPI_ISL_1713943
hCoV-19/Qatar/QA-QU_18-10-A2/2020	EPI_ISL_1713522
hCoV-19/Qatar/QA-QU_18-10-A4/2020	EPI_ISL_1713523
hCoV-19/Qatar/QA-QU_18-10-C10/2020	EPI_ISL_1713540
hCoV-19/Qatar/QA-QU_18-10-D1/2020	EPI_ISL_1713550
hCoV-19/Qatar/QA-QU_18-10-D5/2020	EPI_ISL_1713556
hCoV-19/Qatar/QA-QU_16-2-A2/2020	EPI_ISL_1713418
hCoV-19/Qatar/QA-QU_16-2-A4/2021	EPI_ISL_1713419
hCoV-19/Qatar/QA-QU_19-1-A12/2020	EPI_ISL_1713928
hCoV-19/Qatar/QA-QU_18-10-A1/2020	EPI_ISL_1713518
hCoV-19/Qatar/QA-QU_18-10-D10/2020	EPI_ISL_1713551
hCoV-19/Qatar/QA-QU_18-10-E3/2021	EPI_ISL_1713561
hCoV-19/Qatar/QA-QU_18-10-F10/2021	EPI_ISL_1713565
hCoV-19/Qatar/QA-QU_18-10-E12/2021	EPI_ISL_1713560
hCoV-19/Qatar/QA-QU_18-10-E4/2021	EPI_ISL_1713562
